# Synthesis, Evaluation for Cytotoxicity and Molecular Docking Studies of Benzo[*c*]furan-Chalcones for Potential to Inhibit Tubulin Polymerization and/or EGFR-Tyrosine Kinase Phosphorylation

**DOI:** 10.3390/ijms19092552

**Published:** 2018-08-28

**Authors:** Malose J. Mphahlele, Marole M. Maluleka, Nishal Parbhoo, Sibusiso T. Malindisa

**Affiliations:** 1 Department of Chemistry, College of Science, Engineering and Technology, University of South Africa, Private Bag X06, Florida 1710, South Africa; 2 Department of Life & Consumer Sciences, College of Agriculture and Environmental Sciences, University of South Africa, Private Bag X06, Florida 1710, South Africa; parbhn1@unisa.ac.za (N.P.); malinst@unisa.ac.za (S.T.M.)

**Keywords:** benzofuran-chalcones, cytotoxicity, apoptosis, tubulin, EGFR, molecular docking

## Abstract

A series of 2-arylbenzo[*c*]furan-chalcone hybrids **3a**–**y** have been synthesized and evaluated for antiproliferative effects against the human breast cancer (MCF-7) cell line and for its potential to induce apoptosis and also to inhibit tubulin polymerization and/or epidermal growth factor receptor-tyrosine kinase (EGFR-TK) phosphorylation. Most of these compounds exhibited moderate to significant antigrowth effects in vitro against the MCF-7 cell line when compared to the reference standard actinomycin D. The capabilities of the most cytotoxic benzofuran-chalcone hybrids **3b** and **3i**, to induce apoptosis, have been evaluated by Annexin V-Cy3 SYTOX staining and caspase-3 activation. The experimental and molecular docking results suggest that the title compounds have the potential to exhibit inhibitory effects against tubulin polymerization and epidermal growth factor receptor tyrosine kinase (EGFR-TK) phosphorylation. The modeled structures of representative compounds displayed hydrophobic interactions as well as hydrogen and/or halogen bonding with the protein residues. These interactions are probably responsible for the observed increased binding affinity for the two receptors and their significant antigrowth effect against the MCF-7 cell line.

## 1. Introduction

**Figure 1:**
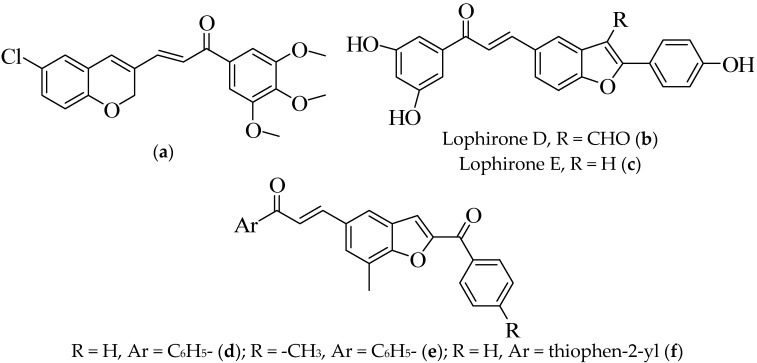
Compound (**a**) and examples of biologically relevant linear benzofuran-chalcone hybrids.

Breast cancer is one of the leading causes of death in females in both developing and developed countries globally [1]. Among the common chemotherapeutic agents currently used in the treatment of metastatic breast cancer are the antimitotic drugs, which bind primarily to β-tubulin [2]. Chalcones are the precursors of flavonoids and isoflavonoids that exhibit a wide range of biological properties including anticancer activity [3,4]. Several mechanisms of action in chalcone-based compounds, such as anticancer agents, have been identified and these include the induction of apoptosis, DNA and mitochondrial damage, inhibition of angiogenesis, tubulin inhibition, kinase inhibition, and drug efflux protein activities, or a combination of some of these mechanisms [5]. Previous investigations suggest that the replacement of one or both phenyl rings of chalcones with a heteroaryl moiety results in heterocycle-appended chalcone hybrids with enhanced and synergistic anticancer properties [6,7]. (*E*)-3-(6-Chloro-2*H*-chromen-3-yl)-1-(3,4,5-trimethoxyphenyl)prop-2-en-1-one (**a**), shown in Figure 1, for example, was previously found to bind directly to tubulin and perturb microtubule stability and the function of the spindle apparatus [8]. To date, there have been numbers of bioassay-guided searches for benzofurans [9] and chalcone derivatives [10] as potential microtubule targeting agents. We considered the anticancer properties of the 2-arylbenzofurans [8,11,12] and their photophysical properties as potential photosensitizing anticancer agents [11] and decided to link the two pharmacophores in order to comprise benzofuran-chalcone hybrids. The most common framework of benzofuran-chalcone hybrids, that we encountered in the literature, have the α,β-unsaturated carbonyl moiety attached to the C-2 or C-3 position of the heterocyclic ring–either through the carbonyl [13,14,15] or through the β-carbon [16]. Very few examples of the benzo[*b*]furan-chalcone hybrids, in which an α,β-unsaturated moiety is appended to the fused benzo ring, existed in the literature [17,18,19]. Lophirones D (**b**) and E (**c**), shown in Figure 1, are examples of the naturally occurring benzofuran-chalcones previously isolated from the stem bark of *Lophira lanceolate* (Ochnaceae), and various parts of this plant are used in tropical Africa for the treatment of toothaches (Cameroon), liver infections (Togo), female sterility, and coughs (Nigeria) [18]. The synthetic analogues of the general structure **d**–**f** (Figure 1), on the other hand, have been found to significantly reduce the aggregation of amyloid-beta peptide and to increase the acetylcholine (Ach) levels along with the overall availability of Ach at the synaptic junction [19]. Moreover, these compounds were also found to decrease acetylcholinesterase (AchE) levels, reduce oxidative stress in the worms (*Caenorhabditis elegans*), lower lipid content, and to provide protection against chemically induced cholinergic neurodegeneration [19].

A literature search revealed that the benzo[*c*]furan-chalcones have not been prepared before and such angular hybrids do not feature in the recent review by Zhuang et al. on the chalcone hybrids of medicinal importance [4]. We were able to synthesize these angular benzofuran-appended chalcone hybrids using the 5-bromo-2-hydroxy-3-iodochalcones as substrates for the tandem palladium catalyzed alkynylation and *endo-dig* Csp-O cycloisomerization. The compounds have been evaluated for their antigrowth effect in vitro against the human breast cancer (MCF-7) cell line. Mechanistic anticancer investigations were performed by testing the potential of representative hybrids to induce apoptosis in the MCF-7 cells and for their capability to inhibit tubulin polymerization and/or EGFR-TK phosphorylation. We selected representative compounds and docked them into the colchicine-binding site of tubulin and the adenosine triphosphate (ATP) binding pocket of the EGFR.

## 2. Results and Discussion

### 2.1. Chemistry

**Scheme 1:**
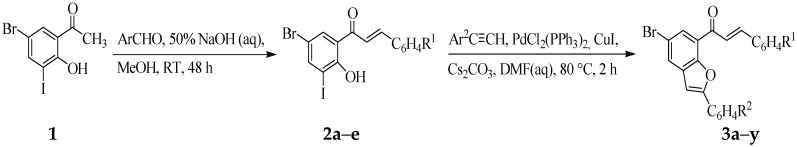
Synthesis of the angular benzofuran-chalcone hybrids **3a**–**y**.

The pathways leading to the synthesis of the benzofuran-appended chalcones described in this investigation are depicted in Scheme 1 with the description of the corresponding substitution pattern outlined in Table 1 below. The analytical data and the corresponding nuclear magnetic resonance (NMR) spectra (^1^H- and ^13^C-NMR) of compounds **2a**–**e** and **3a**–**y** have been included in Figure S1 in the Supplementary Materials. 5-Bromo-2-hydroxy-3-iodoacetophenone **1**, used as a precursor in this investigation was prepared in 64% yield in a one-pot operation involving initial bromination of 2-hydoxyacetophenone with **1** equivalent of *N*-bromosuccinimide (NBS) in acetic acid under reflux for 1.5 h, followed by iodination with *N*-iodosuccinimide (NIS) in acetic acid under reflux for an additional 1.5 h. This mixed 3,5-dihalogenated-2-hydroxyacetophenone was prepared before by treatment of the commercially available 5-bromo-2-hydroxyacetophenone with pyridinium iodochloride (1 equivalent) in methanol under reflux for 2 h [20]. Compound **1** was subjected to the Claisen–Schmidt condensation with benzaldehyde derivatives under basic conditions in methanol at room temperature (RT) for 48 h. We isolated the corresponding 5-bromo-2-hydroxy-3-iodochalcones **2a**–**e** in high yields by aqueous work-up and recrystallization. The *E*-geometry around the vinylic framework of these chalcones was confirmed by the two sets of doublets around δ = 7.48 and 7.89 ppm, with coupling constant values *J_trans_* = 15.5 Hz, which correspond to the H-α and H-β, respectively. The 2-alkyl/aryl-substituted benzofurans were previously synthesized via the palladium catalyzed cross coupling-heteroannulation of the 2-bromo/iodophenol derivatives with terminal acetylenes [21,22,23]. It has, however, been observed before that the 2-bromo/iodophenol, substituted with an α,β-unsaturated carbonyl moiety fails to undergo Sonogashira cross-coupling with terminal alkynes when tetrakis(triphenylphosphine)palladium(0) (PdPPh_3_)_4_ was used as the active Pd(0) source [24]. We required a more reactive palladium(0) catalyst source that could affect sequential Sonogashira cross-coupling and the subsequent heteroannulation in order to construct a benzofuran moiety in a one-pot operation. We decided to use the on the commercially available dichlorobis(triphenyl)phosphine(II) (PdCl_2_(PPh_3_)_2_) as the catalyst source since the Pd(0) complex (Pd(0)(PPh_3_)_2_Cl^–^), generated from its reduction, is known to be more than 30 times faster in the oxidative-addition step than that formed from Pd(0)(PPh_3_)_4_ [25]. The 5-bromo-2-hydroxy-3-iodochalcones **2a**–**e** were subjected to the Sonogashira cross-coupling with arylacetylenes in the presence of PdCl_2_(PPh_3_)_2_-CuI catalyst mixture with a cesium carbonate as a base in aqueous dimethyl formamide (DMF) under reflux for 3 h. We isolated, by aqueous work-up and purification by silica gel column chromatography, the compounds characterized by using a combination of NMR, infrared (IR), and mass spectrometric techniques such as the benzofuran-chalcone hybrids **3a**–**y** in reasonable yields. The molecular ion region of the mass spectra of these angular benzofuran-chalcone hybrids revealed the presence of the M^+^ and M^+2^ peaks in the ratio 1:1, which is typical for compounds containing the ^79^Br and ^81^Br isotopes. The observed site selectivity of Csp^2^-Csp bond formation, through the substitution of iodine that is attributed to the difference in Csp^2^-halogen bond strength, which facilitates cross-coupling via the weaker Csp^2^-I bond in the presence of other halogen atoms.

**Figure 2:**
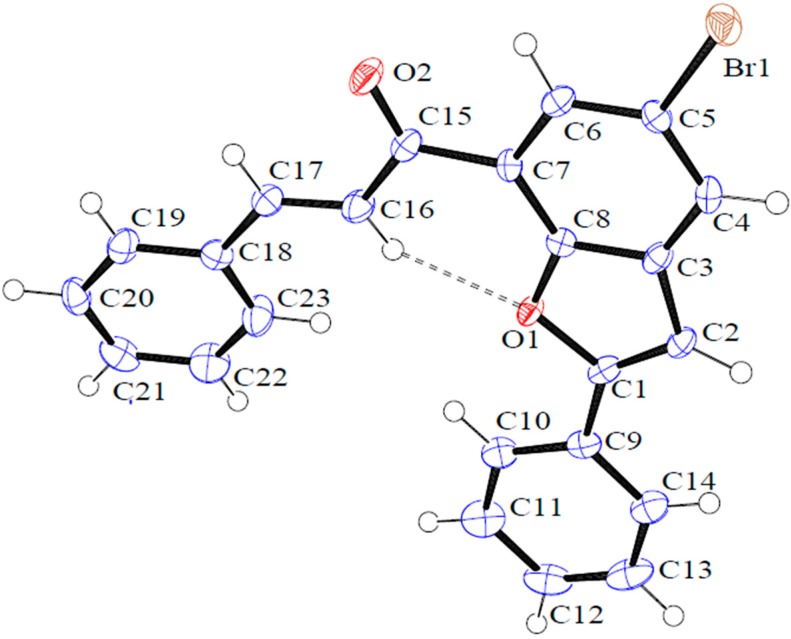
Oak Ridge Thermal Ellipsoid Plot (ORTEP) diagram of **3a**. Displacement ellipsoids are drawn at the 50% probability level and H atoms are shown as small spheres of arbitrary radii.

**Figure 3:**
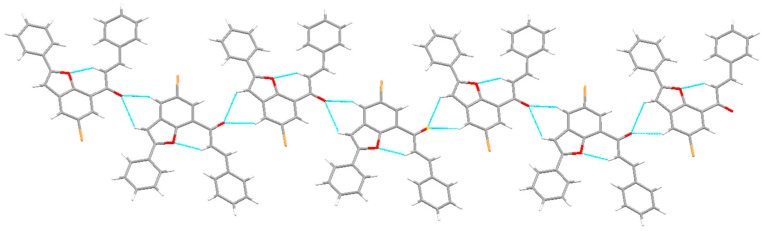
Packing diagram of **3a** showing the 1D ribbon.

The structure of these compounds and the *E*-geometry around the olefinic framework were distinctly confirmed by single crystal X-ray diffraction (XRD) analysis of compound **3a** (Figure 2) [26]. Compound **3a** crystallizes in the P2_1_2_1_2_1_ space group with each asymmetric unit containing one molecule of **3a**. In the asymmetric unit, there is intramolecular hydrogen bonding between the oxygen acceptor (O1) and the α-hydrogen bond donor [d(C(16)-H(16)···O(1) = 2.24 Å], which forms a $S_{1}^{1}$(6) ring (Figure 2). As shown in Figure 3, the molecule packs into a one-dimensional ribbon via bifurcated hydrogen bonding from the oxygen acceptor (O2) and the C-H···O hydrogen bond donors (H2 and H4).

We decided to evaluate the benzofuran-appended chalcone hybrids **3a**–**y** for their potential antiproliferative properties in vitro against the breast cancer (MCF-7) cell line. The structure activity relationship (SAR) of these compounds has been studied with respect to the substitution patterns on the phenyl rings of the chalcone moiety (R^1^) and the benzofuran framework (R^2^).

### 2.2. Biological Studies

#### 2.2.1. Cytotoxicity Studies and SAR Analysis

Compounds **3a**–**y** were grouped into five series, namely, **3a**–**e**, **3f**–**j**, **3k**–**o**, **3p**–**t**, and **3u**–**y** (Table 2) and were evaluated for growth inhibitory activity against the MCF-7 cells using the well-established 3-[4,5-dimethylthiazol-2-yl]-2,5-diphenyltetrazolium bromide (MTT) assay with actinomycin D, which is a well-known chemotherapeutic compound used as the reference drug. Actinomycin D (Act. D) is a DNA interacting transcription blocker with anticancer activity working as a cytotoxic inducer of apoptosis against various tumor cells [27]. The cytotoxic activities of the tested compounds against the MCF-7 cell line have been expressed as the IC_50_ values in μM against actinomycin D (Table 2). Within the series **3a**–**e**, only the compounds **3b**, **3d** and **3e** were substituted with a phenyl ring on the benzofuran moiety and a 4-fluorophenyl, 4-methoxyphenyl or 4-trifluoromethoxyphenyl group on the chalcone arm exhibited increased cytotoxicity against the MCF-7 cells, in comparison to the reference standard (IC_50_ = 37.82 µM) with the IC_50_ values of 0.55, 22.78 and 0.59 µM, respectively. The replacement of the phenyl ring with a 2-(4-fluorophenyl) group on the benzofuran moiety in series **3f**–**j** resulted in reduced cytotoxicity for compounds **3g** and **3h** that were substituted with a 4-halogenophenyl ring on the 2-position of the chalcone framework. However, a combination of the 2-(4-fluorophenyl) group on the benzofuran moiety and a phenyl ring on position-2 of the chalcone arm resulted in significant cytotoxicity for compound **3f** (IC_50_ = 35.81 µM), as compared to actinomycin D. Compounds **3i** and **3j**, substituted with a 4-fluorophenyl ring on the benzofuran moiety and a 4-methoxyphenyl or 4-trifluoromethoxyphenyl group on the chalcone arm, exhibited increased cytotoxicity against the MCF-7 cell line with the IC_50_ values of 3.55 × 10^−4^ µM and 16.00 µM, respectively. In the series **3k**–**o**, substituted with a 2-(3-fluorophenyl) ring on the benzofuran moiety, only compound **3l** containing a 4-fluorophenyl group on the chalcone arm was found to be inactive against this cell line. Compounds **3k**, **3m**, and **3o**, substituted with a phenyl, 4-chlorophenyl and 4-trifluoromethoxyphenyl group on the chalcone fragment, on the other hand, exhibited increased cytotoxicity than the reference standard with IC_50_ values of 23.01, 14.75, and 7.87 µM, respectively. The 4-methoxyphenyl substituted derivative **3n** exhibited comparable activity to the reference standard with an IC_50_ value of 39.25 µM. A 3-chlorophenyl group at the 2-position of the benzofuran framework in series **3p**–**t** was found to be beneficial for the cytotoxicity of compounds **3p**, **3q**, **3s**, and **3t** substituted with a phenyl, 4-fluorophenyl, a 4-methoxyphenyl and a 4-trifluoromethoxy group on the chalcone moiety with IC_50_ values of 12.63, 23.82, 2.69, and 26.62 µM, respectively. However, a combination of the 3-chlorophenyl group on the benzofuran framework and a 4-chlorophenyl group on the chalcone moiety resulted in the lack of cytotoxicity for compound **3r**. The presence of a 4-methoxyphenyl group at the C-2 position of the benzofuran moiety in the series **3u**–**y**, on the other hand, resulted in relatively reduced cytotoxicity against the MCF-7 cell line when compared to the other series. However, a combination of the 4-methoxyphenyl group at the C-2 position of the benzofuran and a 4-fluorophenyl or 4-chlorophenyl ring on the chalcone arm resulted in increased cytotoxicity for compounds **3v** (IC_50_ = 28.43 µM) and **3w** (IC_50_ = 30.62 µM) when compared to the reference standard.

Chalcones have been reported to act as microtubule destabilizing agents that prevent tubulin from polymerizing into microtubules by binding to the colchicine-binding site [28]. It has also been demonstrated by Yang et al. that (*E*)-1-(2,4-dihydroxyphenyl)-3-(3,4-dihydroxyphenyl)prop-2-en-1-one (butein) the retarded epidermal growth factor receptor (EGFR) tyrosine kinase phosphorylation at a micromolar level [29]. Its inhibition was competitive against ATP in human hepatocellular carcinoma HepG2 cells, which suggested the ATP binding site to be the potential binding pocket for this chalcone [29]. The inhibition of tubulin polymerization activity [30] or EGFR-TK activity [31] are regarded as the most promising approaches for innovative therapeutic strategies in cancer treatment. We considered the observed cytotoxic effects of the majority of compounds **3** against the EGFR-positive MCF-7 cell line [32] and the various mechanisms of action displayed by chalcone-based compounds as anticancer agents [5]—we decided to evaluate these compounds for their potential to induce apoptosis and to inhibit tubulin polymerization and/or EGFR-TK phosphorylation.

#### 2.2.2. Evaluation of Cell Death Pathway by Annexin V-Cy3 SYTOX Staining Assay

**Figure 4:**
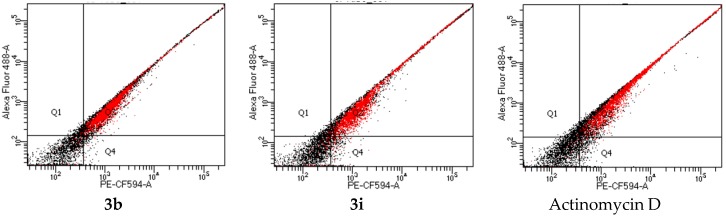
Annexin V–Cy3 SYTOX staining of MCF-7 cells treated with **3b**, **3i**, and actinomycin D at 1 µM concentration for 48 h, respectively.

Several distinctive modes of cell death such as apoptosis, necrosis, autophagy, and cornification exist and are characterized by differences in their morphology and their biochemical changes [33]. A chalcone derivative, (*E*)-3-(3-chlorophenyl)-1-phenylprop-2-en-1-one, has previously been found to exhibit significant antiproliferative activity against various cancer cell lines and to induce apoptosis through the intrinsic and extrinsic pathways in MCF-7 cells [34]. This literature precedent encouraged us to evaluate the most cytotoxic compounds, **3b** and **3i**, for their potential to induce apoptosis in the MCF-7 cells. We employed the Annexin V-Cy3 SYTOX apoptosis assay kit to distinguish between viable cells (Annexin V-negative; PI-negative), early apoptotic cells (Annexin V-positive; PI-negative), late apoptotic cells (Annexin V-positive; PI-positive) and unviable or necrotic cells (Annexin V-negative; PI-positive). The cells were analyzed by the flow cytometry, which is an important tool to evaluate the molecular and morphological events that take place during cell death and cell proliferation. MCF-7 cells exposed to the 1 µM concentration of compounds **3b**, **3i**, and actinomycin D for 48 h resulted in the accumulation of a significant amount of apoptotic cells (Figure 4 and Table 3). Compound **3b**, which is the second most cytotoxic derivative among the twenty-five compound, induced increased apoptosis against the MCF-7 cells when compared to **3i** and actinomycin D.

**Figure 5:**
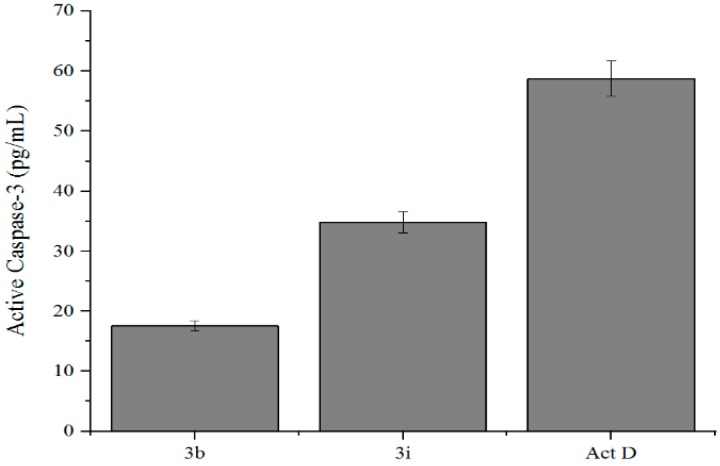
Effects of compounds **3b**, **3i**, and actinomycin D (Act. D) at 1 µM on human Caspase-3 (Asp175) activity in MCF-7 cells, respectively.

Activation of caspases (caspase-2, 3, 6, 7, 8, 9, and 10) is a major biochemical feature that plays a central role in the morphological changes associated with apoptosis [35]. Consequently, we evaluated compounds **3b** and **3i** for their potential to induce caspase-3 activation in the MCF-7 cells. The results from caspase-3 activation (Figure 5) indicate that compound **3b**, which induced increased apoptosis according to the Annexin staining, does not induce significant caspase activation when compared to **3i** and actinomycin D. Since the breast cancer cell line MCF-7 lacks a functional caspase-3 gene product [36], apoptosis induction in these cells by these compounds may be through other molecular pathways that do not involve caspase activation.

#### 2.2.3. Effect of Compounds **3a**–**y** on Tubulin Polymerization

In order to test whether the five series of benzofuran-chalcone hybrids, **3a**–**e**, **3f**–**j**, **3k**–**o**, **3p**–**t**, and **3u**–**y** inhibit tubulin polymerization, we evaluated them for their potential to interact with the microtubule system using colchicine as a reference standard in a cell-free in vitro assay. The corresponding IC_50_ values for selected derivatives (**3b**, **3e**, **3i**, **3j**, **3o**, **3p**, **3s**, and **3v**) are listed in Table 4 below (refer to Table S1, Supplementary Materials for a complete list of IC_50_ values for **3a**–**y**). Compounds **3a**–**e** generally exhibited less inhibitory effects against tubulin polymerization when compared to colchicine (IC_50_ = 9.88 × 10^−2^ µM). The most cytotoxic compounds against the MCF-7 cells in this series, namely, **3b** and **3e**, exhibited relatively poor inhibitory effects against tubulin polymerization with IC_50_ values of 26.5 µM and 25.4 µM, respectively. Literature precedents revealed that some highly cytotoxic compounds were not necessarily potent inhibitors of tubulin polymerization and vice versa [37,38]. Moreover in our view, the observed poor tubulin inhibitory effects for hybrids **3b** and **3e** may suggest that inhibiting tubulin polymerization is not the only way for their antitumor activity. Compounds **3i** and **3j** within the series **3f**–**j** were found to exhibit increased inhibitory effect against tubulin polymerization with IC_50_ values 5.51 × 10^−5^ µM and 8.85 × 10^−3^ µM, respectively. This trend somewhat correlates with their anti-proliferative effect against the MCF-7 cell line. A combination of a 2-(3-fluorophenyl) group on the chalcone arm and a 2-(4-trifluoromethoxyphenyl) group on the benzofuran scaffold in compound **3o** resulted in increased inhibitory effects against tubulin polymerization (IC_50_ = 1.76 × 10^−4^ µM) when compared to the other derivatives within the series **3k**–**o**. Moderate activity was observed for compound **3n** substituted with a 4-methoxyphenyl group on the benzofuran framework. The presence of a 2-(3-chlorophenyl) group on the chalcone moiety, in combination on the benzofuran framework with either a phenyl ring in **3p** or the 4-methoxyphenyl group in **3s,** resulted in significant inhibitory effects against tubulin polymerization as compared to the other derivatives within the series **3p**–**t**. Within the series **3u**–**y**, compound **3v**, substituted with a 2-(4-fluorophenyl) group on the benzofuran framework and a 2-(4-methoxyphenyl) substituent on the chalcone arm, was found to retard (IC_50_ = 1.62 × 10^−2^ µM) tubulin polymerization more so than the other derivatives.

Since chalcone-based compounds exhibit anticancer activity via several mechanisms of action, including kinase inhibition [5], we decided to evaluate the most cytotoxic compounds from each series against the MCF-7 cell line for their potential to inhibit EGFR-TK phosphorylation.

#### 2.2.4. Evaluation of Compounds **3b**, **3i**, **3o**, **3p**, and **3v** for Potential to Inhibit EGFR-TK Phosphorylation

We selected the most cytotoxic benzofuran-appended hybrids from each series, namely, **3b**, **3j**, **3o**, **3p** and **3v**, and evaluated these compounds for their potential to inhibit EGFR-tyrosine kinase phosphorylation against actinomycin D and gefitinib. Actinomycin D has previously been found to block Shc/Grb2 interaction in EGFR-overexpressed NIH3T3 (SAA) and B104-1-1 (neuroblast-transformed NIH3T3) cells, which suggested that it could be used for the treatment of specific tumors caused by EGFR or the ErbB-2 oncogene [39]. Gefitinib, on the other hand, is a selective inhibitor of the EGFR-TK and it inhibits tumour pathogenesis, metastasis, and angiogenesis, and also promotes apoptosis [40]. The test compounds and the two reference standards were evaluated for inhibitory effects against EGFR-tyrosine kinase phosphorylation and the corresponding IC_50_ values are represented in Table 5. The results of this assay revealed that the title compounds also exhibit significant inhibitory effect against EGFR-TK phosphorylation when compared to gefitinib. Compound **3b** exhibited an increased cytotoxicity and apoptotic effect but poor anti-tubulin activity was also found to exhibit significant inhibitory activity against EGFR with an IC_50_ value of 0.17 µM. The most cytotoxic compound **3i** against the MCF-7 cells seems to exhibit inhibitory effects against tubulin polymerization and EGFR-TK phosphorylation. Moderate inhibitory effects against the EGFR were also observed for compounds **3o**, **3p**, and **3v** with IC_50_ values of 0.12, 0.17 and 0.15 µM, respectively.

In order to explore the probable interaction model of inhibitors and enzyme active sites, molecular docking studies were conducted on the representative compounds **3i** and **3o** against two targets, namely, tubulin and EGFR.

### 2.3. Molecular Docking Studies

To help us understand the anticancer activity of compounds **3a**–**y** and guide further structure activity relationship (SAR) studies, we selected compounds **3i** and **3o** and docked them into tubulin and EGFR, respectively.

#### 2.3.1. Molecular Docking of **3i** and **3o** into Tubulin

**Figure 6:**
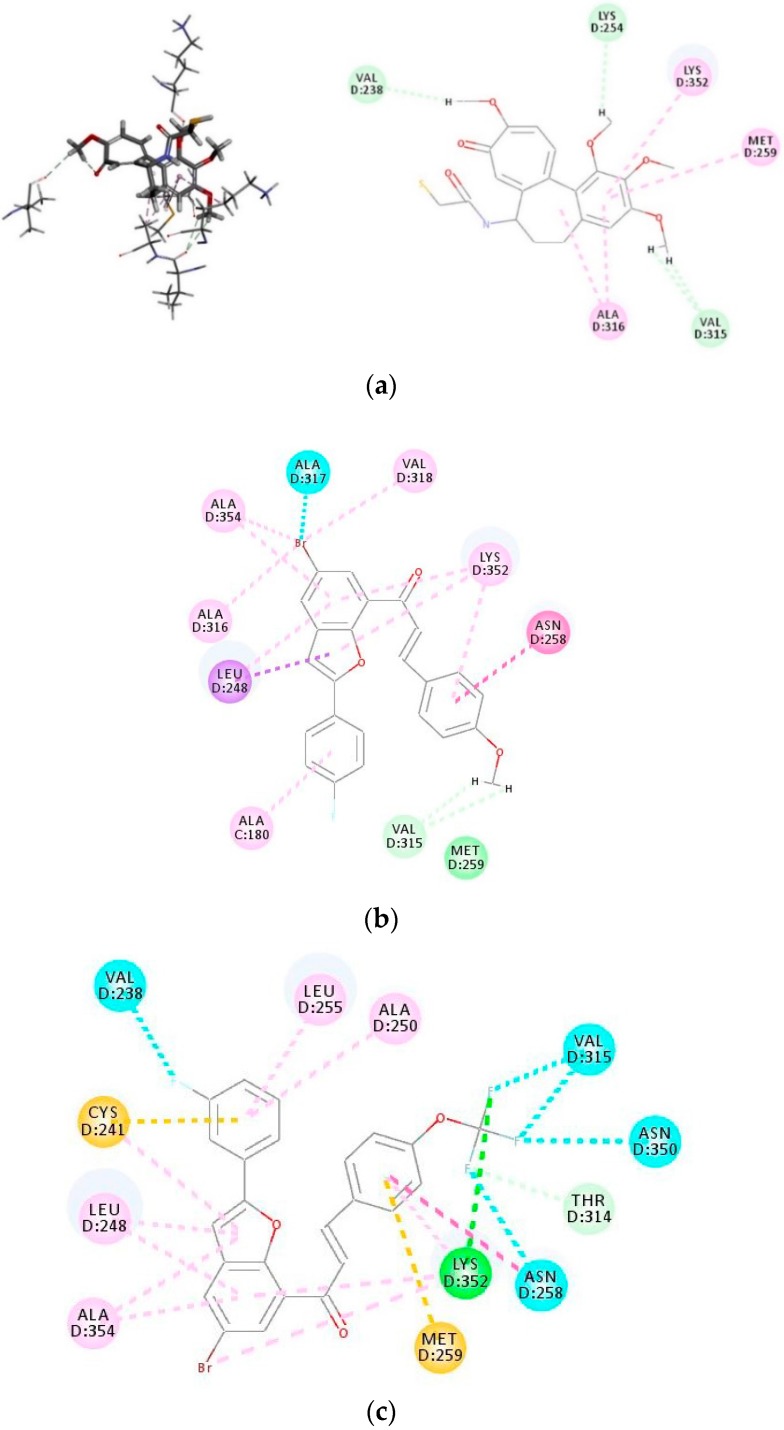
(**a**) 2D interaction diagrams for the binding of tubulin (PDB code: 1SAO) with colchicine; (**b**) the structure of (*E*)-1-(5-Bromo-2-(4-fluorophenyl)benzofuran-7-yl)-3-(4-methoxyphenyl)prop-2-en-1-one (**3i**); (**c**) the structure of (*E*)-1-(5-Bromo-2-(3-fluorophenyl)benzofuran-7-yl)-3-(4-(trifluoromethoxy)phenyl)prop-2-en-1-one (**3o**). Residues are annotated with their three-letter amino acid code.

Compounds **3i** and **3o**, which exhibited increased inhibitory effects against tubulin polymerization and colchicine, were docked into tubulin (PDB ID: 1SAO) and their docked poses are represented in Figure 6 below. The docking poses of colchicine (Figure 6a) and the benzofuran-chalcones **3i** (Figure 6b) and **3o** (Figure 6c) revealed that the hybrids occupy the hydrophobic pocket of the colchicine-binding site at the interface between the α and β-chains. Increased hydrophobic (alkyl, pi-alkyl, pi-sigma, amide-pi) interactions exist between the most active compound **3i** with the side chains of tubulin residues Ala180, Ala316, Ala354, Val318, Lys352, and Leu248 (Figure 6b). The hydrogen atoms of the methoxy group are involved in carbon hydrogen bonding with Val315 (Hb distances = 2.71 and 2.54 Å) and van der Waals interaction with Met259. The bromine atom, on the other hand, is involved in halogen bond interaction with the protein residue Ala317. The docking pose of **3o** (Figure 6c) also revealed the presence of several hydrophobic interactions between the benzofuran moiety and the 4-trifluoromethoxyphenyl group of the chalcone arm with the protein residues Ala250, Ala354, Leu255, and Lys352. There are also pi-sulfur bond interactions between the 3-fluorophenyl and 4-trifluoromethoxyphenyl groups with Cys241 and Met259, respectively. The trifluoromethoxy group is involved in a hydrogen bond interaction with Lys352 (Hb distance = 2.73 Å) and Thr314 (Hb distance = 2.73 Å). The fluorine atom of the 3-fluorophenyl ring interacts with Val238 via halogen bonding while the fluorine atoms of the trifluoromethoxy group are involved in halogen bond interaction with Asn350, Asn358, Val238, and Val315. The modeling studies against tubulin suggest that the hydrophobic interactions and hydrogen and/or halogen bonding with the protein residues helps to stabilize the binding of these compounds in the colchicine-binding domain of α,β-tubulin interface. This probably accounts for the observed significant tubulin polymerization inhibitory effect and the increased cytotoxicity of these compounds against the MCF-7 cell line.

#### 2.3.2. Molecular Docking of Compounds **3i** and **3o** into the EGFR-TK Active Site

**Figure 7:**
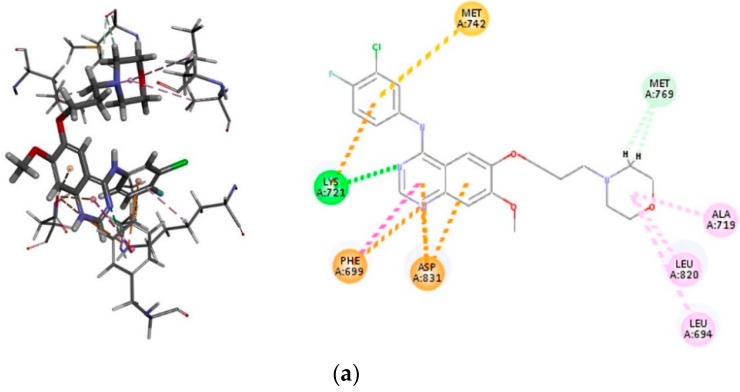

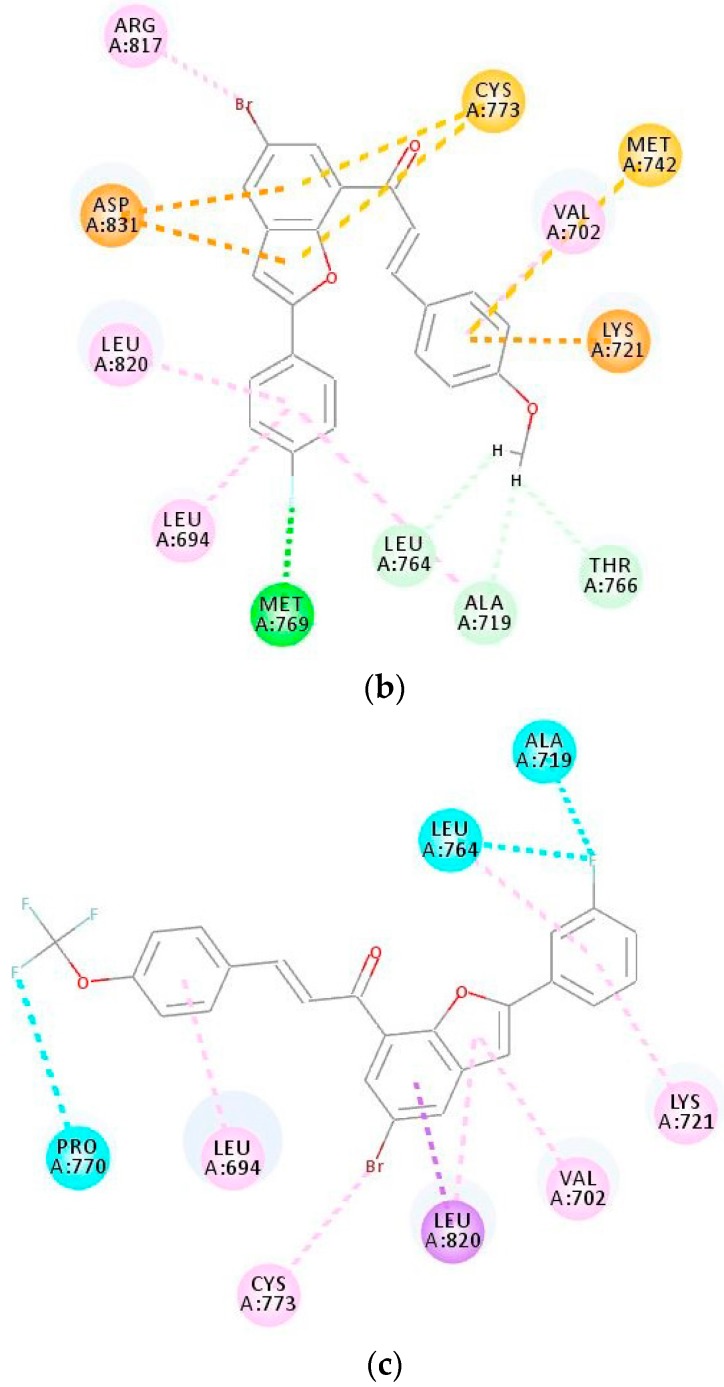
(**a**) 2D interaction diagrams for the binding of EGFR (PDB code 1M17) with gefitinib; (**b**) the structure of (*E*)-1-(5-Bromo-2-(4-fluorophenyl)benzofuran-7-yl)-3-(4-methoxyphenyl)prop-2-en-1-one (**3i**); (**c**) the structure of *E*)-1-(5-Bromo-2-(3-fluorophenyl)benzofuran-7-yl)-3-(4-(trifluoromethoxy)phenyl)prop-2-en-1-one (**3o**). Residues are annotated with their three-letter amino acid code.

In the last part of this investigation, we evaluated whether compounds **3i** and **3o** could be docked into the ATP binding site of EGFR. According to the pharmacophore model of the ATP binding pocket of EGFR, it consists of five regions that are conserved throughout the protein kinases and these include adenine region, hydrophobic region I and II, phosphate binding region, and the sugar pocket [41]. The protein residues Val702, Met742, and Leu764 form part of the hydrophobic region I, whereas the hydrophobic residues such as Leu694, Leu768, Pro770, Phe771, and Leu820 comprise the hydrophobic region II. There are two acidic residues (Glu738 and Asp831) located on the helix-αC and a phosphate binding region along the sugar pocket, whereas the basic residue Lys721 is located near the phosphate binding region that formed the salt-bridge to Glu738 in the helix-αC. Compounds **3i** and **3o** were docked into EGFR (PDB ID: 1M17) and their docking poses were compared with that of the selective EGFR inhibitor—that is, gefitinib (Figure 7a). The important protein residues in the hydrophobic pocket of the ATP-competitive site that interacts with **3i** are Leu694, Leu820, Arg817, and Val702 (Figure 7b). The benzofuran moiety is also involved in pi-sulfur interaction with Cyst773 and pi-sulfur interaction also exists between the methoxyphenyl group on the chalcone arm with Met743. The Lys721 residue establishes a cation-interaction with the 4-methoxyphenyl ring of **3i**. There is also a pi-anion interaction between the benzofuran core of **3i** and carboxylic side chain of Asp831 located on the phosphate-binding region along the sugar pocket. Carbon-hydrogen bond interactions, which are the most common kinase-inhibitor interactions exist between Leu764 (Hb distance = 2.66 Å), Ala719 (Hb distance = 2.52 Å) and Thr766 (Hb distance = 2.43 Å) with the methoxy group of **3i**. The fluorine atom on the 2-phenyl ring of the benzofuran scaffold is involved in hydrogen bonding with the NH backbone of Met769 (Met769-NH···F distance = 2.18 Å), which is known to hydrogen bond to N-1 of the adenine ring of ATP and help to fix it in the binding pocket. This interaction, in our view, would account for the increased inhibitory effect of this compound against the EGFR and therefore its increased cytotoxicity against the EGFR-positive MCF-7 cell line. The docking pose of compound **3o** (Figure 7c) reveals the presence of hydrophobic (alkyl, pi-alkyl and pi-sigma) interactions with the protein residues Leu764, Leu694, Cys773, Lys721, Val702, and Leu820. Val702, Leu820, and Ala719 are located near the gatekeeper residue, Thr766, and its location is envisaged to control the access of an inhibitor to the hydrophobic pocket of the ATP-competitive site [42]. The Lys721 in the ATP-binding site of EGFR is considered to be one of the key residues to decide the biological activity of the EGFR. The fluorine atom on the 4-fluorophenyl group is involved in halogen bonding with Ala719 and Leu764 and additional halogen bonding exists between the trifluoromethoxy group and the protein residue Pro770. Halogen-bonds are known to confer the specificity of inhibitors against protein kinases [43]. Moreover, fluorine-containing groups have been found to enhance the efficacy of the molecule by promoting electrostatic interactions with targets, improving cellular membrane permeability due to its lipophilicity, and increasing strength toward oxidative metabolism of the drug [44]. Compounds **3i** and **3o** exhibit increased interactions with the protein residues in the binding pocket of EGFR and a better binding affinity in line with their strong EGFR binding activity—in addition to its significant cytotoxicity against the MCF-7 cells.

## 3. Materials and Methods

### 3.1. General

The melting point values for the prepared compounds were recorded on a Thermocouple digital melting point apparatus. Their IR spectra were recorded as powders by using the thin-film method using a Bruker VERTEX 70 FT-IR Spectrometer (Bruker Optics, Billerica, MA, USA) with a diamond ATR (attenuated total reflectance) accessory. For purification by column chromatography, we employed Merck kieselgel 60 (0.063–0.200 mm) (Merck KGaA, Frankfurt, Germany) as the stationary phase. The proton and carbon-13 NMR spectra were obtained as CDCl_3_ solutions using Varian Mercury 300 MHz NMR spectrometer (Varian Inc., Palo Alto, CA, USA) or Agilent 500 MHz NMR (Agilent Technologies, Oxford, UK) spectrometer and the chemical shifts are quoted relative to the residual proton in the solvent 7.25 ppm and 77.0 ppm for ^1^H and ^13^C NMR spectra, respectively. Low and high-resolution mass spectra were recorded at the University of Stellenbosch Central Analytical Facility and the ionization potential of 70 eV by using Waters Synapt G2 Quadrupole time-of-flight mass spectrometer (Waters Corp., Milford, MA, USA).

### 3.2. One-Pot Synthesis of 5-Bromo-2-hydroxy-3-iodoacetophenone *(**1**)*

A stirred mixture of 2-hydroxyacetophenone (2.00 g, 14.70 mmol) and NBS (2.61 g, 14.70 mmol) in acetic acid (100 mL) was refluxed for 1.5 h. NIS (4.02 g, 17.95 mmol) and acetic acid (50 mL) were added to the reaction mixture and heating was continued for an additional 1.5 h. The mixture was quenched with an ice cold saturated aqueous solution of sodium thiosulfate and the resulting precipitate was filtered and recrystallized from ethanol to afford **1** as a brown solid (3.20 g, 64%), m.p. 111‒112 °C (Lit. [20] 104–105 °C); ν_max_ (ATR) 441, 539, 670, 779, 862, 968, 1077, 1190, 1233, 1362, 1420, 1581, 1650, 3259 cm^−1^; ^1^H NMR (300 MHz, CDCl_3_) 2.65 (3H, s, CH_3_), 7.83 (1H, d, *J* 2.1 Hz, H-4), 8.04 (1H, d, *J* 2.1 Hz, H-6), 13.07 (1H, s, OH); ^13^C NMR (75 MHz, CDCl_3_) 26.4, 87.6, 111.1, 120.2, 133.1, 147.1, 160.2, and 203.2.

### 3.3. Typical Procedure for the Synthesis of Chalcone Derivatives ***2a**–**e***

A mixture of **1** (1.0 equiv.), benzaldehyde derivative (1.2 equiv. of **1**) and KOH (50%) in methanol (9 mL/mmol of **1**) was stirred at room temperature (RT) for 48 h and then quenched with ice-cold water. The resulting precipitate was filtered on a sintered funnel and then recrystallized from ethanol to afford the 2-hydroxychalcone derivative **2** as an orange solid. Compounds **2a**–**e** were prepared in this fashion.

### 3.4. Typical Procedure for the Synthesis of Benzofuran-Chalcone Hydrids ***3a**–**y***

To a three-necked round-bottom flask equipped with a stirrer bar, condenser, and a rubber septa was added to compound **2** (1 mmol), PdCl_2_(PPh_3_)_2_ (0.05 mmol of **2**), CuI (0.10 mmol), Cs_2_CO_3_ (1.20 mmol) and 10:1 DMF-water (*v*/*v*, 8 mL/mmol of **2**) in sequence. The mixture was purged with nitrogen gas for 20 min. and a balloon filled with nitrogen gas was connected to the top of the condenser. Arylacetylene (1.20 mmol) was added via syringe and the mixture was heated at 80 °C for 2 h. The mixture was quenched with an ice-cold water and the product was extracted with chloroform. The combined organic solutions were washed with brine and dried over anhydrous MgSO_4_. The salt was filtered off and the solvent was evaporated under reduced pressure. The residue was purified by recrystallization from acetone to afford **3a**–**y**.

### 3.5. Materials and Methods for the In Vitro Cytotoxicity Assay

#### 3.5.1. Cell Culturing

The human breast cancer (MCF-7) cell line was maintained in culture flasks containing Dulbecco’s Modified Eagles Medium (DMEM). The media was supplemented with 1% antibiotics (100 µg/mL penicillin, 100 µg/mL streptomycin and 250 µg/L fungizone) and 10% heat-inactivated fetal bovine serum. The cells were grown at 37 °C in a humidified incubator set at 5% CO_2_. The cells were sub-cultured every two-to-three days after the cells had formed a confluent monolayer.

#### 3.5.2. Cytotoxicity against the MCF-7 Cell Line

Cytotoxicity was measured by the 3-[4,5-dimethylthiazol-2-yl]-2,5-diphenyltetrazolium bromide (MTT) method using the Cell Proliferation Kit I (Sigma-Aldrich, St. Louis, MO, USA) according to the method developed by Mosmann et al. [45] The cells were seeded (100 µL) in a 96-well microtiter plates at a concentration of 1 × 10^5^ cells/mL and incubated for 24 h at 37 °C and 5% CO_2_ to allow the cells to attach to the bottom of the wells. The compounds were assessed at 0–100 µM. The control wells included vehicle treated cells exposed to 2% DMSO and the positive control, actinomycin D, with concentrations ranging between 5, 10, 25, and 50, and 100 µM. The microtitre plates were incubated for 48 h. After the 48 h incubation period the MTT reagent (10 µL) was added to a final concentration of 0.5 mg/mL and the plate was further incubated for another 4 h. After 4 h, 100 µL of the solubilisation solution was added to each well and the plate was allowed to stand overnight. Subsequently, the absorbance of the color complex was read at 550 nm with a reference wavelength set at 650 nm using a BIO-TEK Power-Wave XS multi-well plate reader (BioTech Instruments, Midrand, South Africa). The IC_50_ values of the compounds were calculated using OriginPro software (OriginLab Corporation, Northampton, MA, USA).

### 3.6. Apoptosis Assays

#### 3.6.1. Determination of Apoptotic Cell Death by Annexin V-Cy3 SYTOX Staining Assay

The percentage population of apoptotic cells were determined using Annexin V–Cy3 SYTOX apoptosis detection kit (Abcam, Milton, Cambridge, UK) according to the manufacturer’s instructions. The MCF-7 cells were seeded into six well plates in concentration of 1.5 × 10^6^ cells per well and treated with compound **3b** or **3i** at a concentration of 1 µM against actinomycin D (1 µM) as a reference standard, respectively. The cells were incubated for 48 h and then stained with Annexin V–Cy3 SYTOX green dye. The cells were analyzed using BD Biosciences FACSAria III flow cytometer (BD Biosciences, San Jose, CA, USA). 

#### 3.6.2. Caspase-3 Activation

Caspase-3 activity was detected by means of Caspase-3 Colorimetric Assay Kit (Abcam, Cambridge, MA, USA). The cells were cultured in 24 well plates and treated for 24 h with **3b** or **3i** at a concentration of 1 µM against actinomycin D (1 µM) as a reference standard, respectively. The cells were then washed with PBS buffer and lysed on ice. The experiments were carried out according to the manufacturer’s instructions. Optical density was measured at absorbance of 450 nm using the BioTek microplate reader. The concentration of active Caspase-3 (Asp 175) were measured in duplicates and interpolated from the active caspase-3 (Asp 175) standard curve and corrected for sample dilution.

### 3.7. Tubulin Polymerization Assay

Tubulin polymerization assays were carried out using the tubulin polymerization assay kit (Cytoskeleton, Inc., Denver, CO, USA) following the instruction by the manufacturer. 50 μL of 1.3 mg/mL tubulin (>99% pure) proteins in G-PEM buffer (80 mmol/L PIPES, pH 6.9, 2 mmol/L MgCl_2_, 0.5 mmol/L EGTA, 1 mmol/L GTP, and 15% glycerol) were placed in a quartz cuvette in the presence of the test agent. Polymerization was measured at 37 °C at every three seconds for 1 h using an Applied Photophysics Chirascan spectroflourimeter (Applied Photophysics Ltd., Surrey, UK), excitation at 360 nm, and emission at 450 nm.

### 3.8. EGFR-TK Phosphorylation Inhibition Assays

The inhibitory activities of compounds **3e**, **3i**, **3o**, **3p**, and **3v** against actinomycin D and gefitinib towards EGFR-TK were tested using enzyme-linked immunosorbent assay (ELISA) technique with purified Epidermal Growth Factor Receptor (Sigma-Aldrich, Bradford, UK). The procedure was carried out according to the manufacturer’s protocol and referring to reported instructions as described in our previous investigation [46].

### 3.9. Molecular Docking of Compounds ***3i*** and ***3o*** against Tubulin and EGFR

Molecular docking of compounds **3i** and **3o** to the 3D structure of a tubulin heterodimer (PDB ID: 1SAO) [47] and EGFR (PDB ID: 1M17) [48] as carried out using the CDOCKER protocol [49] in Discovery Studio 2017 (Biovia, San Diego, CA, USA). Prior to performing the docking, compounds were drawn using Discovery Studio and prepared using the ‘Prepare Ligand’ protocol. The protein structures were downloaded from the Protein Data Bank, prepared using the ‘Prepare Protein’ protocol in Discovery Studio, which included removing any existing ligands bound, leaving the water molecules unaltered in the model. The binding sites were defined from receptor cavities and docking was performed using default settings and the best conformation of the ligand were selected and evaluated.

## 4. Conclusions

We have demonstrated that the 5-bromo-2-hydroxy-3-iodochalcones represent important scaffolds for the tandem palladium catalyzed Sonogashira cross-coupling and *endo-dig* cyclization to afford angular benzofuran-chalcone hybrids with the potential to undergo further transformation. The presence of a 2-phenyl ring on the benzofuran moiety and a 4-fluorophenyl (**3b**) or 4-trifluoromethoxyphenyl group (**3e**) on the chalcone arm or a 4-methoxyphenyl on the chalcone and 4-fluorophenyl (**3i**) or 3-chlorophenyl ring (**3s**) on the benzofuran framework increased cytotoxicity against the MCF-7 cell line. The most cytotoxic compounds, **3b** and **3i** were able to trigger apoptosis in the MCF-7 cells, which demonstrates that the benzofuran-chalcones prepared in this investigation possess in vitro antiproliferative and pro-apoptotic activity against this cell line. The observed results from anti-tubulin assays and the corresponding theoretical studies, on the other hand, suggest that the title compounds would significantly interfere with tubulin polymerization by lowering the rate of assembly to elicit anticancer activity. The preliminary results of the EGFR tyrosine kinase assay of the most cytotoxic compound, **3i**, and molecular docking into the ATP binding pocket suggest that this compound may act on the same target enzyme where EGFR-TK inhibitors (e.g., gefitinib) act. Compound **3b**, which exhibited reduced anti-tubulin polymerization effect induced apoptosis against the MCF-7 cells more so than the most cytotoxic derivative **3i** and the reference standard, actinomycin D. This may suggest induction of apoptosis to be one of the main modes of inducing cancer cell death by compound **3b** in addition to inhibiting EGFR-TK phosphorylation. Docking analyses of the binding conformations of the most active compounds in the colchicine-binding site or ATP binding site of EGFR revealed several hydrophobic interactions including hydrogen and/or halogen bonding with the protein residues. These interactions, in our view, led to the observed inhibition of tubulin polymerization or EGFR-TK phosphorylation and therefore significant antiproliferative activity. The modelling studies against the two receptors suggest that halogen bonding with the protein residues help to stabilize the binding of these compounds in the colchicine-binding domain of α,β-tubulin interface and in the binding pocket of EGFR. Since some tyrosine kinase inhibitors target a wide range of kinases [41,50], the results of this investigation provide a rational justification for future evaluation of these compounds for potential inhibitory effects against other types of protein kinases to further explore their mechanism of action and selectivity. Moreover, structural modifications of the benzofuran-appended chalcones **3** are possible to generate derivatives with potential antigrowth effects against various cancer cell lines. Further structural elaboration of the ambident electrophilic C=C–C=O framework, with binucleophilic reagents, for example, would afford novel 5, 6 or 7 membered heterocycle-appended benzofurans with interesting biological properties.

## Figures and Tables

**Table 1:** Compounds **3a**–**y** and their substitution pattern.

R^1^	Substitution Pattern of Compounds 2a–e & 3a–y					
	2	3a–e (R^2^)	3f–j (R^2^)	3k–o (R^2^)	3p–t (R^2^)	3u–y (R^2^)
4-H	**2a**	**3a** (H)	**3f** (4-F)	**3k** (3-F)	**3p** (3-Cl)	**3u** (4-OMe)
4-F	**2b**	**3b** (H)	**3g** (4-F)	**3l** (3-F)	**3q** (3-Cl)	**3v** (4-OMe)
4-Cl	**2c**	**3c** (H)	**3h** (4-F)	**3m** (3-F)	**3r** (3-Cl)	**3w** (4-OMe)
4-OMe	**2d**	**3d** (H)	**3i** (4-F)	**3n** (3-F)	**3s** (3-Cl)	**3x** (4-OMe)
4-OCF_3_	**2e**	**3e** (H)	**3j** (4-F)	**3o** (3-F)	**3t** (3-Cl)	**3y** (4-OMe)

**Table 2:** Cytotoxic effects of **3a**–**y** against MCF-7 cell line. The results are presented as IC_50_ (μM) ± standard deviation (SD) from three individual experiments.

Compounds	MCF-7 Cell Line IC_50_ (μM) ± SD
**3a**	47.96 ± 2.10
**3b**	0.55 ± 0.24
**3c**	>100
**3d**	22.78 ± 0.86
**3e**	0.59 ± 0.21
**3f**	35.81 ± 1.20
**3g**	48.59 ± 2.10
**3h**	54.16 ± 0.40
**3i**	3.55 × 10^−4^ ± 0.07
**3j**	16.00 ± 0.62
**3k**	23.01 ± 0.38
**3l**	>100
**3m**	14.75 ± 0.33
**3n**	39.25 ± 0.42
**3o**	7.87 ± 0.25
**3p**	12.63 ± 0.23
**3q**	23.82 ± 0.29
**3r**	66.75 ± 0.84
**3s**	2.69 ± 0.23
**3t**	26.62 ± 0.27
**3u**	48.96 ± 0.30
**3v**	28.43 ± 0.46
**3w**	30.62 ± 0.35
**3x**	69.09 ± 0.76
**3y**	43.17 ± 0.28
Actinomycin D	37.82 ± 1.30

**Table 3:** Percentages of viable, apoptotic and necrotic cells.

Compound	%Viable Cells (Q3)	%Early Apoptosis (Q4)	%Late Apoptosis (Q2)	%Necrosis (Q1)
**3b**	23.3	1.5	72	3.2
**3i**	32.2	3.1	61.7	3.1
Actinomycin D	30.7	3.4	61.4	4.5

**Table 4:** IC_50_ values of **3b**, **3e**, **3i**, **3j**, **3o**, **3p**, **3s**, and **3v** against tubulin using colchicine as a positive control.

Compounds	IC_50_ (µM)	S.D.
**3b**	26.5	±0.17
**3e**	2.54	±0.24
**3i**	5.51 × 10^−5^	±0.39
**3j**	8.85 × 10^−3^	±0.42
**3o**	1.76 × 10^−4^	±0.11
**3p**	9.37 × 10^−2^	±0.21
**3s**	0.71	±0.30
**3v**	1.62 × 10^−2^	±0.12
Colchicine	9.88 × 10^−2^	±0.17

**Table 5:** IC_50_ values (in µM) of EGFR-TK by **3b 3i**, **3o**, **3p**, and **3v** against actinomycin D and gefitinib.

Compound	EGFR, IC_50_ (μM)
**3b**	0.17 ± 0.03
**3i**	0.09 ± 0.03
**3o**	0.12 ± 0.04
**3p**	0.17 ± 0.06
**3v**	0.15 ± 0.02
Actinomycin D	0.04 ± 0.03
Gefitinib	0.03 ± 0.02

## Supplementary Materials

Supplementary Materials can be found at http://www.mdpi.com/1422-0067/19/9/2552/s1.
