# Supplementary material for: Synthesis, Evaluation for Cytotoxicity and Molecular Docking Studies of Benzo[c]furan-Chalcones for Potential to Inhibit Tubulin Polymerization and/or EGFR-Tyrosine Kinase Phosphorylation
Source: Int J Mol Sci. 2018 Aug 28;19(9):2552. doi: 10.3390/ijms19092552 (PMC6164331; doi:10.3390/ijms19092552)
Supplement: Supplementary file 1 [file ijms-19-02552-s001.zip › ijms-337857-SI.pdf]

Synthesis, evaluation for cytotoxicity and molecular docking studies of benzo[*c*]furan-chalcones for potential to inhibit tubulin polymerization and/or EGFR-tyrosine kinase phosphorylation

Supplementary Materials: ^{1}H and ^{13}C NMR spectra of the prepared compounds **1**, **2a–e** and **3a–y** (Figure S1) and the IC_{50} values for compounds **3a–y** against tubulin polymerization using colchicine as a control (Table S1).

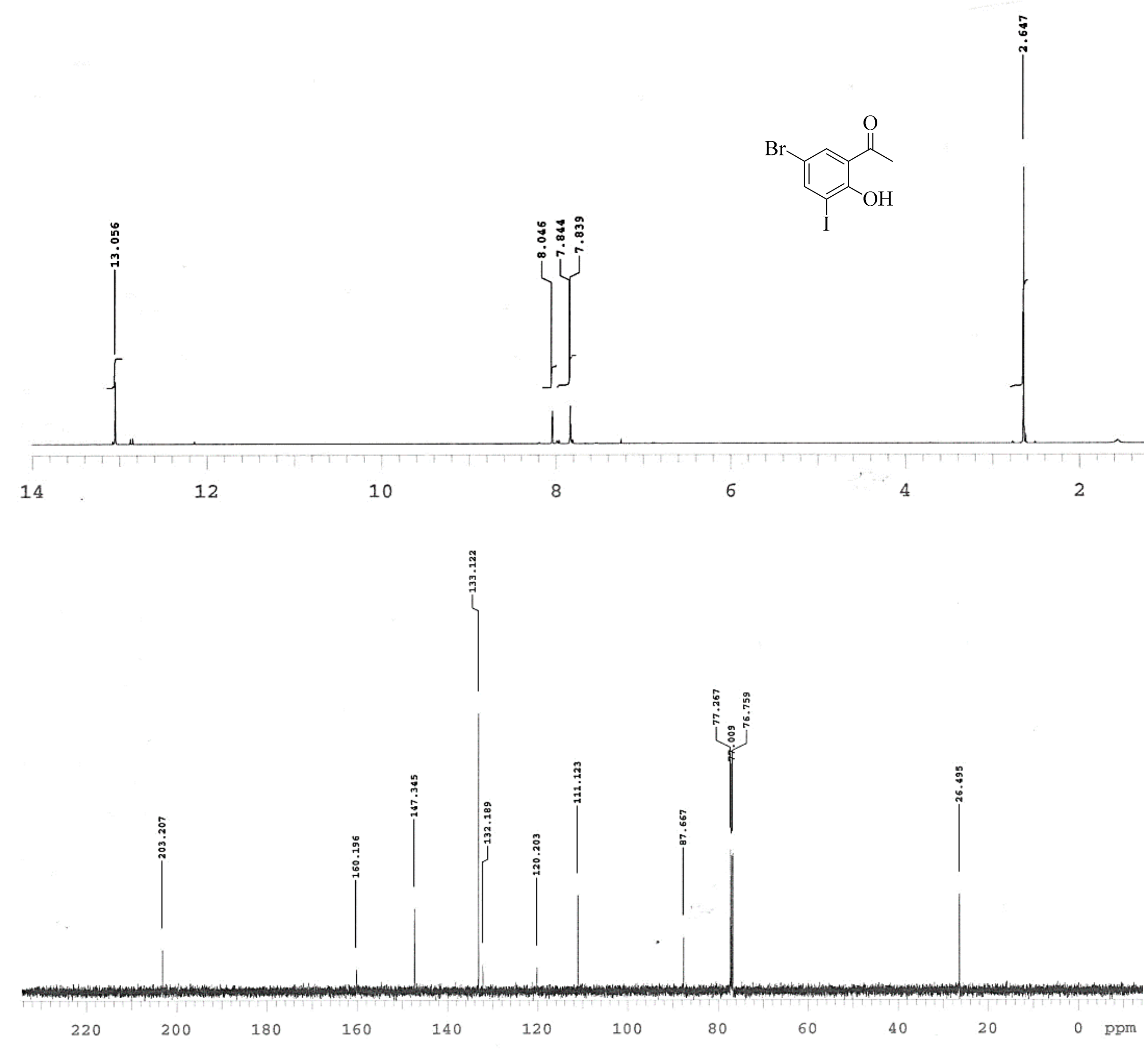

Figure S1.1. ^{1}H- and ^{13}C-NMR spectra of **1** in $CDCl_3$ at 500 and 125 MHz, respectively.

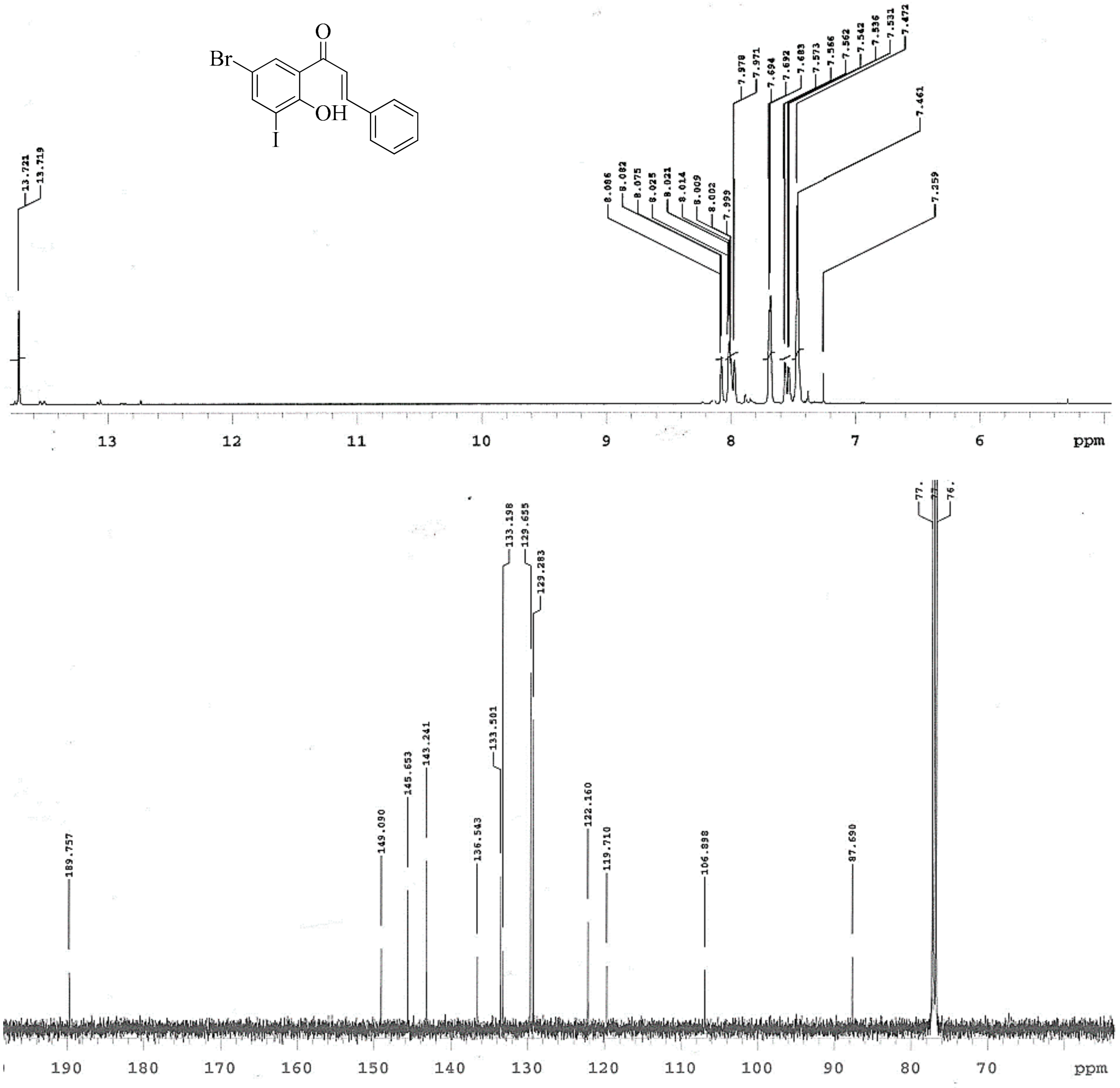

Figure S1.2. ^{1}H- and ^{13}C-NMR spectra of **2a** in $CDCl_3$ at 500 and 125 MHz, respectively.

1-(5-B.romo-2-hydroxy-3-iodophenyl)-3-phenylprop-2-en-1-one (**2a**)

Solid (2.57 g, 72%), mp. 152–154 °C (EtOH); ν_{max} (ATR) 581, 676, 889, 976, 1070, 1173, 1281, 1335, 1581, 1642, 3278 cm^{-1}; δ_H (500 MHz, $CDCl_3$) 7.47 (1H, t, *J* = 7.0, Ar), 7.50 (1H, d, J_{trans} = 15.5 Hz, α-H), 7.68 (2H, d, *J* = 8.0 Hz, Ar), 7.65 (2H, d, *J* = 7.5 Hz, Ar), 8.00 (1H, d, J_{trans} = 15.5 Hz, β-H), 8.01 (1H, d, *J* 2.0 Hz, H-4), 8.07 (1H, d, *J* 2.0 Hz, H-6), 13.5 (1H, br s, OH); δ_C (125 MHz, $CDCl_3$) 110.9, 114.5, 115,9, 120.8, 126.9, 130.1, 130.9, 131.2, 131.9, 142.6, 147.5, 160.3, 190.7; HRMS (ES): found 428.8967. $C_{15}H_{11}O_2{}^{79}BrI^+$ requires 428.8988. *Anal* calcd for $C_{15}H_{10}O_2BrI$: C, 41.99; H, 2.35. Found: C, 41.96; H, 2.32.

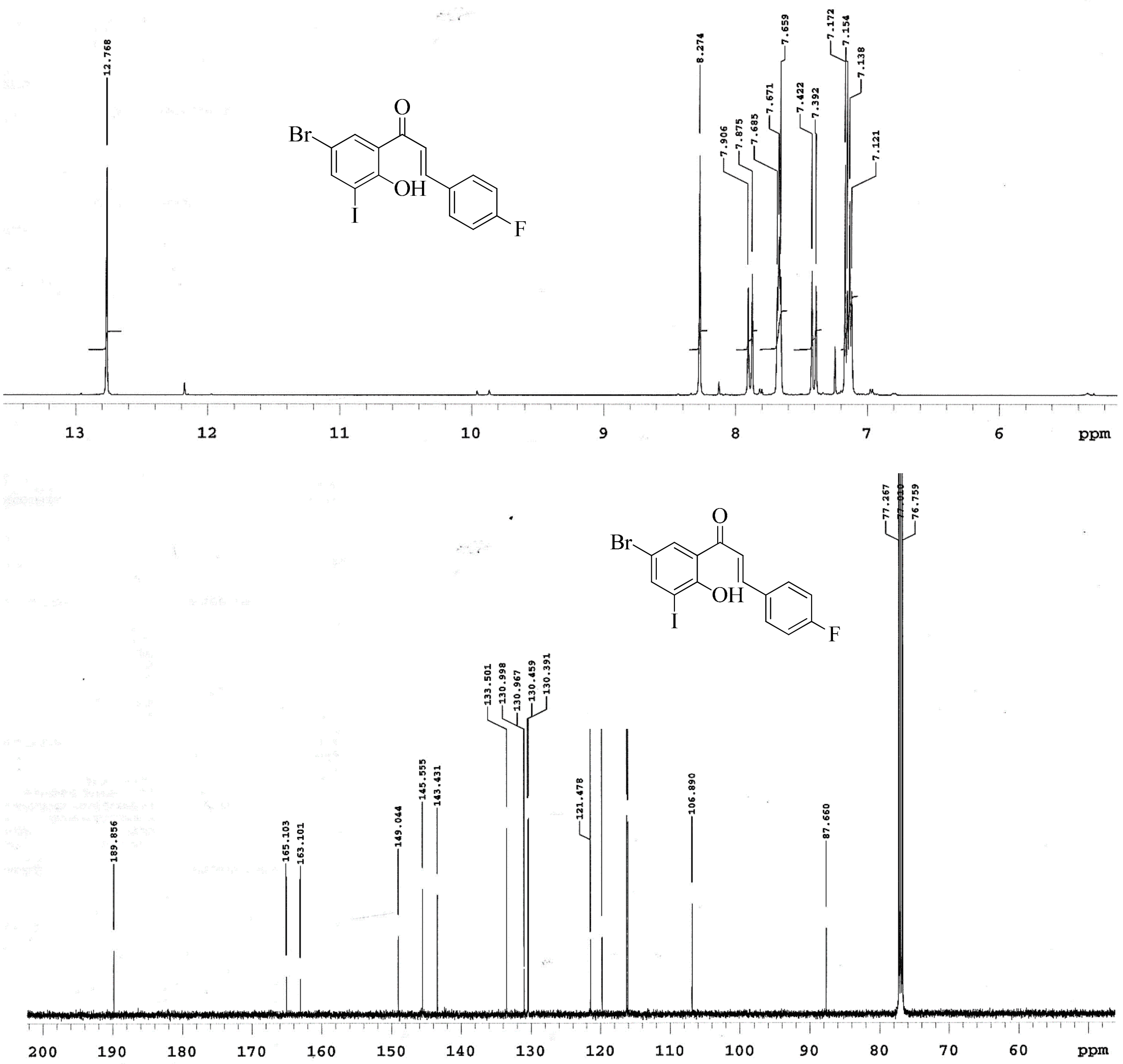

Figure S1.3. ^{1}H- and ^{13}C-NMR spectra of **2b** in $CDCl_3$ at 500 and 125 MHz, respectively.

(*E*)-1-(5-Bromo-2-hydroxy-3-iodophenyl)-3-(4-fluorophenyl)prop-2-en-1-one (**2b**)

Solid (4.11 g, 80%), mp. 177–179 °C (EtOH); ν_{max} (ATR) 542, 674, 825, 975, 1155, 1190, 1504, 1550, 1643, 3202 cm^{-1}; δ_H (500 MHz, $CDCl_3$) 7.15 (2H, t, J = 8.5 Hz) 7.46 (1H, d, J_{trans} = 16.0 Hz, α-H), 7.69 (2H, t, J = 8.0 Hz), 7.95 (1H, d, J_{trans} = 16.0 Hz, β-H), 8.00 (1H, d, J 1.5 Hz. H-4), 8.07 (1H, d, J 2.5 Hz, H-6), 13.7 (1H, br s, OH); δ_C (125 MHz, $CDCl_3$) 87.7, 106.9, 116.1 (d, $^{2}J_{CF}$ = 21.8 Hz), 119.8, 121.4, 130.4 (d, $^{3}J_{CF}$ = 8.5 Hz), 130.9 (d, $^{4}J_{CF}$ = 3.3 Hz), 133.5, 143.4, 145.6, 149.0, 164.1 (d, $^{1}J_{CF}$ = 250.2 Hz), 189.3; HRMS (ES): found 446.8815. $C_{15}H_{10}O_2F^{79}BrI^+$ requires 446.9053. *Anal* calcd for $C_{15}H_9O_2FBrI$: C, 40.30; H, 2.03. Found: C, 40.19; H, 1.98.

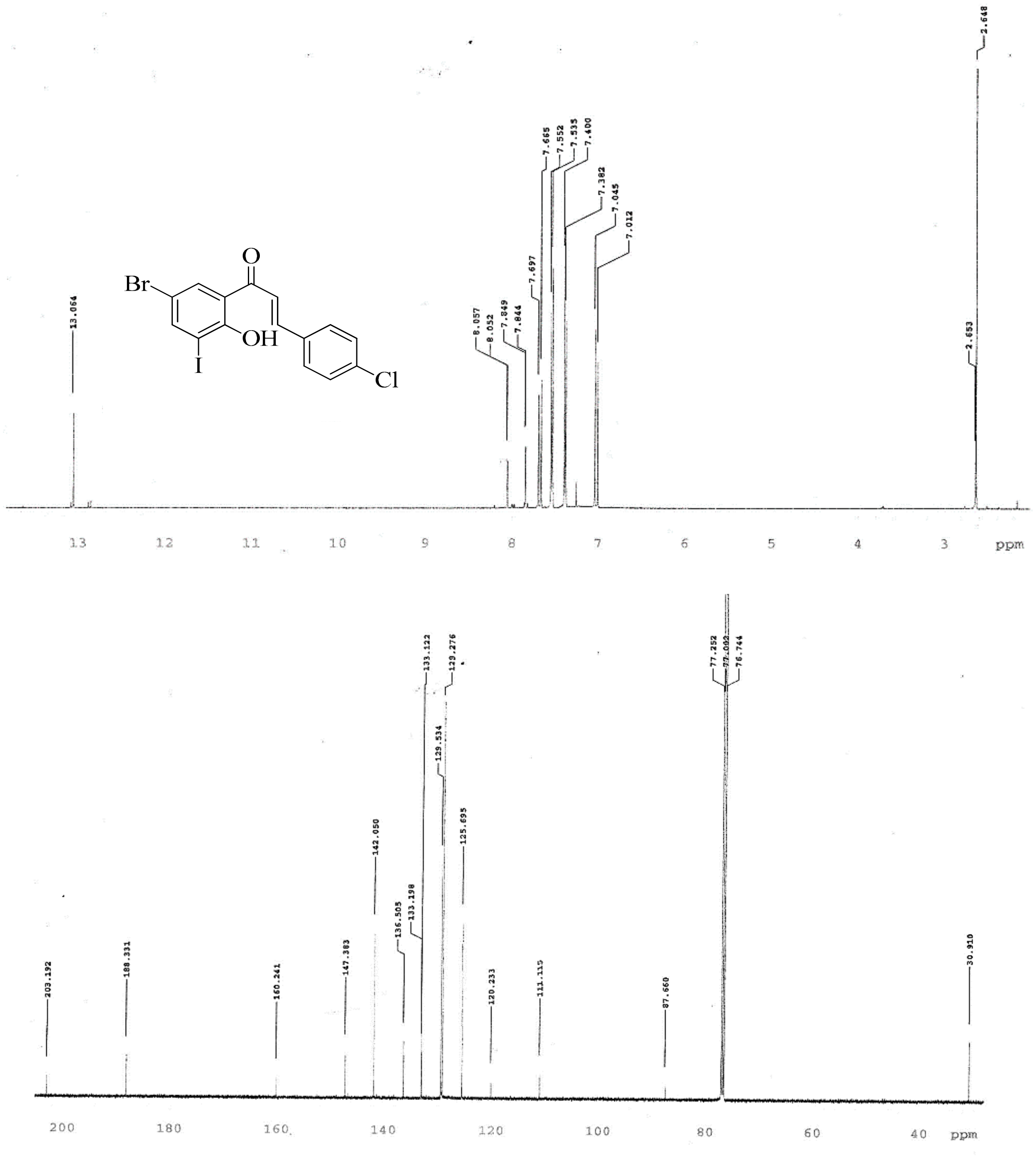

Figure S1.4. ^{1}H- and ^{13}C-NMR spectra of **2c** in $CDCl_3$ at 500 and 125 MHz, respectively.

1-(5-B.romo-2-hydroxy-3-iodophenyl)-3-(4-chlorophenyl)prop-2-en-1-one (**2c**)

Solid (4.11 g, 80%), mp. 184–186 °C (EtOH); ν_{max} (ATR) 675, 821, 1013, 1091, 1189, 1488, 1506, 154, 1584, 1641, 3249 cm^{-1}; δ_H (500 MHz, $CDCl_3$) 1.58 (1H, br s, OH) 7.03 (1H, d, J_{trans} = 16.0 Hz, α-H), 7.39 (2H, d, J = 8.5 Hz, H-2',6'), 7.55 (2H, d, J = 8.5 Hz, H-3',5'), 7.67 (1H, d, J_{trans} = 16.0 Hz, β-H), 7.84 (1H, d, J 2.5 Hz. H-4), 8.05 (1H, d, J 2.5 Hz, H-6); δ_C (125 MHz, $CDCl_3$) 87.7, 111.1, 120.2, 125.7, 129.3, 129.5, 133.1, 133.2, 136.5, 142.1, 147.4, 160.2, 188.3 (C=O); HRMS (ES): found 462.8519. $C_{15}H_{10}O_2{}^{35}Cl^{79}BrI^+$ requires 462.8598. *Anal* calcd for $C_{15}H_9O_2ClBrI$: C, 38.87; H, 1.96. Found: C, 38.77; H, 2.04.

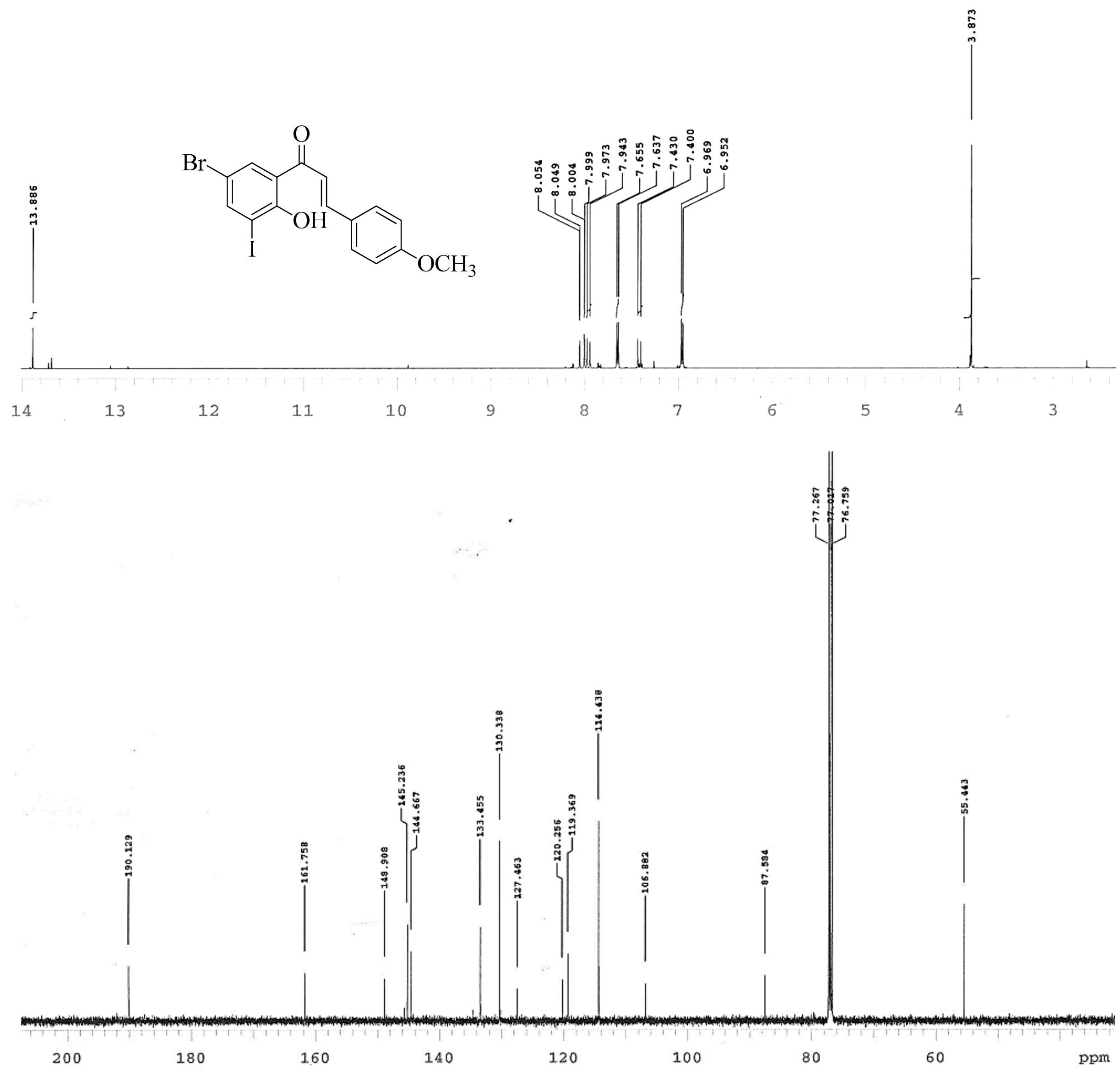

Figure S1.5. ^{1}H- and ^{13}C-NMR spectra of **2d** in $CDCl_3$ at 500 and 125 MHz, respectively.

1-(5-B.romo-2-hydroxy-3-iodophenyl)-3-(4-methoxyphenyl)prop-2-en-1-one (**2d**)

A mixture of **1** (4.08 g, 11.96 mmol), 4-methoxybenzaldehyde (1.95 g, 14.35 mmol) and KOH (3.34 g, 59.80 mmol) in methanol (100 mL) afforded **2d** as an orange solid (3.83 g, 69%), mp. 173–175 °C (EtOH); ν_{max} (ATR) 537, 673, 824, 985, 1166, 1259, 1506, 1548, 1634, 3276 cm^{-1}; δ_H (500 MHz, $CDCl_3$) 3.88 (3H, s, OCH_3) 6.96 (2H, d, *J* = 8.5 Hz, H-3',5'), 7.42 (1H, d, J_{trans} = 16.0 Hz, α-H), 7.65 (2H, d, *J* = 8.5 Hz, H-2',6'), 7.96 (1H, d, J_{trans} = 16.0 Hz, β-H), 8.01 (1H, d, *J* 2.0 Hz, H-4), 8.05 (1H, d, *J* 2.0 Hz, H-6), 13.5 (1H, br s, OH); δ_C (125 MHz, $CDCl_3$) 55.6, 114.3, 115.9, 126.8, 129.9, 130.0, 130.9, 131.0, 131.9, 140.9, 147.5, 162.5, 164.6, 190.7; HRMS (ES): MH^+, found 458.9095. $C_{16}H_{13}O_3{}^{79}BrI^+$ requires 458.9093. *Anal* calcd for $C_{16}H_{12}O_3BrI$: C, 41.86; H, 2.63. Found: C, 41.93; H, 2.75.

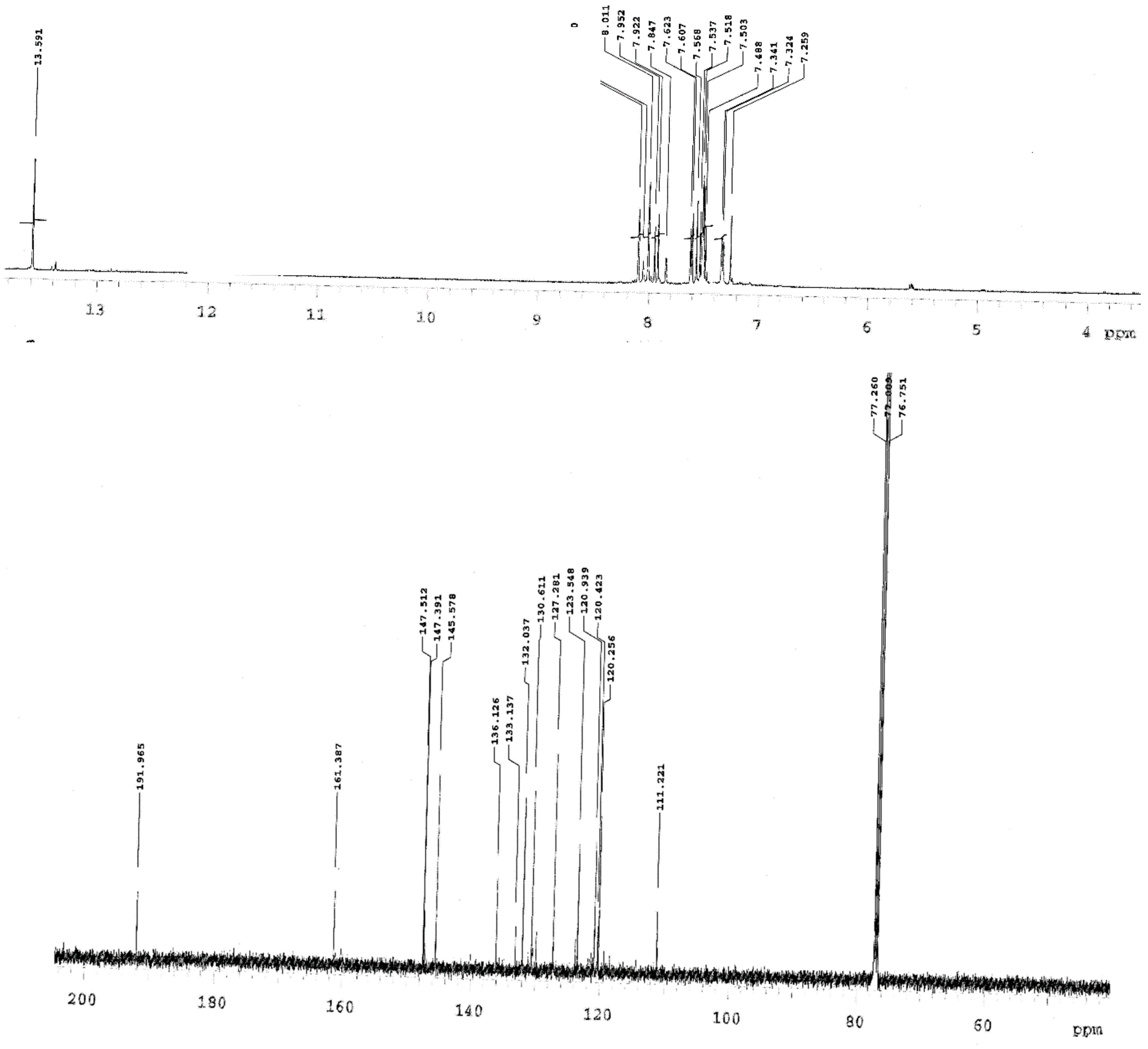

Figure S1.6. ^{1}H- and ^{13}C-NMR spectra of **2e** in $CDCl_3$ at 500 and 125 MHz, respectively.

1-(5-B.romo-2-hydroxy-3-iodophenyl)-3-(4-(trifluoromethoxy)phenyl)prop-2-en-1-one (**2e**)

A mixture of **1** (2.98 g, 8.79 mmol), 4-trifluoromethoxybenzaldehyde (2.15 g, 10.54 mmol) and KOH (2.46 g, 43.95 mmol) in methanol (100 mL) afforded **2c** as an orange solid (3.33 g, 74%), mp. 106–107 ºC; ν_{max} (ATR) 542, 691, 869, 1033, 1140, 1259, 1343, 1447, 1573, 1642, 3283 cm^{-1}; δ_H (500 MHz, $CDCl_3$) 7.33 (1H, d, *J* = 8.0 Hz, H-3'), 7.50 (2H, d, *J* = 8.5 Hz, H-2',6'), 7.54 (1H, d, J_{trans} = 16.0 Hz, α-H), 7.61 (1H, d, *J* = 8.0 Hz, H-5'), 7.94 (1H, d, J_{trans} = 16.0 Hz, β-H), 8.01 (1H, d, *J* 2.0 Hz, H-4), 8.10 (1H, d, *J* 2.0 Hz, H-6), 13.6 (1H, br s, OH); δ_C (125 MHz, $CDCl_3$) 112, 120.2 (t, J_{CF} = 256.0 Hz), 123.8, 127.2, 129.9, 130.6, 132.0, 133.1, 136.1, 145.5, 147.3, 147.5, 161.4, 191.9; HRMS (ES): MH$^+$, found 512.8633 $C_{16}H_{10}O_3{}^{79}BrIF_3{}^+$ requires 512.8811. *Anal* calcd for $C_{16}H_9O_3BrIF_3$: C, 37.46; H, 1.77. Found: C, 371.53; H, 1.74.

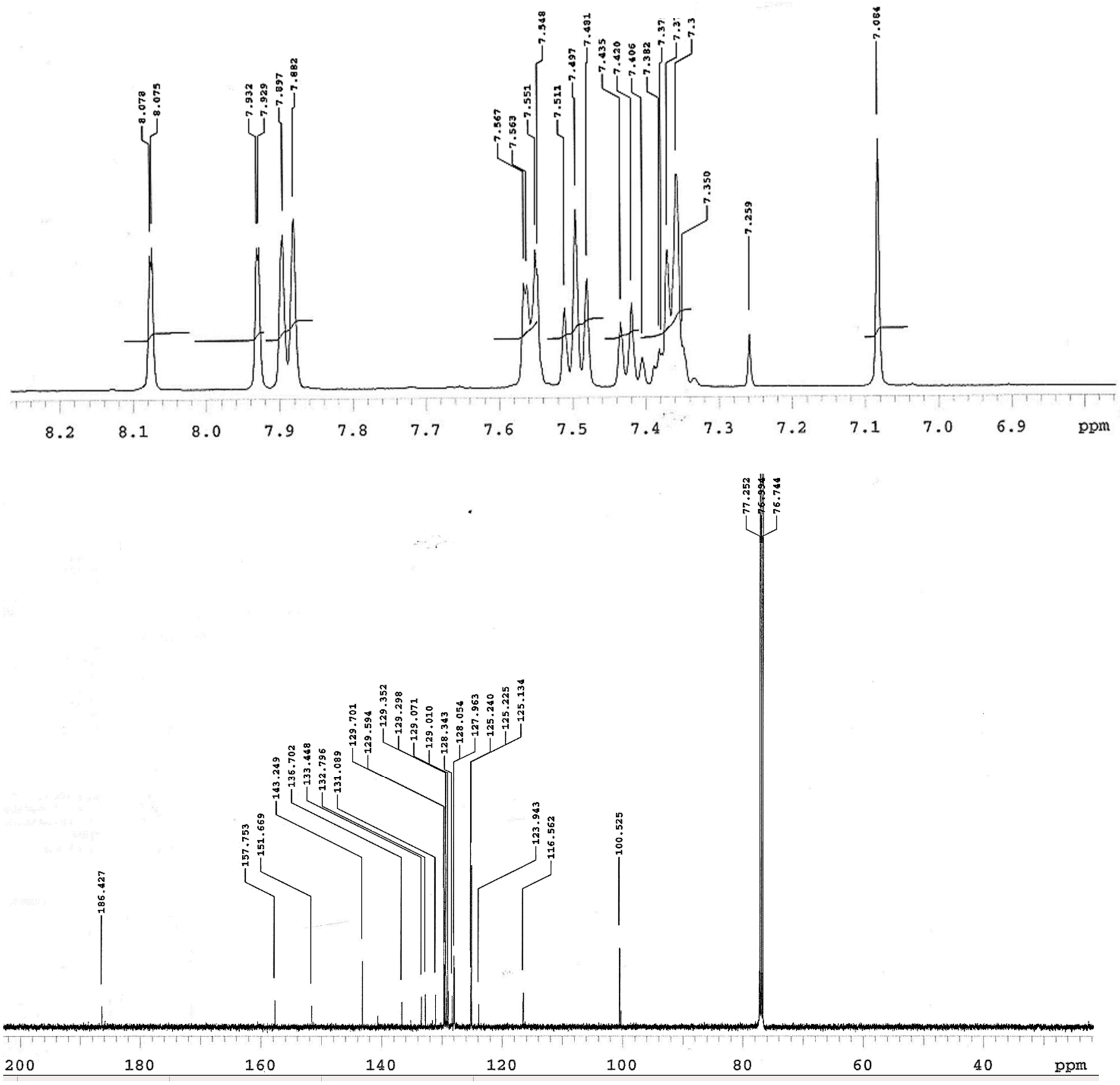

Figure S1.7. ^{1}H- and ^{13}C-NMR spectra of **3a** in $CDCl_3$ at 500 and 125 MHz, respectively.

(*E*)-1-(5-Bromo-2-phenylbenzofuran-7-yl)-3-phenylprop-2-en-1-one (**3a**)

Solid (0.63 g, 66%); mp. 197–195 °C; ν_{max} (ATR) 567, 680, 758, 853, 972, 1097, 1176, 1283, 1365, 1491, 1574, 1596, 1656 cm^{-1}; δ_H (500 MHz, $CDCl_3$) 7.06 (1H, s, =CH), 7.15 (1H, t, *J* = 7.5 Hz), 7.42 (2H, d, *J* =7.0 Hz), 7.54–7.49 (3H, m, Ar), 7.56 (2H, d, *J* = 8.0 Hz, Ar), 7.65 (1H, *J* = 2.5 Hz, H-4), 7.68 (2H, d, *J* = 8.0 Hz, Ar), 7.74 (1H, d, J_{trans} = 16.0 Hz, α-H), 7.80 (1H, d, J_{trans} = 16.0 Hz, β-H), 7.95 (1H, d, *J* 2.5 Hz, H-6); δ_C (125 MHz, $CDCl_3$) 88.6, 88.9, 100.9, 118.5, 122.0, 123.1, 125.1, 128.3, 128.4, 128.6, 128.8, 129.0, 129.3, 129.4, 131.1, 131.6, 152.8, 157.8, 195.2; HRMS (ES): found 403.0325. $C_{23}H_{16}O_2{}^{79}Br^+$ requires 403.0334. *Anal* calcd for $C_{23}H_{15}O_2Br$: C, 68.50; H, 3.75. Found: C, 68.38; H, 3.73.

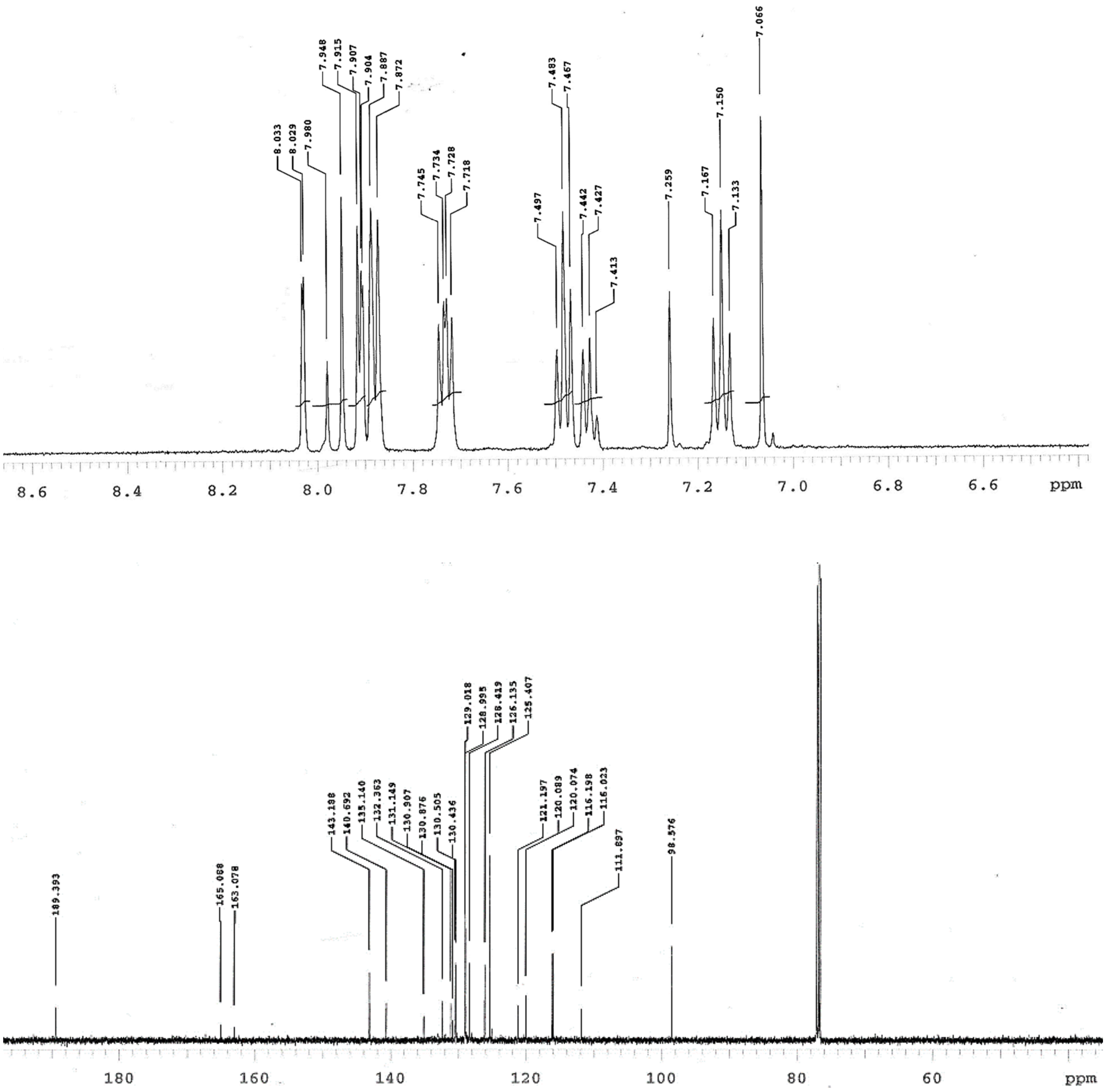

Figure S1.8. ^{1}H- and ^{13}C-NMR spectra of **3b** in $CDCl_3$ at 500 and 125 MHz, respectively.

(*E*)-1-(5-Bromo-2-phenylbenzofuran-7-yl)-3-(4-fluorophenyl)prop-2-en-1-one (**3b**)

Solid (0.73 g, 79%); mp. 220–223 °C; ν_{max} (ATR) 579, 690, 798, 835, 997, 1098, 1155, 1278, 1363, 1433, 1502, 1585, 1673 cm^{-1}; δ_H (300 MHz, $CDCl_3$) 7.07 (1H, s, =CH), 7.15 (2H, t, *J* = 8.5 Hz, Ar), 7.43 (2H, d, *J* = 8.5 Hz, Ar), 7.48 (2H, t, *J* = 8.0 Hz, Ar), 7.73 (1H, t, *J* = 8.0 Hz, Ar), 7.88 (2H, d, *J* = 7.5 Hz, Ar), 7.91 (1H, d *J* = 1.5 Hz, H-4), 7.93 (1H, d, J_{trans} = 16.0 Hz, α-H), 7.95 (1H, d, J_{trans} = 16.0 Hz, β-H), 8.03 (1H, d, *J* = 2.4 Hz, H-6); δ_C (75 MHz, $CDCl_3$) 100.5, 116.3 (d, $^2J_{CF}$ = 21.8 Hz), 116.5, 124.1, 124.6, 125.1, 127.8, 128.1, 129.1, 129.3, 129.5, 130.5 (d, $^3J_{CF}$ = 8.5 Hz), 131.2 (d, $^4J_{CF}$ = 2.9 Hz), 132.8, 143.5, 151.6, 157.7, 164.2 (d, $^1J_{CF}$ = 239.0 Hz), 186.5; HRMS (ES): found 421.0254. $C_{23}H_{15}O_2F^{79}Br^+$ requires 421.0239. *Anal* calcd for $C_{23}H_{14}O_2FBr$: C, 65.58; H, 3.35. Found: C, 65.42; H, 3.41.

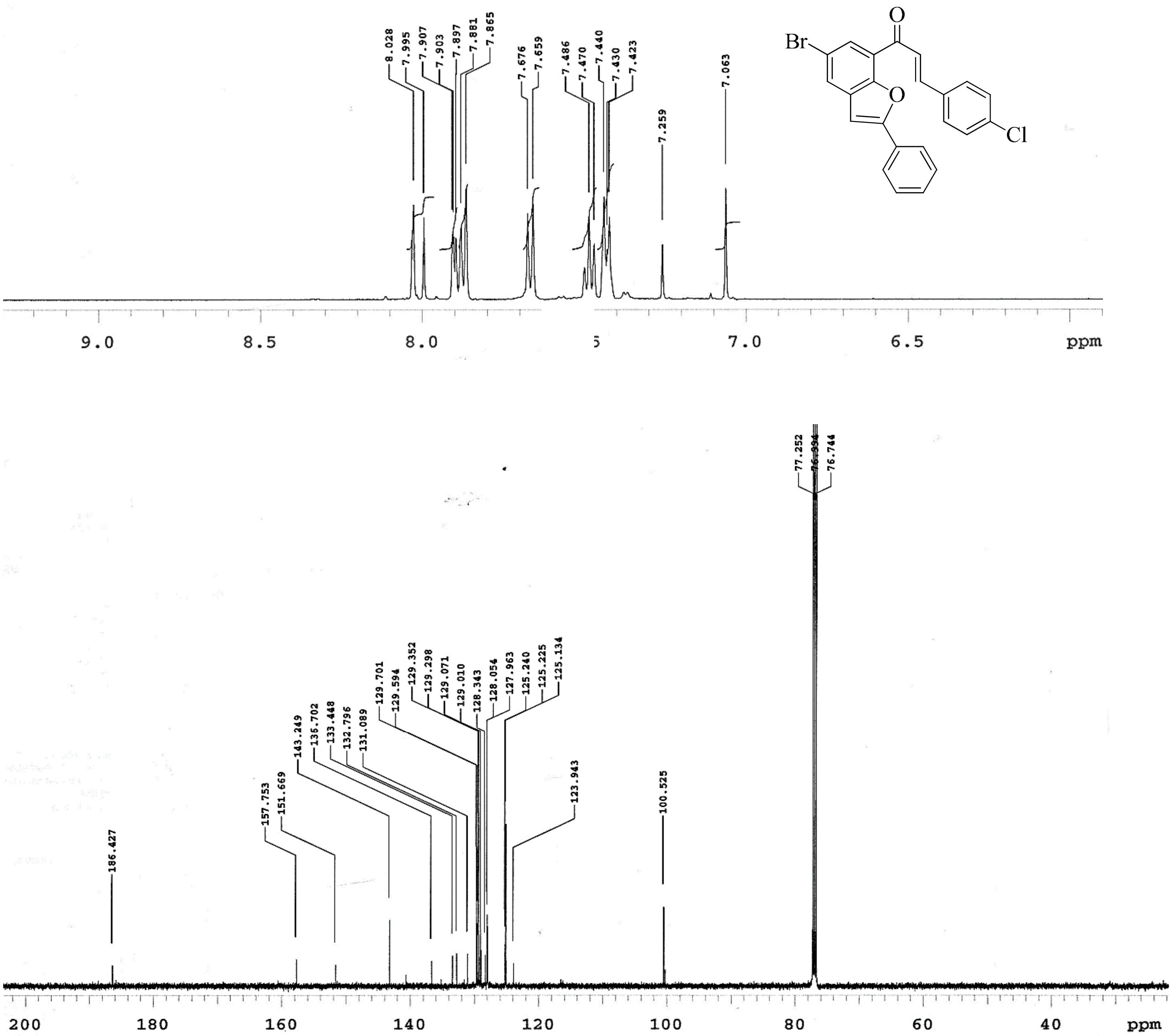

Figure S1.9. ^1H- and ^{13}C-NMR spectra of **3c** in $CDCl_3$ at 500 and 125 MHz, respectively.

(*E*)-1-(5-Bromo-2-phenylbenzofuran-7-yl)-3-(4-chlorophenyl)prop-2-en-1-one (**3c**)

Solid (0.79, 78%); mp. 242–245 °C; ν_{max} (ATR) 524, 685, 751, 814, 974, 1090, 1217, 1317, 1365, 1489, 1588, 1592, 1659 cm^{-1}; δ_H (500 MHz, $CDCl_3$) 7.06 (1H, s, =CH), 7.42 (2H, d, *J* = 8.5 Hz, Ar), 7.49 (1H, t, *J* = 8.5 Hz, Ar), 7.67 (2H, d, *J* = 8.7 Hz, Ar), 7.84 (2H, d, *J* = 8.0 Hz, Ar), 7.86 (1H, d, J_{trans} = 16.0 Hz, α-H), 7.88 (1H, d, *J* = 1.5 Hz, H-4), 7.91 (1H, d, *J* = 2.5 Hz, 6-H), 8.02 (1H, d, J_{trans} = 16.0 Hz, β-H), 8.04 (2H, d, *J* = 7.0 Hz, Ar); δ_C (125 MHz, $CDCl_3$) 116.5, 125.1, 125.2, 125.3, 127.9, 128.1, 128.3, 129.0, 129.3, 129.6, 129.7, 131.1, 132.8, 133.4, 136.7, 143.2, 151.6, 157.7, 186.4; HRMS (ES): found 436.9937. $C_{23}H_{15}O_2{}^{35}Cl^{79}Br^+$ requires 436.9945. *Anal* calcd for $C_{23}H_{14}O_2ClBr$: C, 63.11; H, 3.22. Found: C, 63.08; H, 3.17.

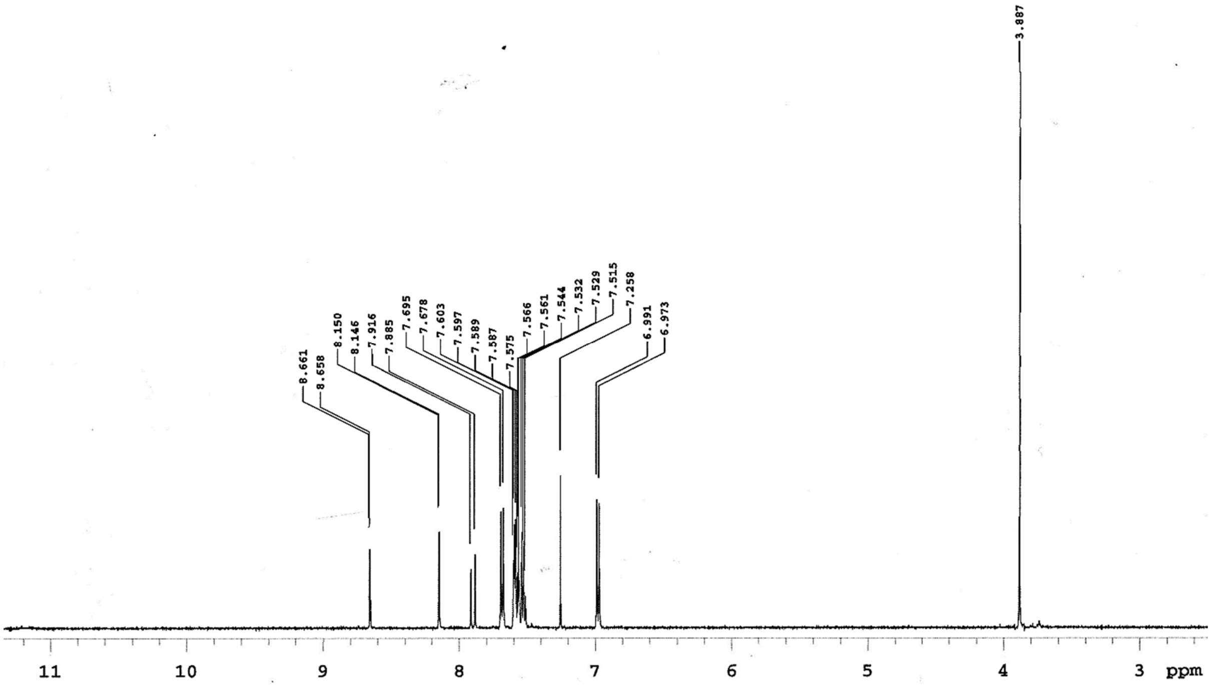

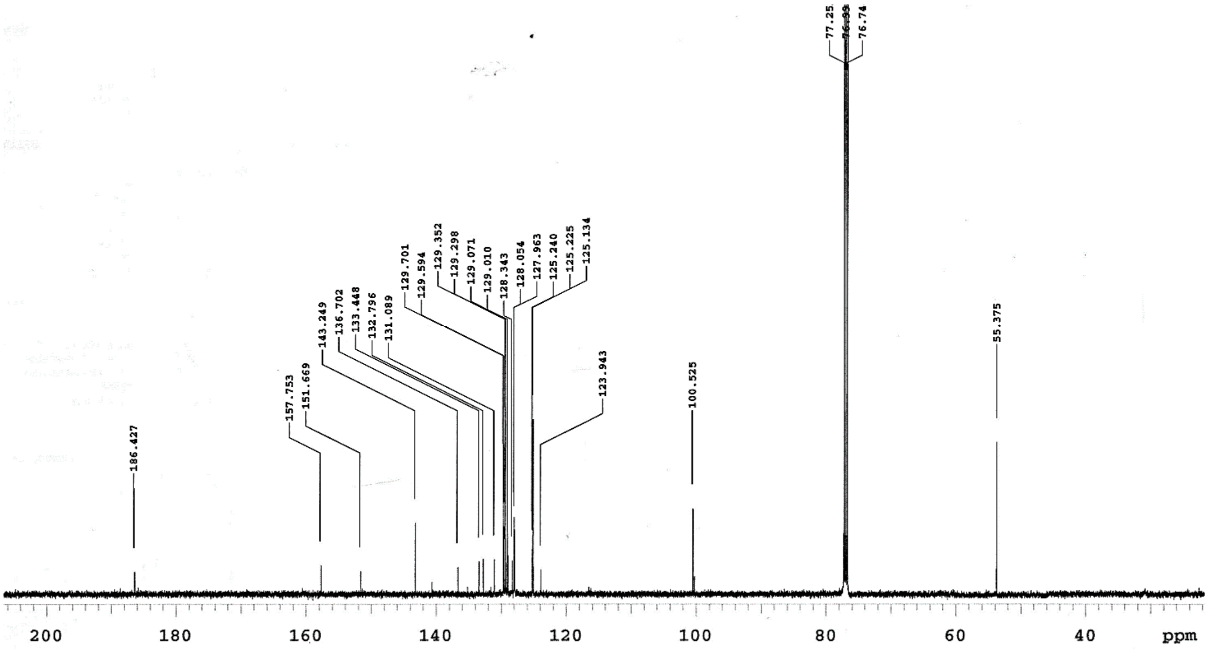

Figure S1.10: ^{1}H- and ^{13}C-NMR spectra of **3d** in $CDCl_3$ at 500 and 125 MHz, respectively.

(*E*)-1-(5-Bromo-2-phenylbenzofuran-7-yl)-3-(4-methoxyphenyl)prop-2-en-1-one (**3d**)

Solid (0.65 g, 72%); mp. 206–208 °C; ν_{max} (ATR) 531, 672, 766, 870, 991, 1023, 1170, 1256, 1365, 1422, 1509, 1558, 1654 cm^{-1}; δ_H (500 MHz, $CDCl_3$) 3.86 (3H, s, OCH_3), 6.98 (2H, d, *J* = 8.5 Hz, Ar), 7.14 (1H, s, =CH), 7.19 (1H, t, *J* = 8.0 Hz, Ar), 7.44 (2H, d, *J* = 8.7 Hz, Ar), 7.81 (2H, d, *J* = 8.0 Hz, Ar), 7.83 (1H, d, J_{trans} = 16.0 Hz, α-H), 7.88 (1H, d, J_{trans} = 16.0 Hz, β-H), 7.92 (2H, d, *J* = 8.5 Hz, Ar), 8.00 (1H, d, *J* = 2.5 Hz, 4-H), 8.02 (1H, d, *J* = 2.5 Hz, 6-H); δ_C (125 MHz, $CDCl_3$) 55.4, 83.5, 97.1, 105.7, 112.0, 114.4, 119.6, 119.9, 122.4, 127.6, 128.5, 128.8, 129.4, 130.2, 131.6, 133.4, 138.8, 144.2, 161.6, 186.7; HRMS MH$^+$, found 433.0364. $C_{24}H_{18}O_3{}^{79}Br^+$ requires 433.0361. *Anal* calcd for $C_{24}H_{17}O_3Br$: C, 66.53; H, 3.95. Found: C, 66.59; H, 3.93.

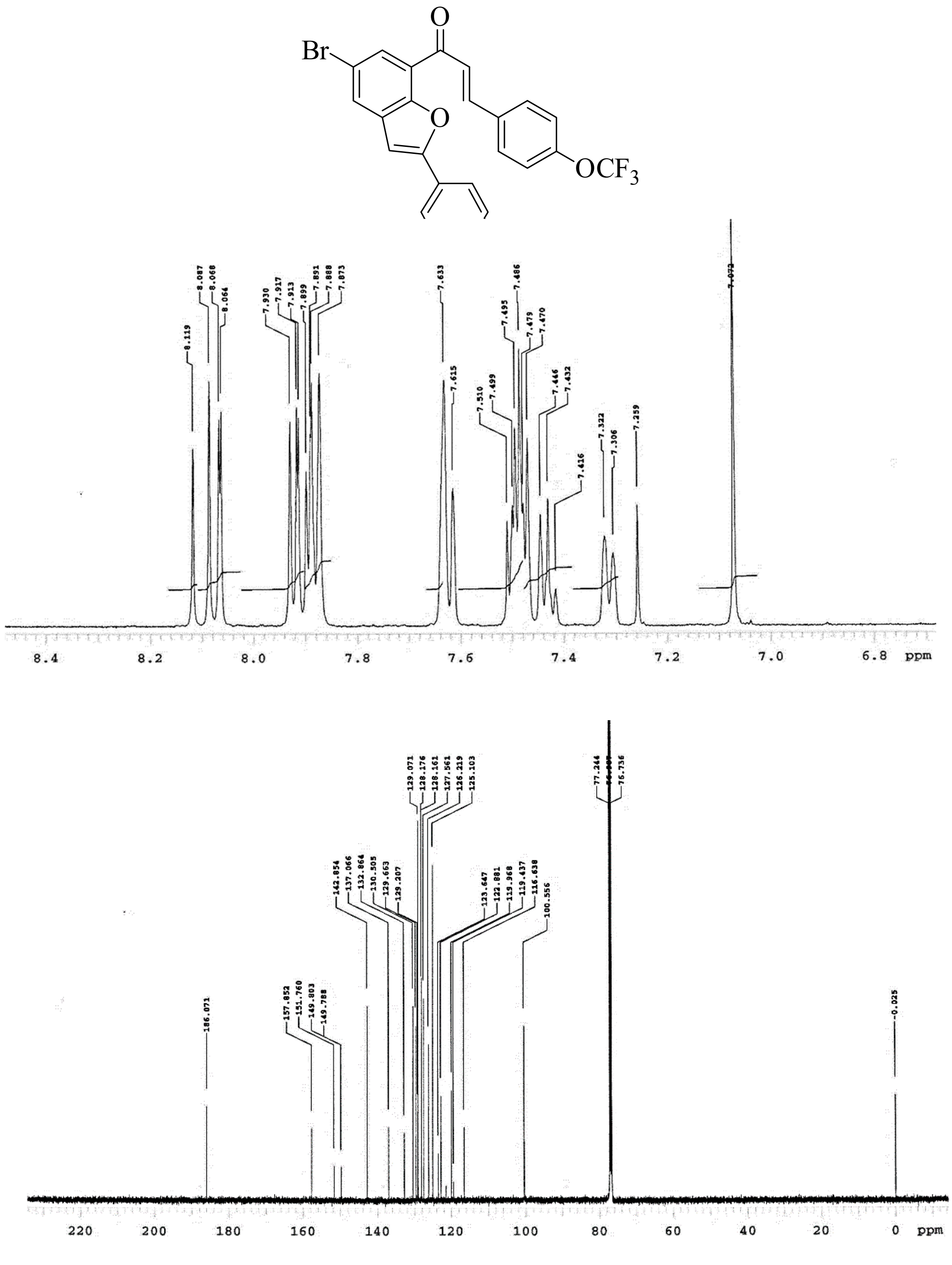

Figure S1.11: ^{1}H- and ^{13}C-NMR spectra of **3e** in $CDCl_3$ at 500 and 125 MHz, respectively.

(*E*)-1-(5-Bromo-2-phenylbenzofuran-7-yl)-3-(4-(trifluoromethoxy)phenyl)prop-2-en-1-one (**3e**)

Solid (0.33 g, 67%); 177–178 °C; ν_{max} (ATR) 568, 685, 790, 866, 969, 1036, 1147, 1253, 1409, 1492, 1600, 1658 cm^{-1}; δ_H (500 MHz, $CDCl_3$) 7.07 (1H, s, =CH), 7.31 (1H, d, *J* = 8.0 Hz, Ar), 7.43 (1H, t, *J* =8.0 Hz, Ar), 7.47 (2H, d, *J* = 8.0 Hz, Ar), 7.49 (2H, *J* = 7.5 Hz, Ar), 7.62 (2H, d, *J* = 8.7 Hz, Ar), 7.88 (1H, d, *J* = 8.7 Hz, Ar), 7.91 (1H, d, J_{trans} = 16.0 Hz, α-H), 7.92 (1H, d, *J* 2.0 Hz, H-4), 8.06 (1H, d, *J* 2.0 Hz, H-6), 8.09 (1H, d, J_{trans} = 16.0 Hz, β-H); δ_C (125 MHz, $CDCl_3$) 100.5, 116.6, 119.9 (t, J_{CF} = 256.0 Hz), 122.8, 123.6, 125.1, 126.2, 129.6, 128.1, 129.0, 129.2, 129.6, 130.5, 132.9, 137.1, 142.8, 149.7, 151.8, 157.9, 186.1; HRMS (ES): found 487.0148. $C_{24}H_{15}O_3{}^{79}BrF_3{}^{+}$ requires 487.0157. *Anal* calcd for $C_{24}H_{14}O_3BrF_3$: C, 59.16; H, 2.90. Found: C, 59.13; H, 3.01.

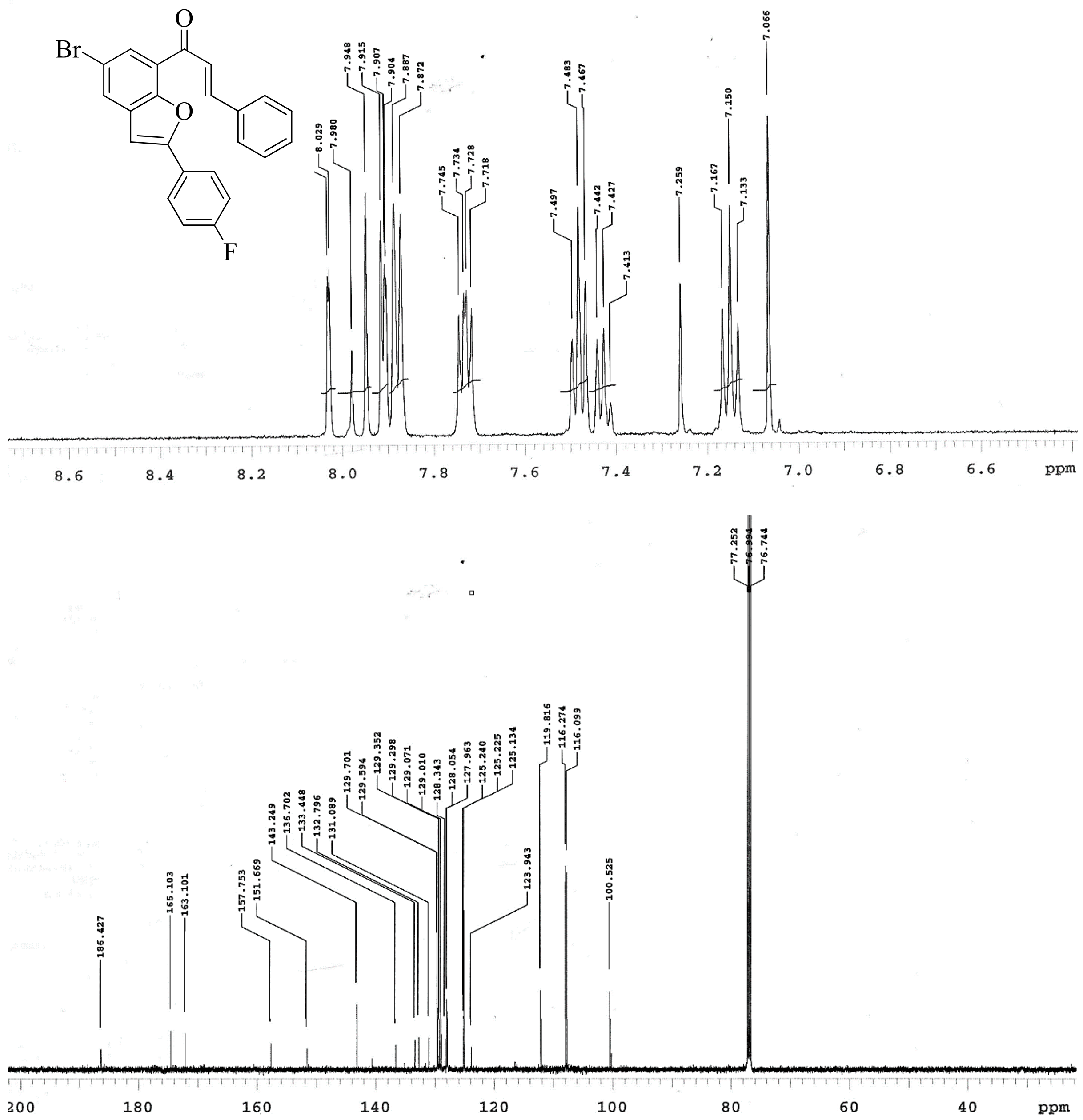

Figure S1.12: ^{1}H- and ^{13}C-NMR spectra of **3f** in $CDCl_3$ at 500 and 125 MHz, respectively.

(*E*)-1-(5-Bromo-2-(4-fluorophenyl)benzofuran-7-yl)-3-phenylprop-2-en-1-one (**3f**)

Solid (0.69 g, 70%); mp. 224–226 °C; ν_{max} (ATR) 575, 682, 751, 826, 976, 1099, 1172, 1217, 1366, 1432, 1508, 1587, 1656 cm^{-1}; δ_H (500 MHz, $CDCl_3$) 6.97 (1H, s, =CH), 7.18 (1H, t, J = 8.5 Hz, Ar), 7.44–7.46 (4H, m, Ar), 7.51 (2H, t, J = 8.0 Hz, Ar), 7.72 (1H, d, J_{trans} = 16.0 Hz, α-H), 7.74 (1H, d, J_{trans} = 16.0 Hz, β-H), 7.84 (2H, t, J = 8.5 Hz, Ar), 7.86 (1H, d, J = 2.0 Hz, H-4), 7.87 (1H, d, J = 2.0 Hz, H-6); δ_C (125 MHz, $CDCl_3$) 100.5, 116.2 (d, $^{2}J_{CF}$ = 21.9 Hz), 116.5, 124.0, 124.5, 125.1, 127.9, 128.1, 129.0, 129.3, 129.5, 130.4 (d, $^{3}J_{CF}$ = 8.5 Hz), 131.2, 132.4 (d, $^{4}J_{CF}$ = 3.8 Hz), 143.5, 151.5, 157.8, 161.2 (d, $^{1}J_{CF}$ = 245.0 Hz), 186.5; HRMS (ES): found: 421.0161. $C_{23}H_{15}FO_2{}^{79}Br^+$ requires 421.0141. *Anal* calcd for $C_{23}H_{14}FO_2Br$: C, 65.58; H, 3.35. Found: C, 68.49; H, 3.33.

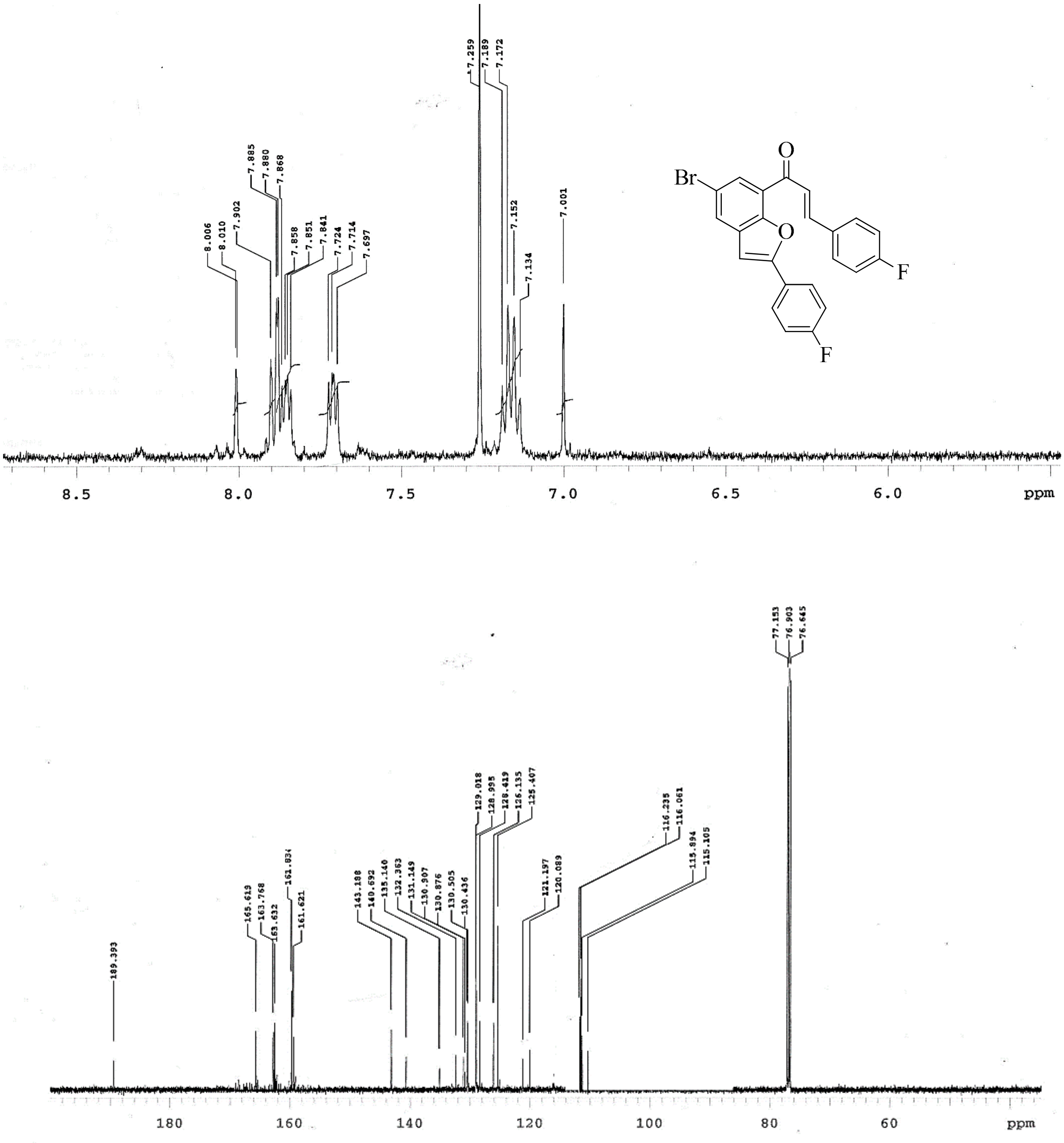

Figure S1.13: ^{1}H- and ^{13}C-NMR spectra of **3g** in $CDCl_3$ at 500 and 125 MHz, respectively.

(*E*)-1-(5-Bromo-2-(4-fluorophenyl)benzofuran-7-yl)-3-(4-fluorophenyl)prop-2-en-1-one (**3g**)

Solid (0.88 g, 76%); mp. 239–242 °C; ν_{max} (ATR) 575, 645, 755, 860, 977, 1096, 1157, 1285, 1365, 1414, 1598, 1658, 1678 cm^{-1}; δ_H (500 MHz, $CDCl_3$) 6.99 (1H, s, =CH), 7.17 (2H, t, *J* = 8.5 Hz, Ar), 7.46 (2H, t, *J* = 8.5 Hz, Ar), 7.73 (2H, t, *J* = 8.7 Hz, Ar), 7.86 (2H, t, *J* = 8.5 Hz, Ar), 7.89 (1H, d, *J* = 2.0 Hz, H-4), 7.94 (1H, d, J_{trans} = 16.0 Hz, α-H), 8.00 (1H, d, J_{trans} = 16.0 Hz, β-H), 8.02 (1H, d, *J* = 2.5 Hz, H-6); δ_C (125 MHz, $CDCl_3$) 100.5, 115.1 (d, $^{2}J_{CF}$ = 21.9 Hz), 116.2 (d, $^{2}J_{CF}$ = 21.9 Hz), 124.1, 124.6, 125.1, 127.9, 128.1, 129.0, 129.6 (d, $^{4}J_{CF}$ = 3.8 Hz), 130.4 (d, $^{3}J_{CF}$ = 8.5 Hz), 132.0 (d, $^{3}J_{CF}$ = 8.5 Hz), 132.7 (d, $^{4}J_{CF}$ = 3.8 Hz), 143.4, 151.6, 157.3, 162.1 (d, $^{1}J_{CF}$ = 248.0 Hz), 162.9 (d, $^{1}J_{CF}$ = 267.3 Hz), 186.5; HRMS (ES): found 439.0134. $C_{23}H_{14}O_2F_2{}^{79}Br^+$ requires 439.0146. *Anal* calcd for $C_{23}H_{13}O_2F_2Br$: C, 62.89; H, 2.98. Found: C, 62.87; H, 2.93.

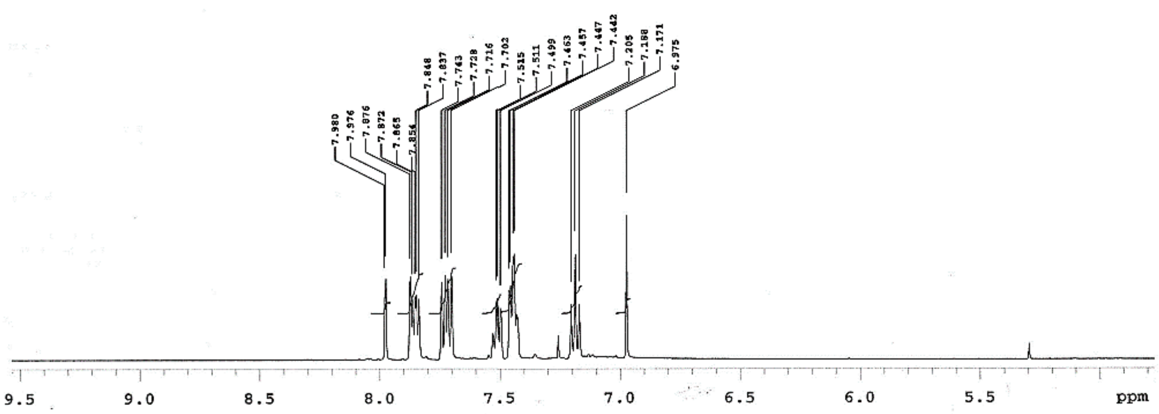

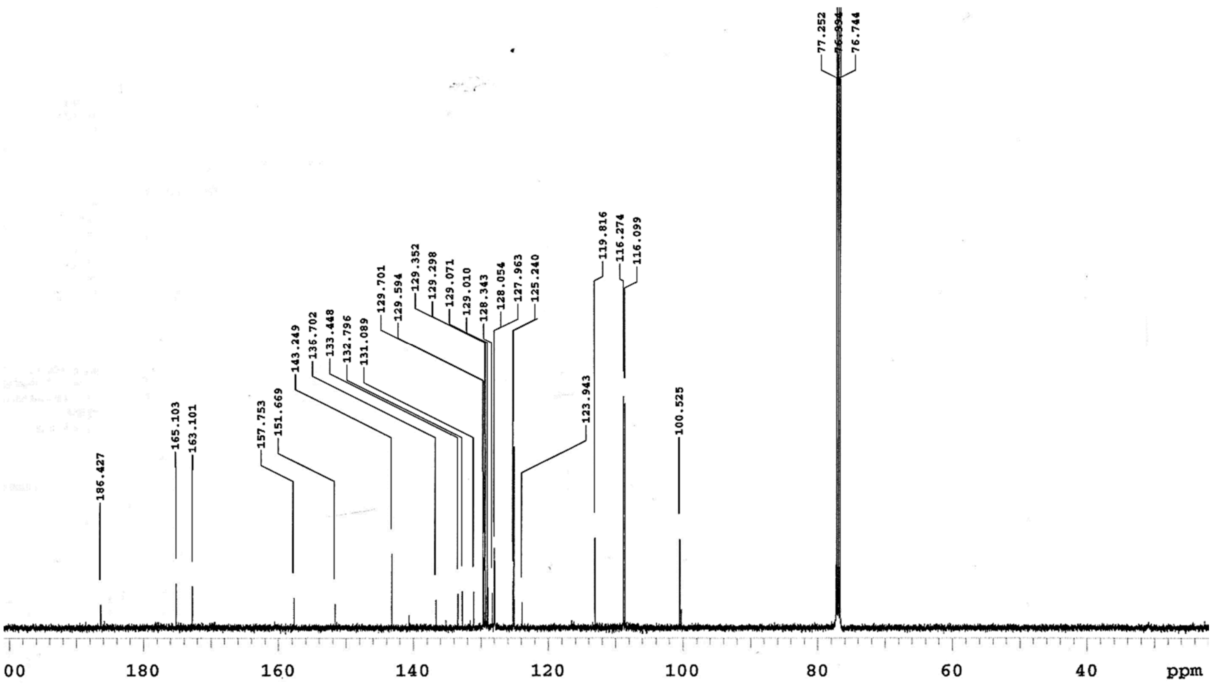

Figure S1.14: ^{1}H- and ^{13}C-NMR spectra of **3h** in $CDCl_3$ at 500 and 125 MHz, respectively.

(*E*)-1-(5-Bromo-2-(4-fluorophenyl)benzofuran-7-yl)-3-(4-chlorophenyl)prop-2-en-1-one (**3h**)

Solid (0.69 g, 77%); mp. 231–233 °C; ν_{max} (ATR) 579, 666, 741, 891, 987, 1097, 1145, 1248, 1337, 1447, 1502, 1598, 1674 cm^{-1}; δ_H (500 MHz, $CDCl_3$) 7.06 (1H, s, =CH), 7.15 (2H, t, *J* = 8.0 Hz, Ar), 7.46 (2H, d, *J* = 8.0 Hz, Ar), 7.72 (2H, t, *J* = 8.5 Hz, Ar), 7.87 (2H, d, *J* = 7.5 Hz, Ar), 7.91 (1H, d, *J* = 2.0 Hz, H-4), 7.93 (1H, d, J_{trans} = 16.0 Hz, α-H), 7.98 (1H, d, J_{trans} = 16.0 Hz, β-H), 8.03 (1H, d, *J* = 2.0 Hz, H-6); δ_C (125 MHz, $CDCl_3$) 100.5, 116.3 (d, $^{2}J_{CF}$ = 21.9 Hz), 120.2, 124.1, 124.3, 124.4, 125.6, 129.3, 129.5, 130.4 (d, $^{3}J_{CF}$ = 8.7 Hz), 131.2, 132.4 (d, $^{4}J_{CF}$ = 3.8 Hz), 142,1, 143.6, 147.3, 151.5, 156.8, 161.3 (d, $^{1}J_{CF}$ = 245.0 Hz), 188.4; HRMS (ES): found 454.9873. $C_{23}H_{14}O_2F^{35}Cl^{79}Br^+$ requires 454.9850. *Anal* calcd for $C_{23}H_{13}O_2F^{35}Cl^{79}Br$: C, 60.62; H, 2.88. Found: C, 60.68; H, 2.85.

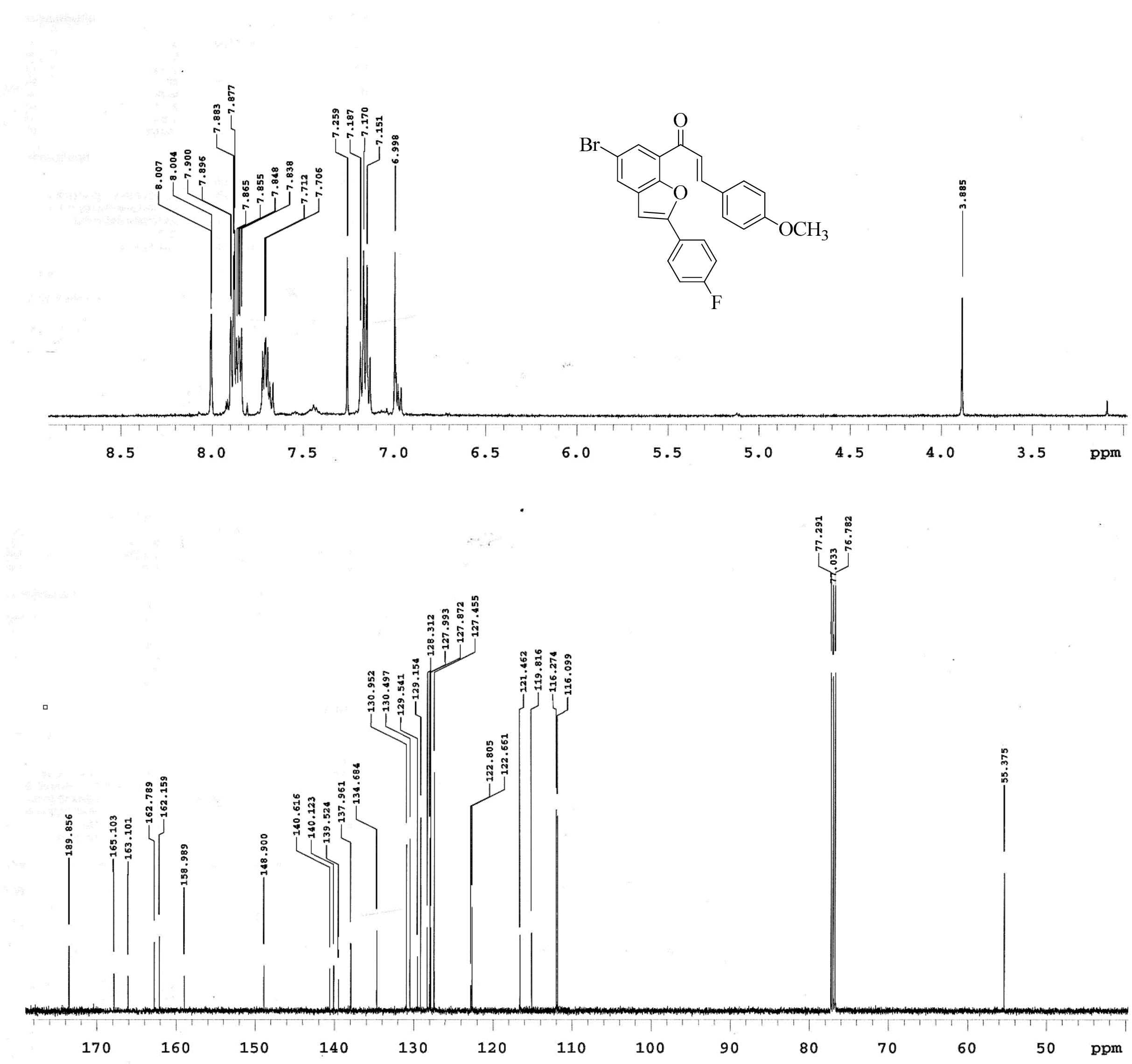

Figure S1.15: ^{1}H- and ^{13}C-NMR spectra of **3i** in $CDCl_3$ at 500 and 125 MHz, respectively.

(*E*)-1-(5-Bromo-2-(4-fluorophenyl)benzofuran-7-yl)-3-(4-methoxyphenyl)prop-2-en-1-one (**3i**)

Solid (0.65 g, 72%); mp. 252–254 °C; ν_{max} (ATR) 526, 654, 791, 878, 976, 1099, 1159, 1287, 1364, 1455, 1572, 1589, 1655 cm^{-1}; δ_H (500 MHz, $CDCl_3$) 3.86 (3H, s, OCH_3), 6.98 (2H, d, J = 8.5 Hz, Ar), 7.14 (1H, s, =CH), 7.19 (1H, t, J = 8.0 Hz, Ar), 7.44 (2H, d, J = 8.7 Hz, Ar), 7.81 (1H, d, J = 8.0 Hz, Ar), 7.83 (1H, d, J_{trans} = 16.0 Hz, α-H), 7.88 (1H, d, J_{trans} = 16.0 Hz, β-H), 7.92 (2H, d, J = 8.5 Hz, Ar), 8.00 (1H, d, J = 2.5 Hz, H-4), 8.02 (1H, d, J = 2.5 Hz, H-6); δ_C (125 MHz, $CDCl_3$) 55.5, 100.2, 114.6, 116.3 (d, $^{2}J_{CF}$ = 21.9 Hz), 124.1, 124.3, 124.4, 125.8, 127.0, 127.6, 128.8, 130.4 (d, $^{3}J_{CF}$ = 8.7 Hz), 131.2, 132.4 (d, $^{4}J_{CF}$ = 3.8 Hz), 143.6, 144.9, 151.5, 156.8, 161.6 (d, $^{1}J_{CF}$ = 245.0 Hz), 186.8; HRMS (ES): found 451.0327. $C_{24}H_{17}O_3{}^{79}Br^+$ requires 451.0345. *Anal* calcd for $C_{24}H_{16}O_3Br$: C, 63.87; H, 3.57. Found: C, 68.83; H, 3.62.

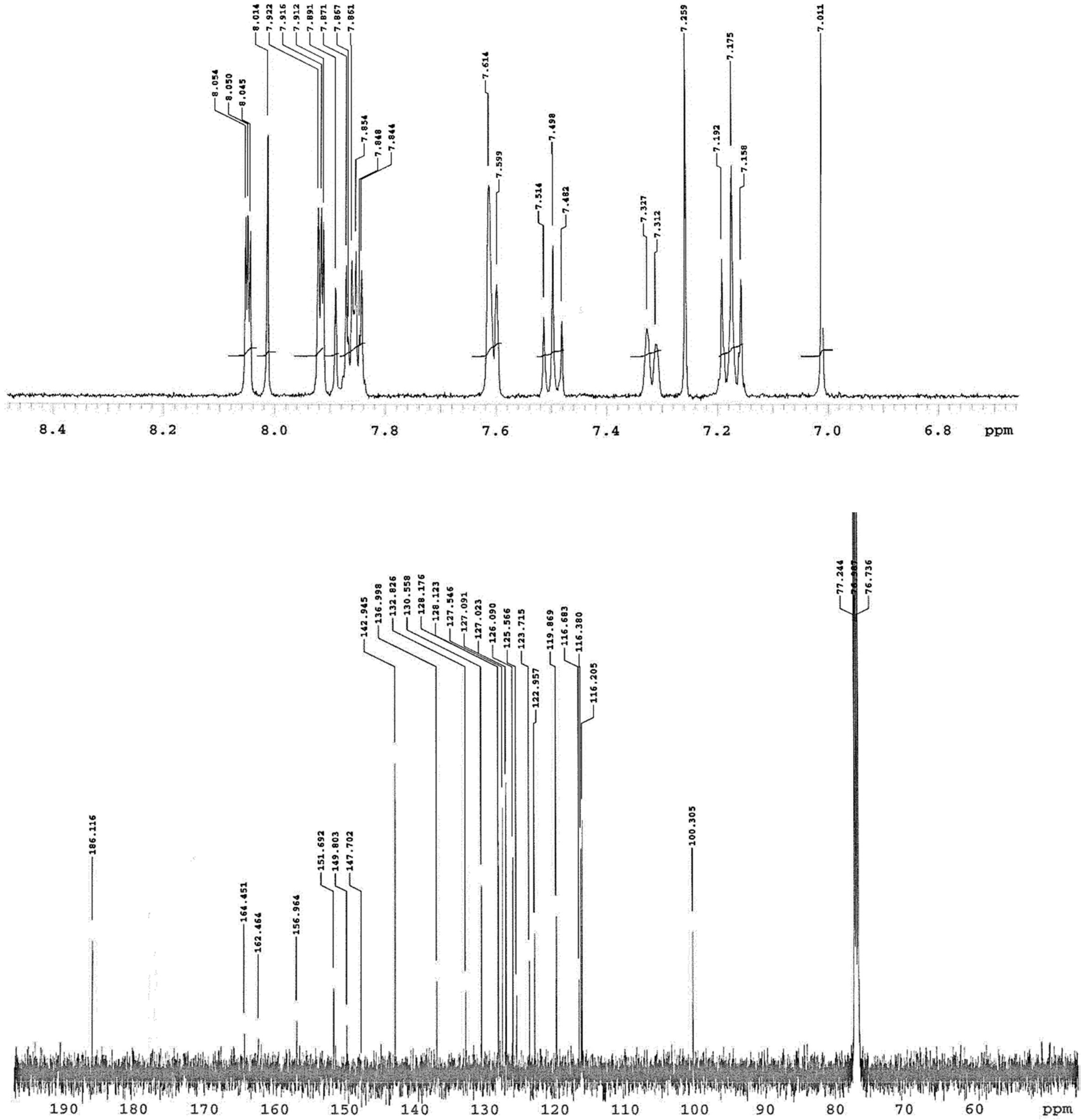

Figure S.16: ^{1}H- and ^{13}C-NMR spectra of **3j** in $CDCl_3$ at 500 and 125 MHz, respectively.

(*E*)-1-(5-Bromo-2-(4-fluorophenyl)benzofuran-7-yl)-3-(4-(trifluoromethoxy)phenyl)prop-2-en-1-one (**3j**)

Solid (0.48 g, 64%); mp. 203–204 °C; ν_{max} (ATR) 580, 695, 789, 892, 978, 1094, 1169, 1262, 1345, 1577, 1601, 1660 cm^{-1}; 7.01 (1H, s, =CH), 7.17 (1H, t, *J* = 8.5 Hz, Ar), 7.31 (2H, d, *J* = 7.5 Hz, Ar), 7.49 (1H, t, *J* = 8.0 Hz, Ar) 7.60 (2H, d, *J* = 7.5 Hz, Ar), 7.96 (2H, t, *J* = 8.5 Hz, Ar), 7.90 (1H, d, J_{trans} = 16.0 Hz, α-H), 7.92 (1H, d, *J* = 2.0 Hz, H-4), 8.00 (1H, d, J_{trans} = 16.0 Hz, β-H), 8.05 (1H, d, *J* = 2.0 Hz, H-6); δ_C (125 MHz, $CDCl_3$) 100.3, 116.3 (d, $^{2}J_{CF}$ = 21.9 Hz), 116.7, 119.8 (t, J_{CF} = 256.0 Hz), 123.7, 124.3, 125.5, 126.1, 127.0 (d, $^{3}J_{CF}$ = 8.7 Hz), 127.5, 128.1 (d, $^{4}J_{CF}$ = 3.8 Hz), 130.5, 132.8, 136.9, 142.9, 149.8, 151.6, 156.9, 163.4 (d, $^{1}J_{CF}$ = 245.0 Hz), 186.1; HRMS (ES): found 504.0044. $C_{24}H_{14}O_3{}^{79}BrF_4{}^{+}$ requires 504.0066. *Anal* calcd for $C_{24}H_{13}O_3BrF_4$: C, 57.05; H, 2.59. Found: C, 57.08; H, 2.64.

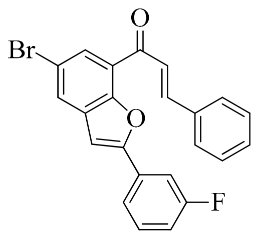

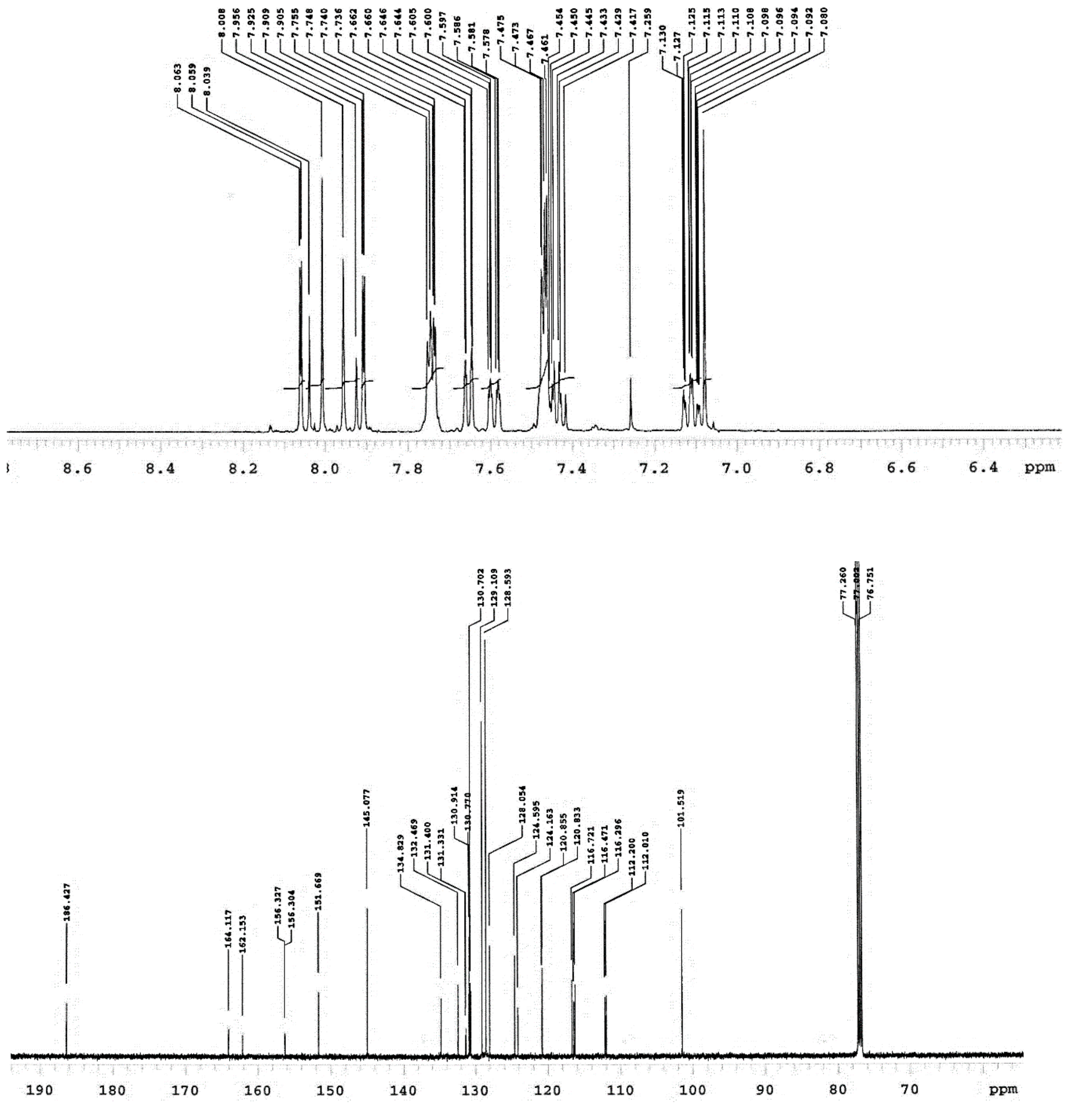

Figure S1.17: ^{1}H- and ^{13}C-NMR spectra of **3k** in $CDCl_3$ at 500 and 125 MHz, respectively.

(*E*)-1-(5-Bromo-2-(3-fluorophenyl)benzofuran-7-yl)-3-phenylprop-2-en-1-one (**3k**)

Solid (0.73 g, 70%); mp. 243–245 °C; ν_{max} (ATR) 503, 664, 765, 854, 939, 1039, 1159, 1220, 1325, 1448, 1588, 1655 cm^{-1}; δ_H (500 MHz, $CDCl_3$) 7.08 (1H, s, =CH), 7.11 (1H, t, *J* = 8.0 Hz, Ar), 7.43 (1H, t, *J* = 8.0 Hz, Ar), 7.46 (1H, d, *J* = 8.0 Hz, Ar), 7.47 (1H, d, *J* = 8.0 Hz, Ar), 7.59 (1H, dt, *J* = 8.0 and 2.0 Hz, Ar), 7.65 (1H, d, *J* = 8.0 Hz, Ar), 7.73-7.75 (3H, m, Ar), 7.91 (1H, d, *J* = 2.0 Hz, H-4), 7.93 (1H, d, J_{trans} =

16.0 Hz, α-H), 8.02 (1H, d, J_{trans} = 16.0 Hz, β-H), 8.06 (1H, d, J = 2.0 Hz, H-6); δ_C (125 MHz, $CDCl_3$) 101.5, 112.2 (d, $^3J_{CF}$ = 23.7 Hz), 116.3 (d, $^2J_{CF}$ = 21.9 Hz), 116.7, 120.8, 120.9, 124.2, 124.6, 128.1, 128.6, 129.1, 130.7, 130.8 (d, $^6J_{CF}$ = 1.8 Hz), 131.4 (d, $^4J_{CF}$ = 8.6 Hz), 132.5, 134.8, 145.1, 151.6, 156.3 (d, $^5J_{CF}$ = 2.8 Hz), 163.1 (d, $^1J_{CF}$ = 245.0 Hz), 186.4; HRMS (ES): found: 421.0243. $C_{23}H_{15}O_2{}^{79}Br\ F^+$ requires 421.0239. *Anal* calcd for $C_{23}H_{14}O_2BrF$: C, 65.52; H, 3.35. Found: C, 65.80; H, 3.41.

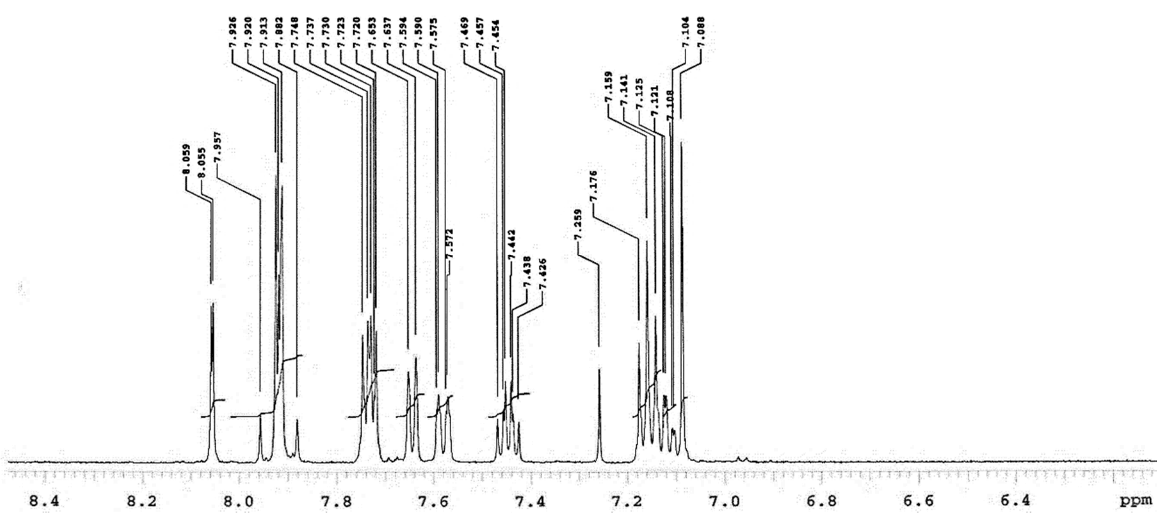

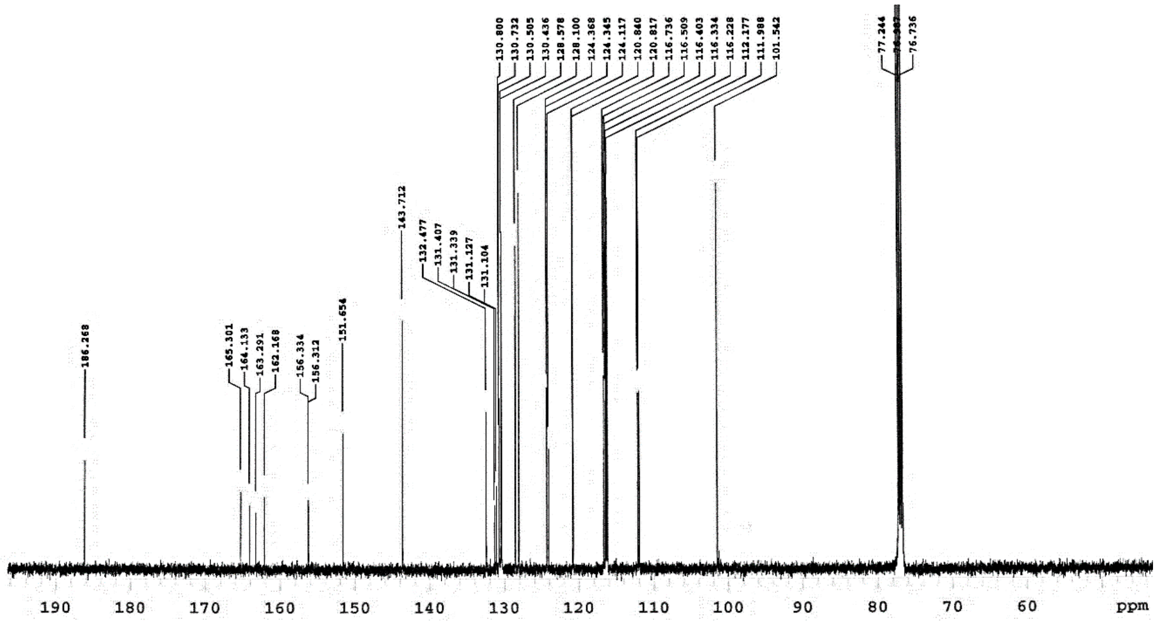

Figure S1.18: ^{1}H- and ^{13}C-NMR spectra of **3l** in $CDCl_3$ at 500 and 125 MHz, respectively.

(*E*)-1-(5-Bromo-2-(3-fluorophenyl)benzofuran-7-yl)-3-(4-fluorophenyl)prop-2-en-1-one (**3l**)

Solid (0.67 g, 77%); mp. 266–267 °C; ν_{max} (ATR) 572, 680, 779, 891, 976, 1171, 1281, 1346, 1488, 1597, 1660 cm^{-1}; δ_H (500 MHz, $CDCl_3$) 7.08 (1H, s, =CH), 7.12 (1H, t, J = 8.5 Hz, Ar), 7.42 (1H, d, J = 8.0 Hz, Ar), 7.45 (2H, d, J = 7.5 Hz, Ar), 7.59 (1H, dt, J = 8.5 and 2.0 Hz, Ar), 7.64 (1H, d, J = 8.0 Hz, Ar), 7.73 (2H, t, J = 8.5 Hz, Ar), 7.90 (1H, d, J_{trans} = 16.0 Hz, α-H), 7.92 (1H, d, J = 2.5 Hz, H-4), 7.95 (1H, d, J_{trans} = 16.0 Hz, β-H), 8.05 (1H, d, J = 2.5 Hz, H-6); δ_C (125 MHz, $CDCl_3$) 100.5, 112.0 (d, $^2J_{CF}$ = 23.9 Hz), 116.3 (d, $^2J_{CF}$ = 21.9 Hz), 116.9 (d, $^2J_{CF}$ = 21.9 Hz), 120.8 (d, $^2J_{CF}$ = 2.9 Hz), 124.1, 124.3, 124.4, 128.1, 128.5, 130.4 (d, $^4J_{CF}$ = 8.6 Hz), 130.7 (d, $^3J_{CF}$ = 8.5 Hz), 131.2 (d, $^3J_{CF}$ = 2.9 Hz), 131.4 (d, $^4J_{CF}$ = 8.5 Hz), 132.4, 143.7, 151.6, 156.3 (d, $^2J_{CF}$ = 2.7 Hz), 163.1 (d, $^1J_{CF}$ = 245.6 Hz), 164.3 (d, $^1J_{CF}$ = 251.2 Hz), 186.3; HRMS (ES):

found 438.0028. $C_{23}H_{14}O_2F_2{}^{79}Br^+$ requires 439.0067. *Anal* calcd for $C_{23}H_{13}O_2F_2Br$: C, 62.89; H, 2.98. Found: C, 62.91; H, 3.01.

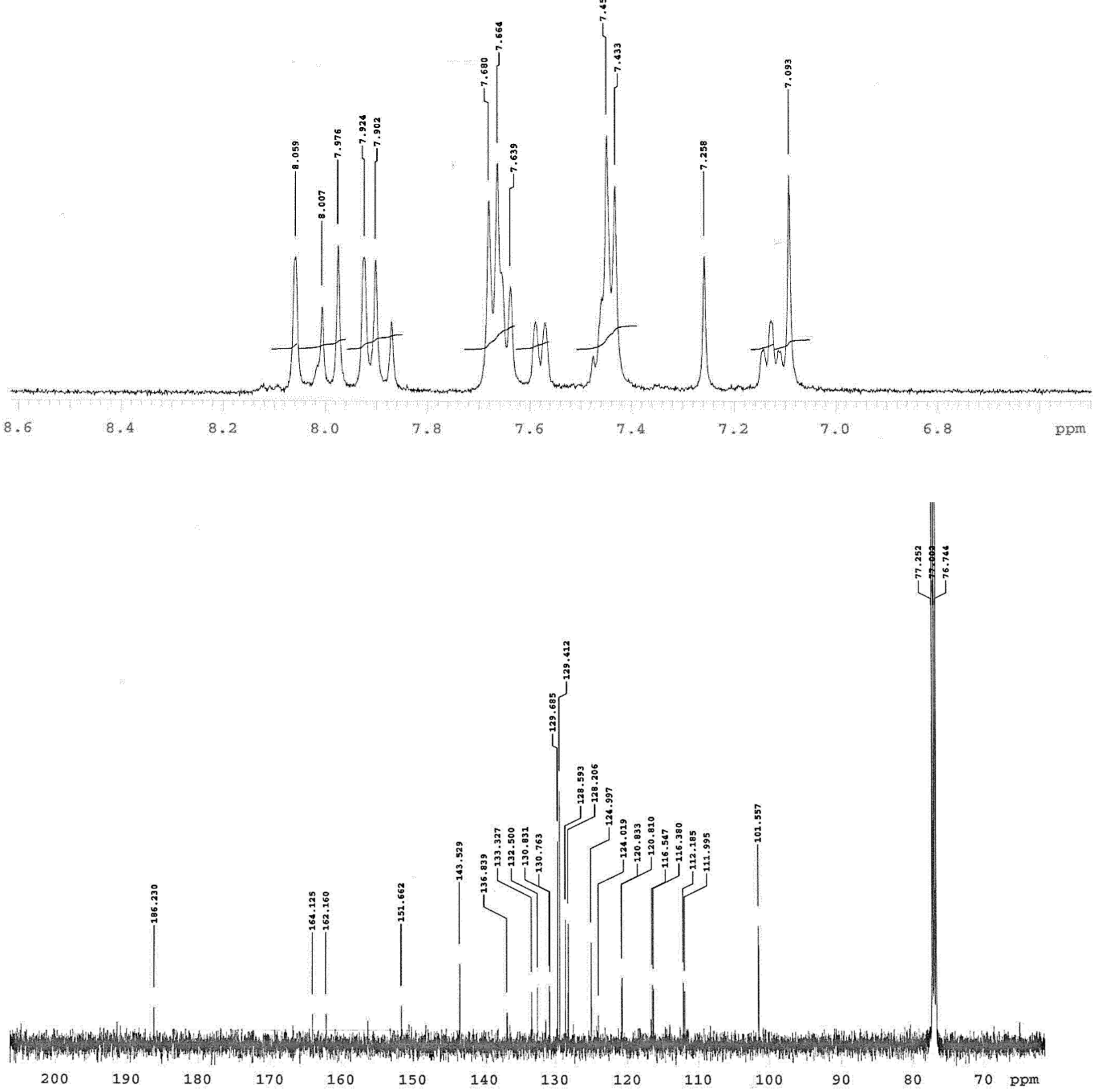

Figure S1.19: ^{1}H- and ^{13}C-NMR spectra of **3m** in $CDCl_3$ at 500 and 125 MHz, respectively.

(*E*)-1-(5-Bromo-2-(3-fluorophenyl)benzofuran-7-yl)-3-(4-chlorophenyl)prop-2-en-1-one (**3m**)

Solid (0.58 g, 68%); mp. 234–235 °C; ν_{max} (ATR) 515, 662, 728, 880, 1036, 1169, 1219, 1315, 1448, 1571, 1655 cm^{-1}; δ_H (500 MHz, $CDCl_3$) 7.09 (1H, s, =CH), 7.14 (1H, t, *J* = 8.0 Hz, Ar), 7.45 (2H, d, *J* = 8.5 Hz, Ar), 7.58 (1H, d, *J* = 8.5 Hz, Ar), 7.63-7.68 (4H, d, *J* = 7.5 Hz, Ar), 7.89 (1H, d, J_{trans} = 16.0 Hz, α-H), 7.92 (1H, d, *J* = 2.0 Hz, H-4), 7.99 (1H, d, J_{trans} = 16.0 Hz, β-H), 8.05 (1H, d, *J* = 2.0 Hz, H-6); δ_C (125 MHz, $CDCl_3$) 101.5, 112.9 (d, $^3J_{CF}$ = 23.7 Hz), 116.4 (d, $^2J_{CF}$ = 21.9 Hz), 116.7, 120.8 (d, $^2J_{CF}$ = 2.87 Hz), 124.0, 124.9, 128.2, 128.6, 129.4, 129.7, 130.8 (d, $^6J_{CF}$ = 8.5 Hz), 131.4 (d, $^4J_{CF}$ = 8.6 Hz), 132.5, 133.3, 136.8, 143.5, 151.6, 156.3 163.1 (d, $^1J_{CF}$ = 245.0 Hz), 186.2; HRMS (ES): found 454.9931 $C_{23}H_{14}O_2{}^{35}Cl^{79}BrF^+$ requires 454.9771. *Anal* calcd for $C_{23}H_{13}O_2ClBrF$: C, 60.62; H, 2.88. Found: C, 60.59; H, 2.90.

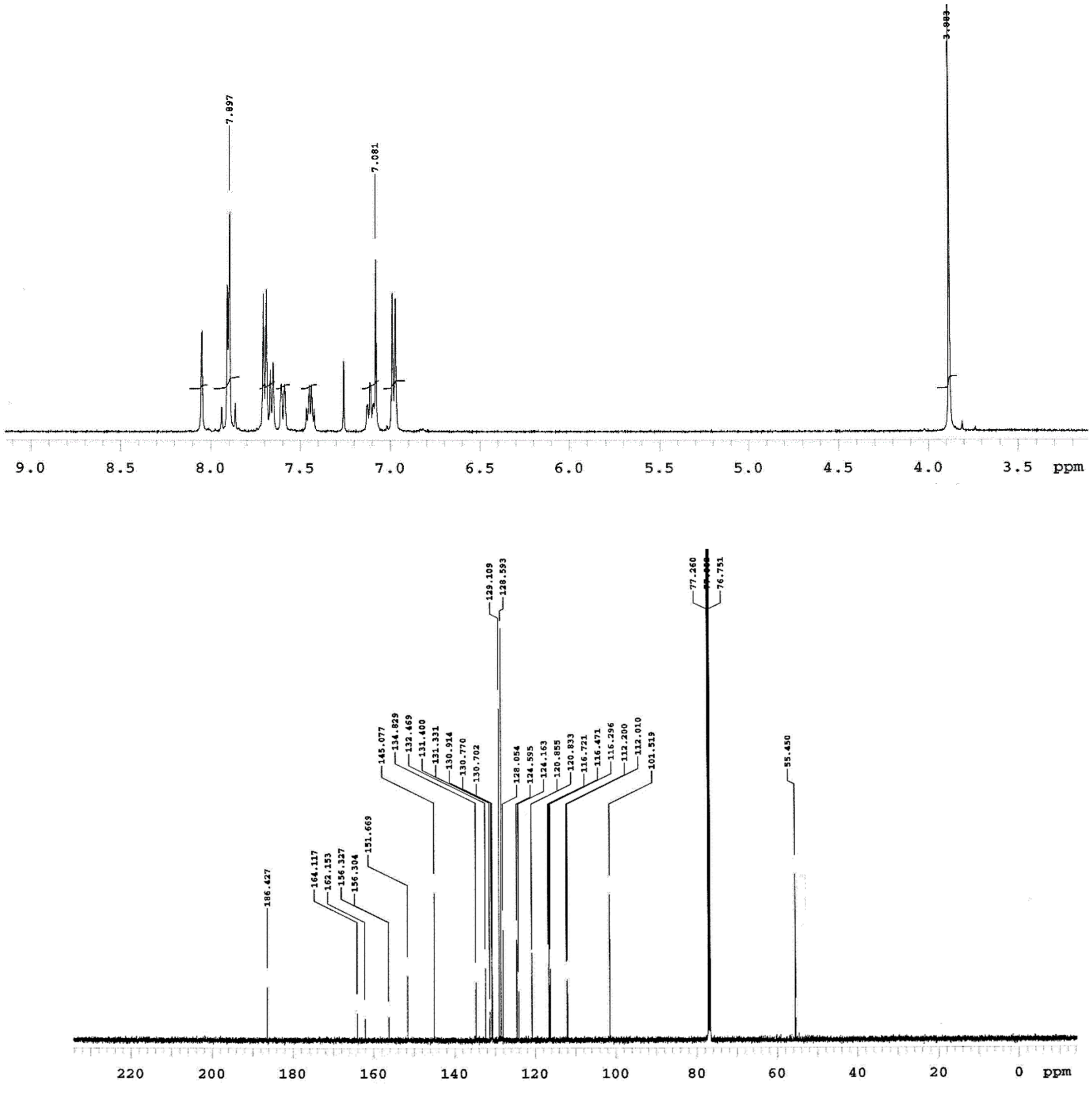

Figure S1.20: ^{1}H- and ^{13}C-NMR spectra of **3n** in $CDCl_3$ at 500 and 125 MHz, respectively.

(*E*)-1-(5-Bromo-2-(3-fluorophenyl)benzofuran-7-yl)-3-(4-methoxyphenyl)prop-2-en-1-one (**3n**)

Solid (0.79 g, 73%); mp. 221–222 °C; ν_{max} (ATR) 562, 683, 778, 893, 973, 1076, 1194, 1257, 1348, 1487, 1591, 1655 cm^{-1}; δ_H (500 MHz, $CDCl_3$) 3.88 (3H, s, OCH_3), 6.98 (2H, d, *J* = 8.5 Hz, Ar), 7.08 (1H, s, =CH), 7.11 (1H, t, *J* = 8.5 Hz, Ar), 7.45 (1H, d, *J* = 7.5 Hz, Ar), 7.69 (1H, d, *J* = 8.5 Hz, Ar), 7.65 (1H, d, *J* = 8.0 Hz, Ar), 7.69 (2H, d, *J* = 8.5 Hz, Ar), 7.87 (1H, d, J_{trans} = 16.0 Hz, α-H), 7.89 (1H, d, *J* = 1.5 Hz, H-4), 7.92 (1H, d, J_{trans} = 16.0 Hz, β-H), 8.04 (1H, d, *J* = 2.5 Hz, H-6); δ_C (125 MHz, $CDCl_3$) 55.4, 112,2 (d, $^{3}J_{CF}$ = 23.7 Hz), 114.6, 116.3 (d, $^{2}J_{CF}$ = 20.9 Hz), 116.7, 121.0 (d, $^{4}J_{CF}$ = 2.8 Hz), 122.4, 123.7, 124.5, 127.5, 127.7, 128.5, 129.9, 130.4, (d, $^{3}J_{CF}$ = 7.5 Hz),130.7, 131.4 (d, $^{3}J_{CF}$ = 7.6 Hz), 132.4, 145.0, 151.6, 156.2 (d, $^{4}J_{CF}$ = 2.9 Hz),163.1 (d, $^{1}J_{CF}$ = 263.6 Hz), 186.4; HRMS (ES): found 451.0327. $C_{24}H_{17}O_3{}^{79}Br^+$ requires 451.0345. *Anal* calcd for $C_{24}H_{16}O_3Br$: C, 63.87; H, 3.57. Found: C, 63.92; H, 3.64.

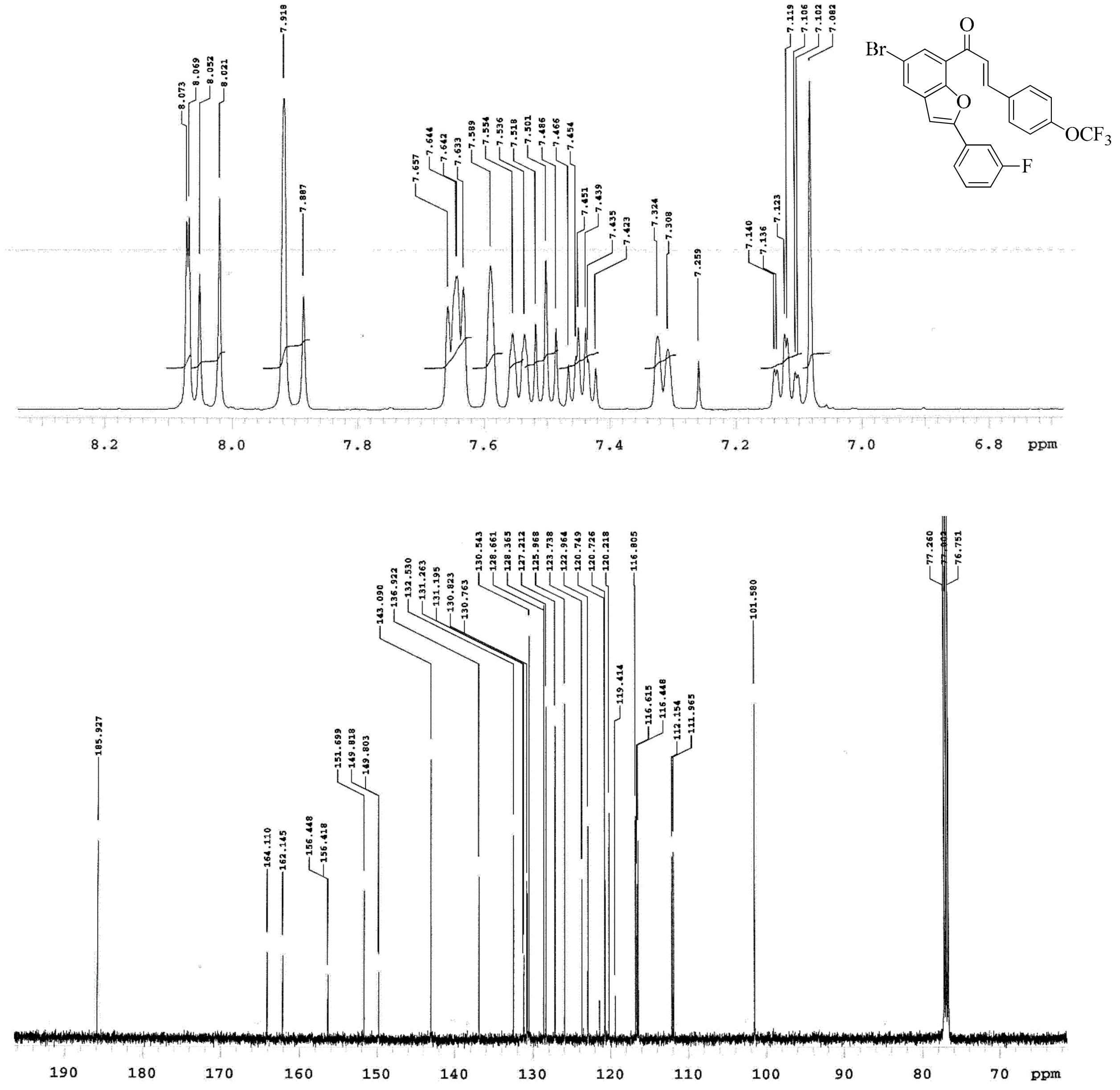

Figure S1.21: ^{1}H- and ^{13}C-NMR spectra of **3o** in $CDCl_3$ at 500 and 125 MHz, respectively.

(*E*)-1-(5-Bromo-2-(3-fluorophenyl)benzofuran-7-yl)-3-(4-(trifluoromethoxy)phenyl)prop-2-en-1-one (**3o**)

Solid (0.88 g, 87%); mp. 186–187 °C; ν_{max} (ATR) 575, 696, 779, 892, 978, 1075, 1160, 1256, 1346, 1487, 1573, 1656 cm^{-1}; δ_H (500 MHz, $CDCl_3$) 7.09 (1H, s, =CH), 7.14 (1H, t, *J* = 8.0 Hz, Ar), 7.31 (1H, d, *J* = 8.7 Hz, Ar), 7.43 (1H, d, *J* = 8.5 Hz, Ar), 7.45 (1H, d, *J* = 8.5 Hz, Ar), 7.50 (1H, t, *J* = 8.0 Hz, Ar), 7.55 (1H, dt, *J* = 8.5 and 2.5 Hz, Ar), 7.59 (1H, d, *J* = 8.0 Hz, Ar), 7.65 (1H, t, *J* = 8.5 Hz, Ar), 7.91 (1H, d, J_{trans} = 16.0 Hz, α-H), 7.93 (1H, d, *J* = 2.5 Hz, H-4) 8.04 (1H, d, J_{trans} = 16.0 Hz, β-H), 8.08 (1H, d, *J* = 1.5 Hz, H-6); δ_C (125 MHz, $CDCl_3$) 100.5, 112.0 (d, $^2J_{CF}$ = 23.9 Hz), 116.5 (d, $^2J_{CF}$ = 21.9 Hz), 116.8, 120.2 (q, J_{CF} = 256.0 Hz), 122.9, 123.7, 125.9, 127.2, 128.4, 128.7, 130.5, 130.6 (d, $^4J_{CF}$ = 7.5 Hz), 131.2 (d, $^3J_{CF}$ = 8.5 Hz), 132.5, 136.9, 143.1, 149.8 (d, $^2J_{CF}$ = 1.9 Hz), 151.7, 156.4 (d, $^2J_{CF}$ = 3.8 Hz), 163.1 (d, $^1J_{CF}$ = 245.6 Hz), 185.9; HRMS (ES): found 505.0164. $C_{24}H_{14}O_3{}^{79}BrF_4{}^+$ requires 505.0063. *Anal* calcd for $C_{24}H_{13}O_3BrF_4$: C, 57.05; H, 2.59. Found: C, 57.12; H, 2.61.

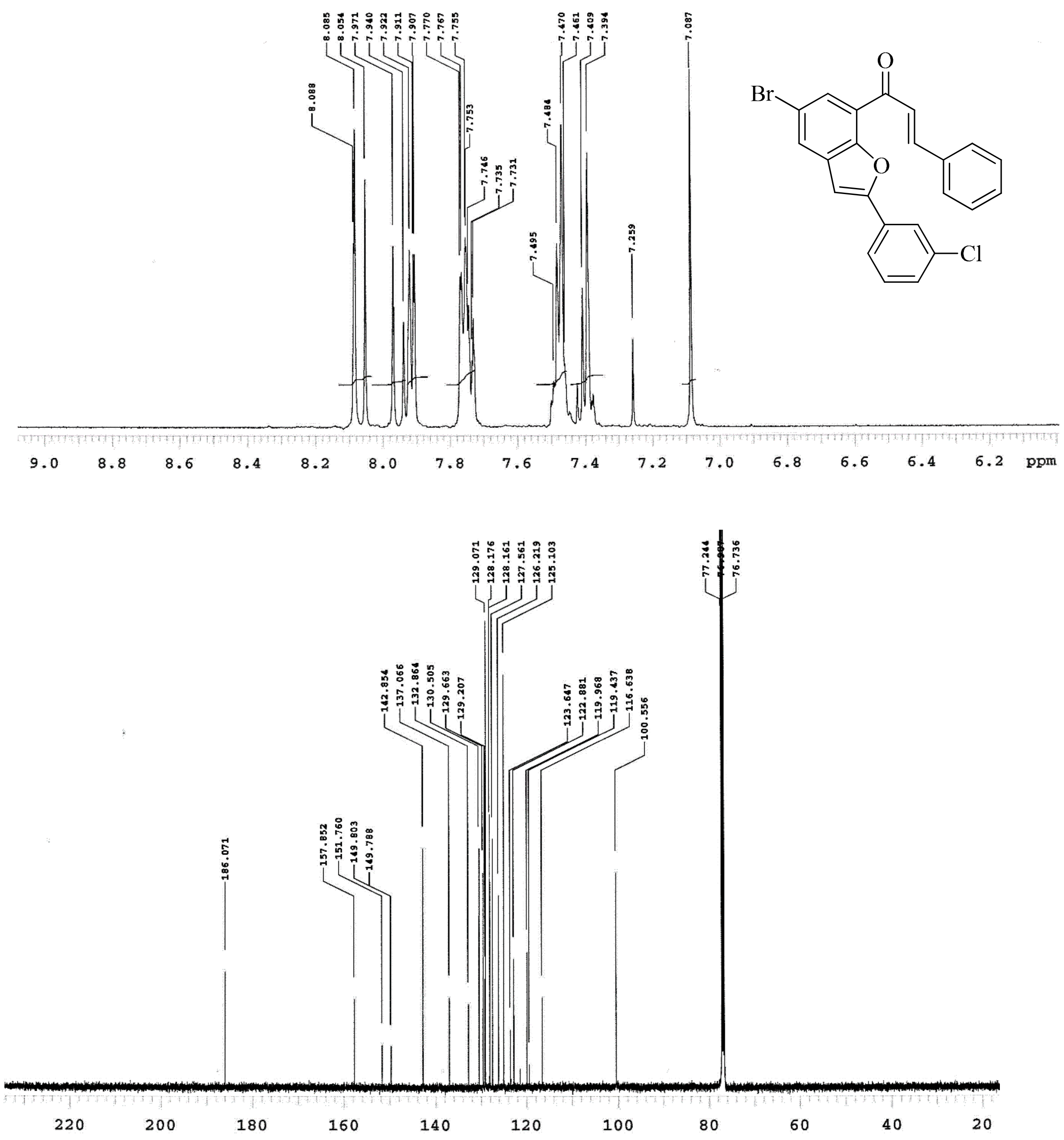

Figure S1.22: ^{1}H- and ^{13}C-NMR spectra of **3p** in $CDCl_3$ at 500 and 125 MHz, respectively.

(*E*)-1-(5-Bromo-2-(3-chlorophenyl)benzofuran-7-yl)-3-phenylprop-2-en-1-one (**3p**)

Solid (0.75 g, 75%); mp. 218–219 °C; ν_{max} (ATR) 575, 663, 780, 893, 944, 1012, 1194, 1279, 1345, 1488, 1585, 1602, 1657 cm^{-1}; δ_H (500 MHz, $CDCl_3$) 7.09 (1H, s, =CH), 7.40 (2H, d, *J* = 8.0 Hz, Ar), 7.47 (2H, d, *J* = 8.0 Hz, Ar), 7.73-7.77 (5H, m, Ar), 7.90 (1H, d, *J* = 2.5 Hz, H-4), 7.92 (1H, d, *J* = 1.5 Hz, H-6), 7.96 (1H, d, J_{trans} = 16.0 Hz, α-H), 8.06 (1H, d, J_{trans} = 16.0 Hz, β-H); δ_C (125 MHz, $CDCl_3$) 101.3, 116.4, 123.2, 124.0, 124.5, 125.1, 128.1, 128.6, 128.7, 129.2, 129.4, 130.4, 130.9, 131.0, 132.4, 134.8, 135.2, 145.1, 151.7, 156.0, 186.2; HRMS (ES): found 436.9942. $C_{23}H_{15}O_2{}^{35}Cl^{79}Br^+$ requires 436.9942. *Anal* calcd for $C_{23}H_{14}O_2ClBr$: C, 63.11; H, 3.22. Found: C, 63.08; H, 3.16.

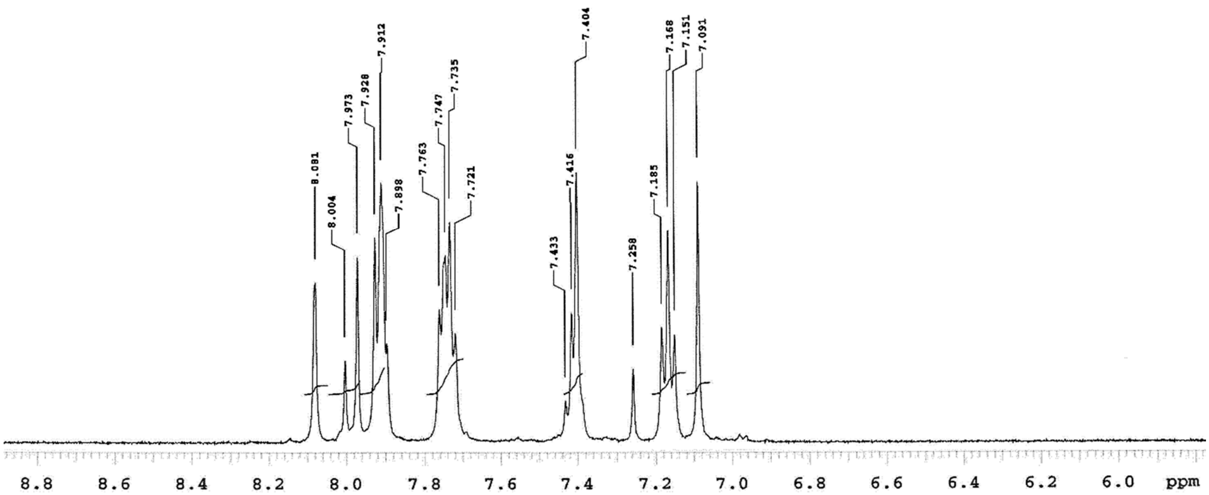

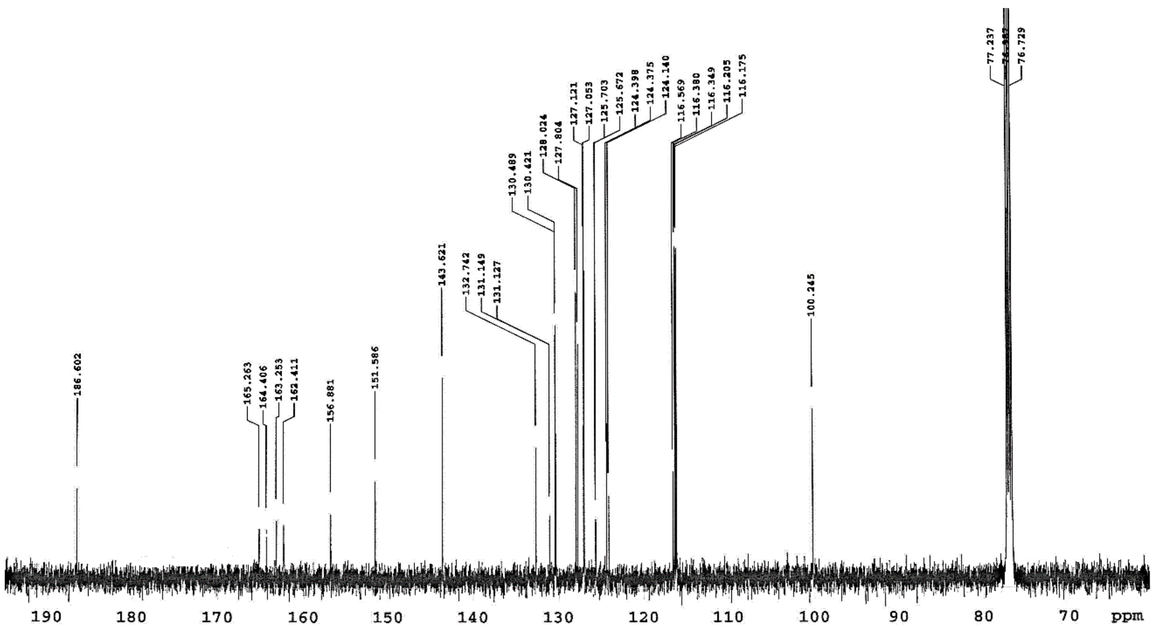

Figure S1.23: ^{1}H- and ^{13}C-NMR spectra of **3q** in $CDCl_3$ at 500 and 125 MHz, respectively.

(*E*)-1-(5-Bromo-2-(3-chlorophenyl)benzofuran-7-yl)-3-(4-fluorophenyl)prop-2-en-1-one (**3q**)

Solid (0.67 g, 69%); mp. 232–233 °C; ν_{max} (ATR) 570, 630, 781, 891, 973, 1078, 1102, 1221, 1376, 1494, 1584, 1596, 1655 cm^{-1}; δ_H (300 MHz, $CDCl_3$) 7.09 (1H, s, =CH), 7.17 (2H, t, *J* = 8.5 Hz, Ar), 7.41 (2H, t, *J* = 8.5 Hz, Ar), 7.21-7.76 (3H, m, Ar), 7.91 (1H, d, *J* = 8.0 Hz, Ar), 7.92 (1H, d *J* = 2.0 Hz, H-4), 7.95 (1H, d, J_{trans} = 16.0 Hz, α-H), 7.98 (1H, d, J_{trans} = 16.0 Hz, β-H), 8.08 (1H, d, *J* = 2.0 Hz, H-6); δ_C (75 MHz, $CDCl_3$) 100.2, 116.3 (d, $^{2}J_{CF}$ = 21.8 Hz), 116.5, 124.1, 124.3, 124.4, 125.6, 125.7, 127.1, 127.8, 128.0, 129.8, 130.4 (d, $^{3}J_{CF}$ = 8.5 Hz), 131.1 (d, $^{4}J_{CF}$ = 2.8 Hz), 132.7, 136.9, 143.6, 151.6, 156.8, 164.4 (d, $^{1}J_{CF}$ = 239.0 Hz), 186.6; HRMS (ES): found 454.9851. $C_{23}H_{14}O_2{}^{79}BrClF^{+}$ requires 454.9850. *Anal* calcd for $C_{23}H_{13}O_2BrClF$: C, 60.62; H, 2.88. Found: C, 60.64; H, 2.90.

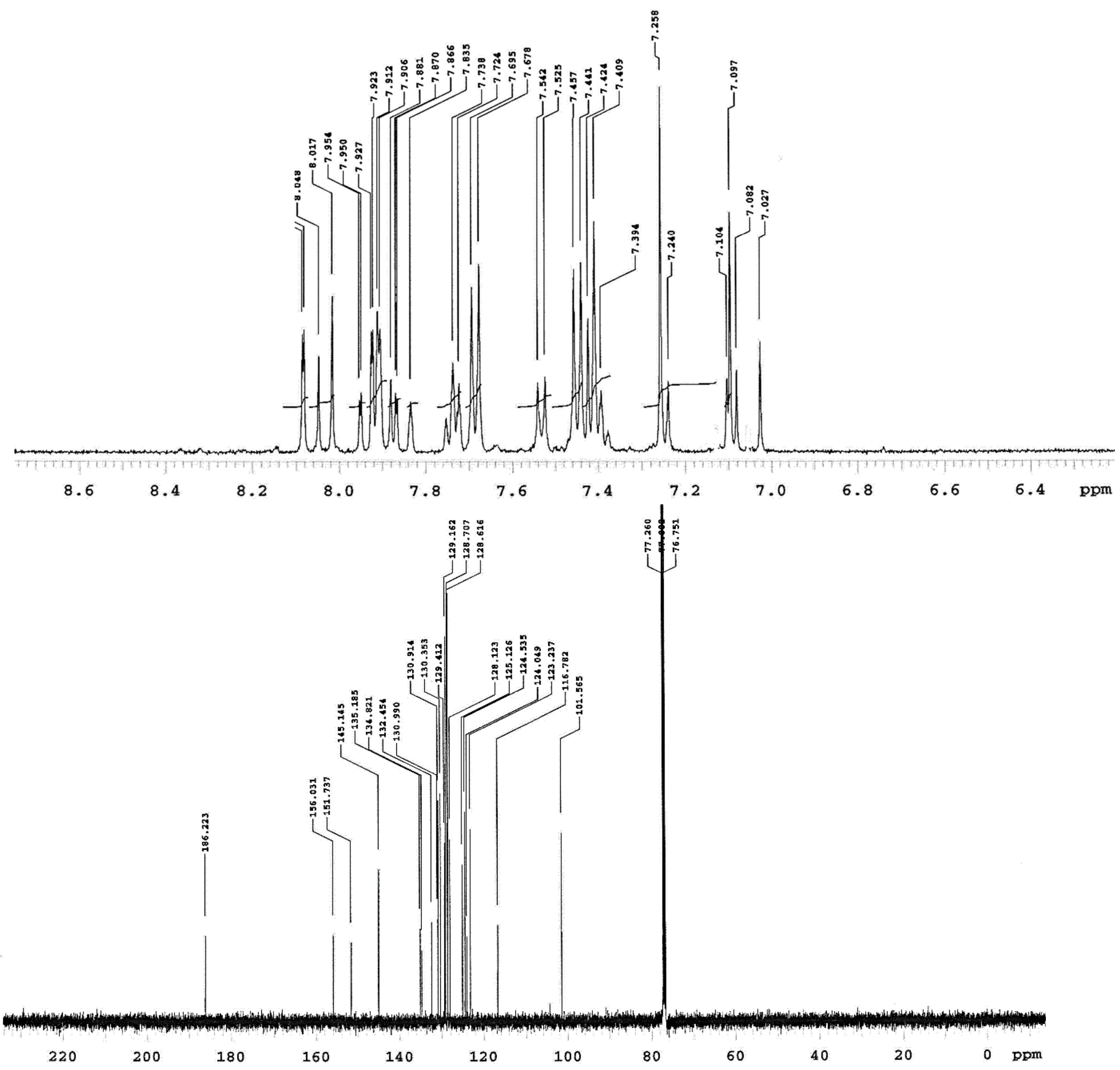

Figure S1.24: ^{1}H- and ^{13}C-NMR spectra of **3r** in $CDCl_3$ at 500 and 125 MHz, respectively.

(*E*)-1-(5-Bromo-2-(3-chlorophenyl)benzofuran-7-yl)-3-(4-chlorophenyl)prop-2-en-1-one (**3r**)

Solid (0.79, 77%); mp. 257–258 °C; ν_{max} (ATR) 574, 639, 776, 859, 980, 1077, 1192, 1238, 1346, 1476, 1507, 1562, 1656 cm^{-1}; δ_H (500 MHz, $CDCl_3$) 7.08 (1H, s, =CH), 7.41 (2H, d, *J* = 7.5 Hz, Ar), 7.46-7.48 (3H, m, Ar), 7.73-7.77 (2H, m, Ar), 7.91 (1H, d, *J* = 2.0 Hz, H-4), 7.94 (1H, d, *J* = 8.0 Hz, Ar), 7.96 (1H, d, J_{trans} = 16.0 Hz, α-H), 8.06 (1H, d, J_{trans} = 16.0 Hz, β-H), 8.08 (1H, d, *J* = 7.0 Hz, H-6); δ_C (125 MHz, $CDCl_3$) 101.6, 116.8, 123.2, 124.0, 124.5, 125.1, 128.1, 128.6, 128.7, 129.1, 129.4, 130.3, 130.9, 131.0, 132.4, 134.8, 135.2, 145.1, 151.7, 156.0, 186.2; HRMS (ES): found 454.7450. $C_{23}H_{14}O_2{}^{35}Cl_2{}^{79}Br^+$ requires 454.9850. *Anal* calcd for $C_{23}H_{13}O_2Cl_2Br$: C, 58.51; H, 2.78. Found: C, 58.60; H, 2.82.

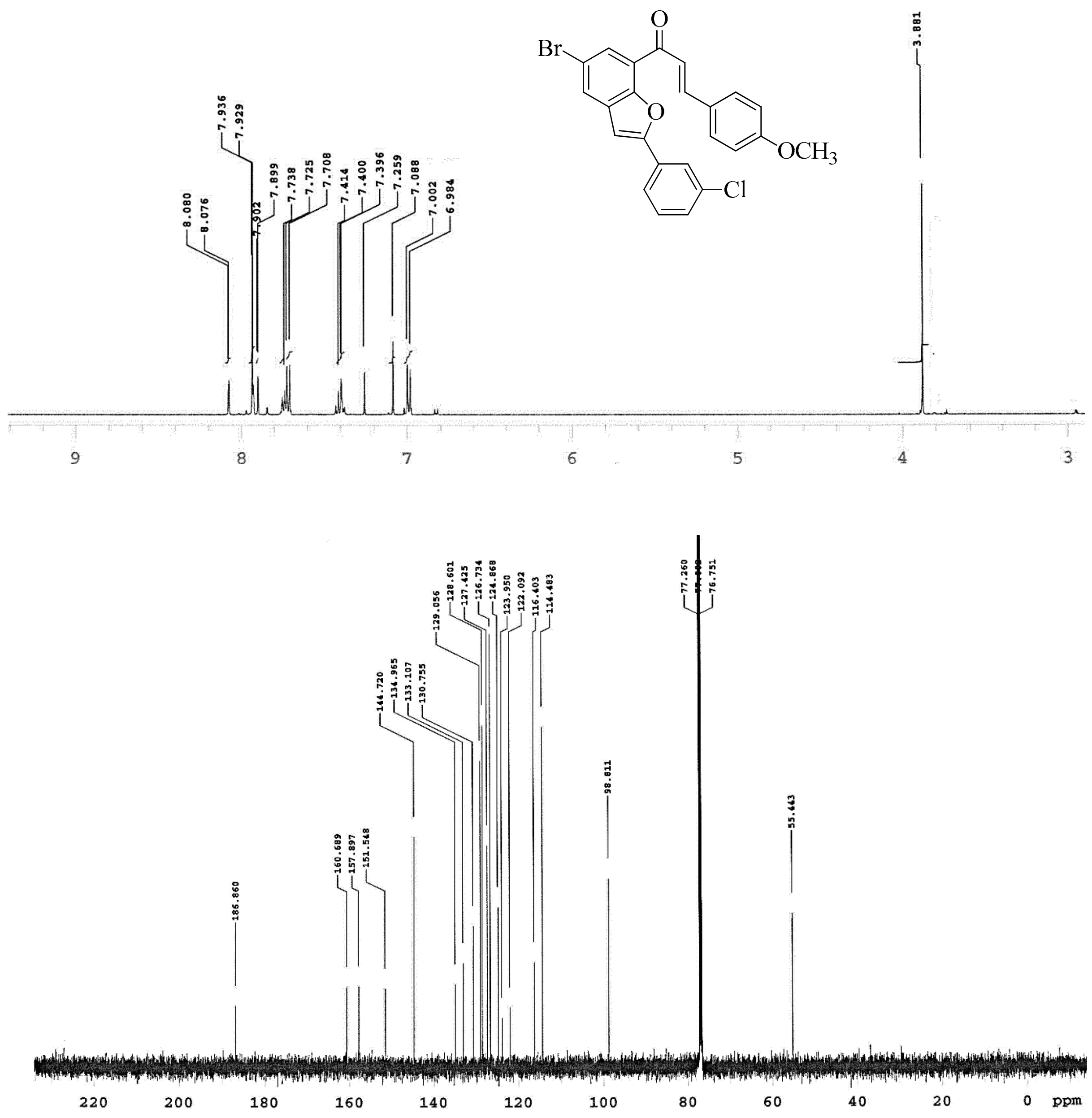

Figure S1.25: ^{1}H- and ^{13}C-NMR spectra of **3s** in $CDCl_3$ at 500 and 125 MHz, respectively.

(*E*)-1-(5-Bromo-2-(3-chlorophenyl)benzofuran-7-yl)-3-(4-methoxyphenyl)prop-2-en-1-one (**3s**)

Solid (0.65 g, 71%); mp. 214–215 °C; ν_{max} (ATR) 551, 695, 772, 893, 1076, 1169, 1255, 1346, 1474, 1508, 1585, 1654 cm^{-1}; δ_H (500 MHz, $CDCl_3$) 3.88 (3H, s, OCH_3), 6.99 (2H, d, *J* = 8.5 Hz, Ar), 7.09 (1H, s, =CH), 7.40 (2H, t, *J* = 8.0 Hz, Ar), 7.71 (2H, d, *J* = 9.0 Hz, Ar), 7.74 (2H, d, *J* = 7.5 Hz, Ar), 7.90 (1H, d, *J* = 2.5 Hz, 4-H), 8.00 (1H, d, J_{trans} = 16.0 Hz, α-H), 8.04 (1H, d, J_{trans} = 16.0 Hz, β-H), 8.06 (1H, d, *J* = 2.5 Hz, 6-H); δ_C (125 MHz, $CDCl_3$) 55.4, 101.5, 113.5, 114.6, 116.7, 122.3, 124.4, 125.1, 127.8, 128.6, 129.3, 130.3, 130.4, 131.1, 132.2, 132.4, 135.1, 145.0, 151.7, 156.0, 162.0, 186.2; HRMS MH$^+$, found 467.0128. $C_{24}H_{17}O_3{}^{79}Br^{35}Cl^+$ requires 467.0050. Anal calcd for $C_{24}H_{16}O_3BrCl$: C, 61.63; H, 3.45. Found: C, 61.58; H, 3.48.

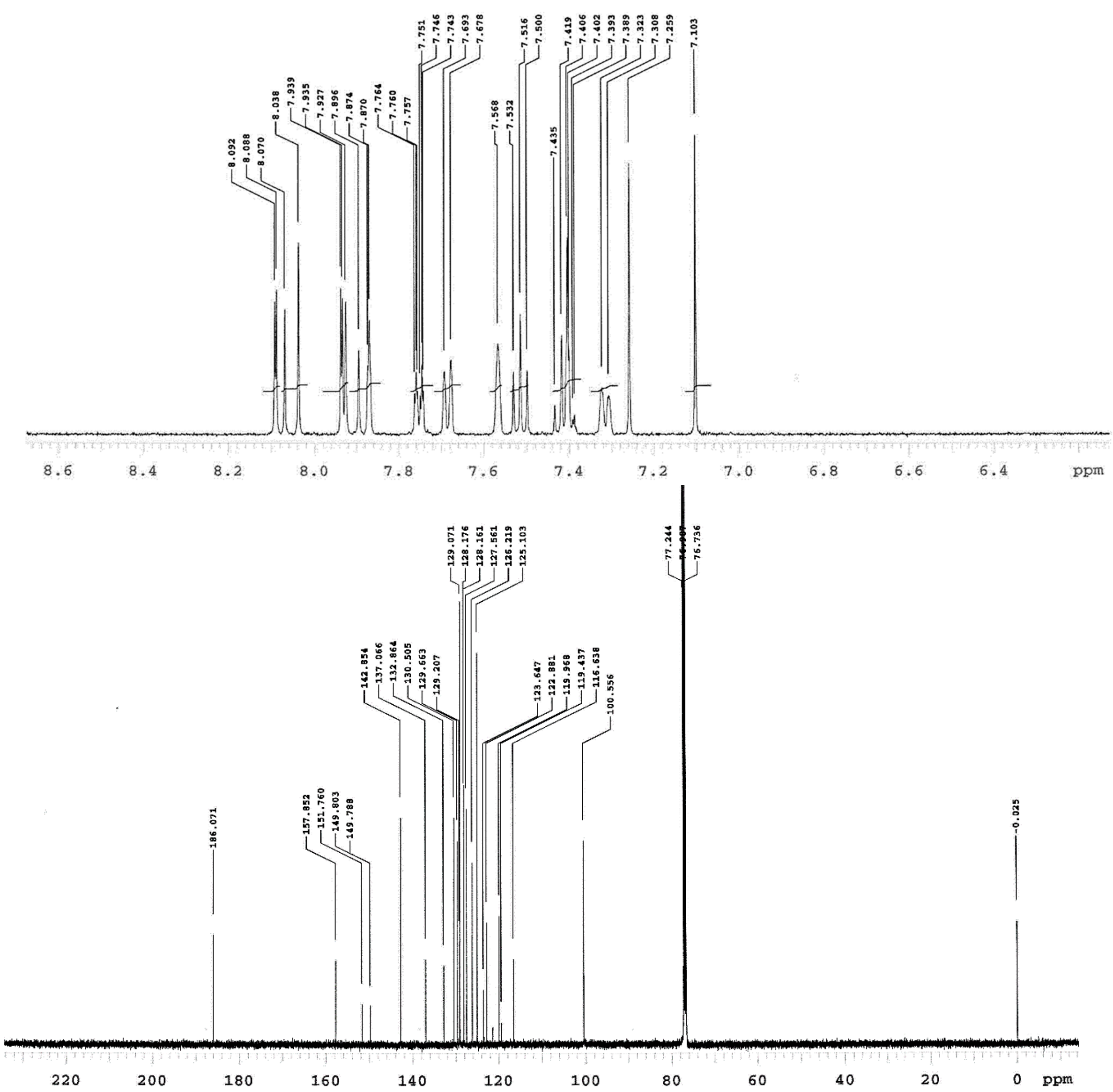

Figure S1.26: ^{1}H- and ^{13}C-NMR spectra of **3t** in $CDCl_3$ at 500 and 125 MHz, respectively.

(*E*)-1-(5-Bromo-2-(3-chlorophenyl)benzofuran-7-yl)-3-(4-(trifluoromethoxy)phenyl)prop-2-en-1-one (**3t**)

Solid (0.73 g, 71%); mp. 196–197 °C; ν_{max} (ATR) 575, 695, 776, 893, 997, 1076, 1172, 1260, 1344, 1490, 1561, 1599, 1656 cm^{-1}; δ_H (500 MHz, $CDCl_3$) 7.03 (1H, s, =CH), 7.31 (1H, d, *J* = 7.5 Hz, Ar), 7.40 (2H, d, *J* =7.0 Hz, Ar), 7.51 (1H, t, *J* = 8.0 Hz, Ar), 7.56 (1H, d, *J* = 8.0 Hz, Ar), 7.68 (1H, d, *J* =7.5 Hz, Ar). 7.75 (1H, dt, *J* = 2.0 and 8.0 Hz, Ar), 7.78 (1H, d, *J* =8.0 Hz, Ar), 7.90 (1H, d, J_{trans} = 16.0 Hz, α-H), 7.93 (1H, d, *J* 2.0 Hz, H-4), 8.05 (1H, d, J_{trans} = 16.0 Hz, β-H), 8.09 (1H, d, *J* 2.0 Hz, H-6); δ_C (125 MHz, $CDCl_3$) 101.3, 116.4, 119.5 (t, J_{CF} = 256.0 Hz), 123.2, 124.0, 124.5, 125.1, 128.1, 128.6, 128.7, 129.2, 129.4, 130.4, 130.9, 131.0, 132.4, 134.8, 135.2, 145.1, 151.7, 156.0, 186.2; HRMS (ES): found 520.9930. $C_{24}H_{14}O_3{}^{79}BrF_3{}^{35}Cl^+$ requires 520.9923. *Anal* calcd for $C_{24}H_{13}O_3BrF_3Cl$: C, 55.25; H, 2.51. Found: C, 55.30; H, 2.54.

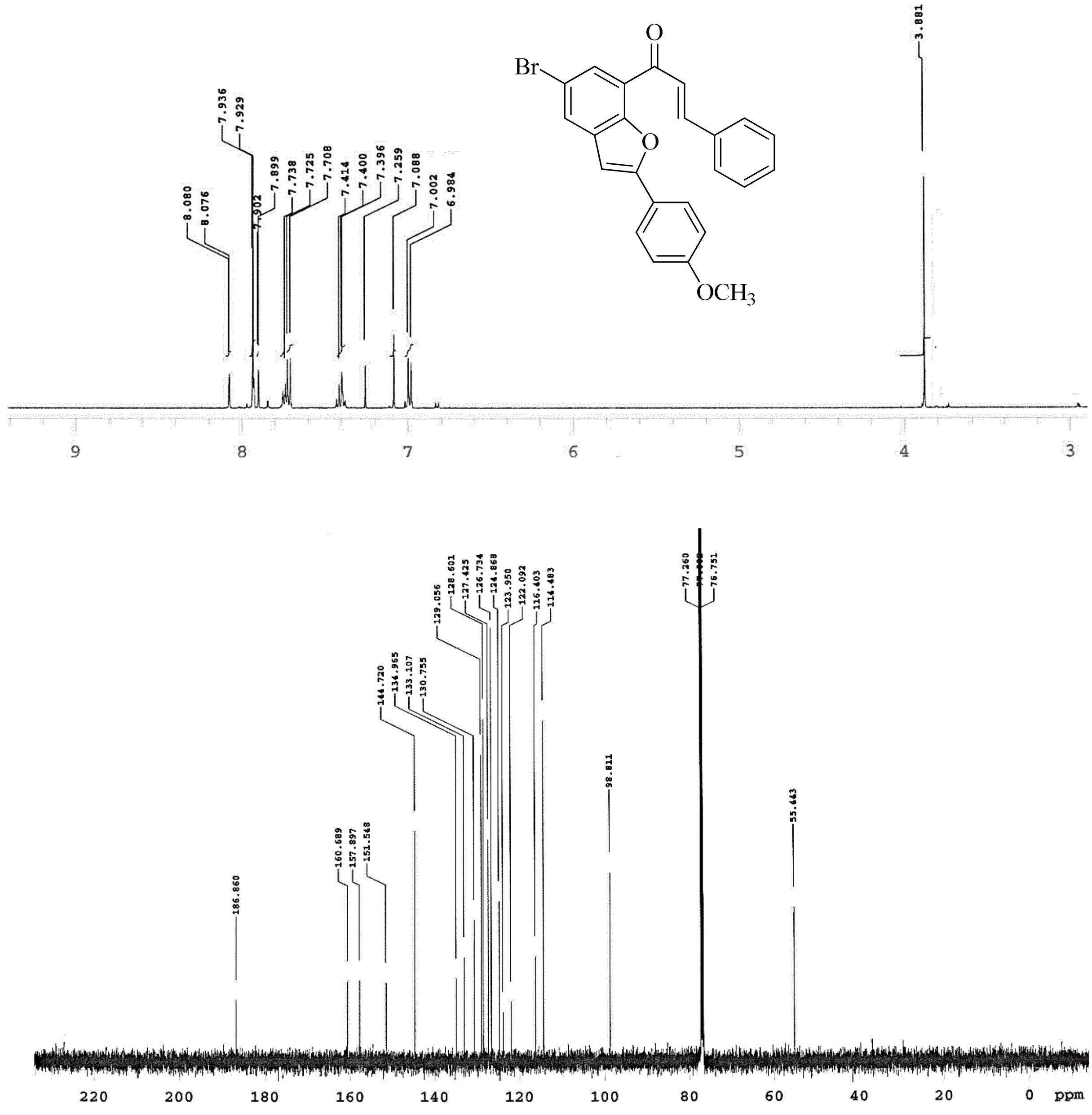

Figure S1.27: ^{1}H- and ^{13}C-NMR spectra of **3u** in $CDCl_3$ at 500 and 125 MHz, respectively.

(*E*)-1-(5-Bromo-2-(4-methoxyphenyl)benzofuran-7-yl)-3-phenylprop-2-en-1-one (**3u**)

Solid (0.68 g, 72%); mp. 255–256 °C; ν_{max} (ATR) 571, 680, 765, 889, 979, 1098, 1156, 1254, 1347, 1435, 1580, 1596, 1657 cm^{-1}; δ_H (500 MHz, $CDCl_3$) 3.87 (3H, s, OCH_3), 6.92 (1H, s, =CH), 6.98 (2H, d, *J* = 9.0 Hz, Ar), 7.45-7.46 (3H, m, Ar), 7.74 (2H, d, *J* = 8.0 Hz, Ar), 7.81 (2H, d, *J* = 9.0 Hz, Ar), 7.85 (1H, *J* = 1.5 Hz, H-4), 7.92 (1H, d, J_{trans} = 16.0 Hz, α-H), 7.98 (1H, d, *J* 2.5 Hz, H-6), 8.02 (1H, d, J_{trans} = 16.0 Hz, β-H); δ_C (125 MHz, $CDCl_3$) 55.4, 98.8, 114.4, 116.4, 122.1, 123.9, 124.9, 126.7, 127.4, 128.6, 129.1, 130.7, 133.1 (2C), 134.9, 144.7, 151.5, 157.9, 160.5, 186.8; HRMS (ES): found 433.0439. $C_{24}H_{18}O_3{}^{79}Br^+$ requires 433.0439 *Anal* calcd for $C_{24}H_{17}O_3Br$: C, 66.53; H, 3.95. Found: C, 66.54; H, 3.95.

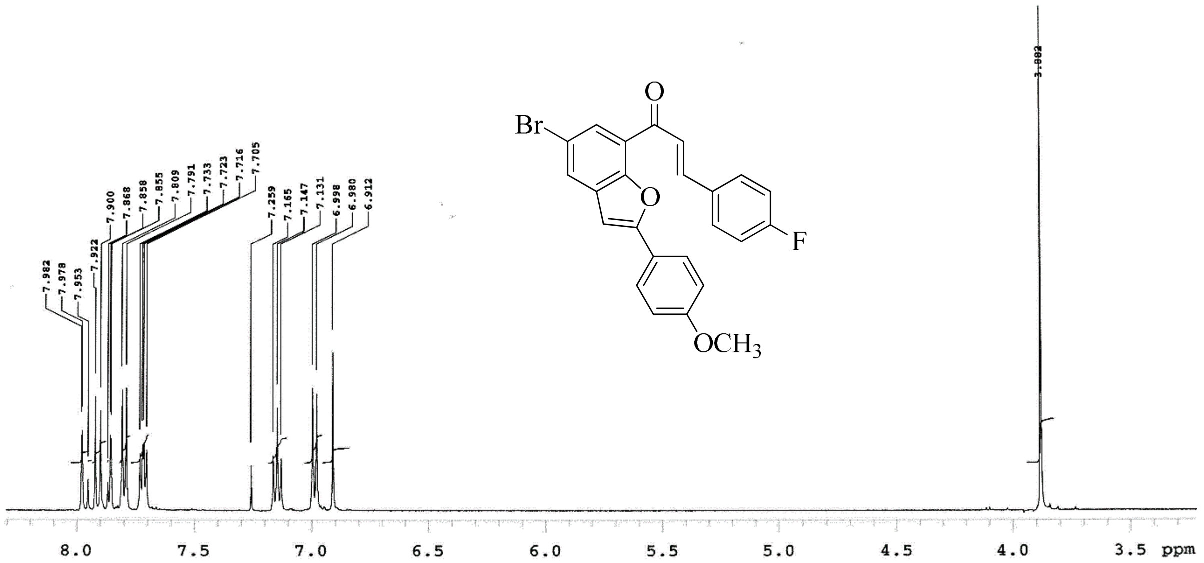

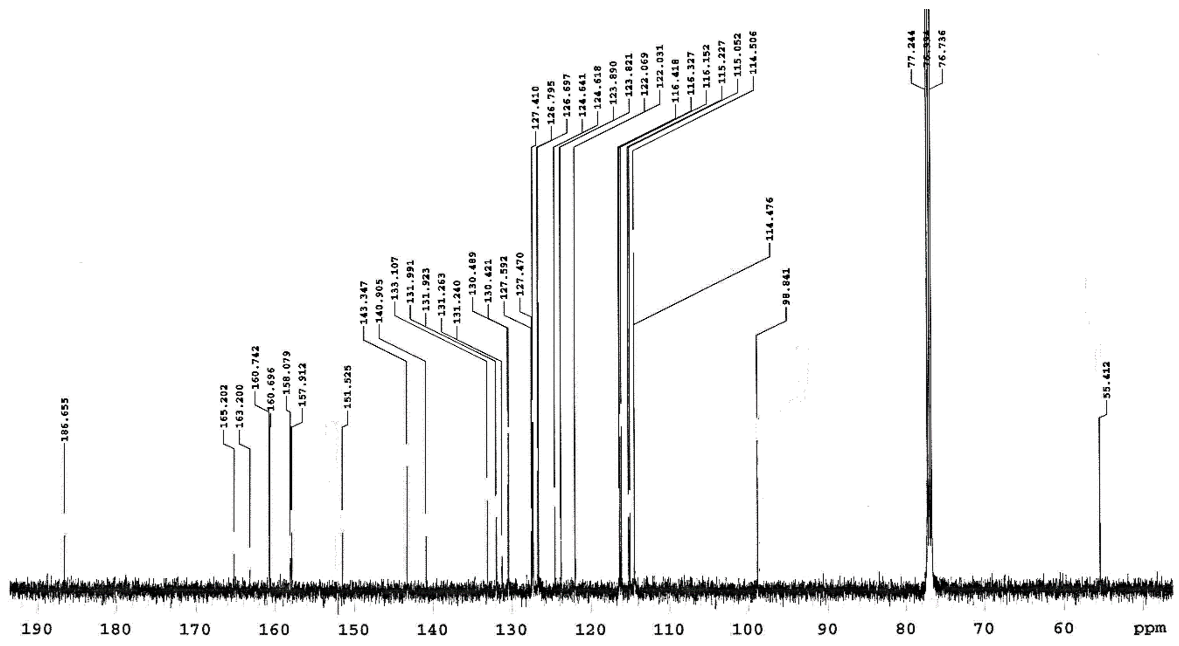

Figure S1.28: ^{1}H- and ^{13}C-NMR spectra of **3v** in $CDCl_3$ at 500 and 125 MHz, respectively.

(*E*)-1-(5-Bromo-2-(4-methoxyphenyl)benzofuran-7-yl)-3-(4-fluorophenyl)prop-2-en-1-one (**3v**)

Solid (0.78 g, 82%); mp. 241–242 °C; ν_{max} (ATR) 575, 695, 776, 893, 997, 1076, 1172, 1260, 1344, 1490, 1561, 1599, 1656 cm^{-1}; δ_H (500 MHz, $CDCl_3$) 6.91 (1H, s, =CH), 6.98 (2H, d, *J* = 9.0 Hz, Ar), 7.14 (2H, t, *J* = 8.5 Hz, Ar), 7.72 (2H, t, *J* = 8.5 Hz, Ar), 7.79 (2H, d, *J* = 9.0 Hz, Ar), 7.85 (1H, d *J* = 1.5 Hz, H-4), 7.90 (1H, d, J_{trans} = 16.0 Hz, α-H), 7.93 (1H, d, J_{trans} = 16.0 Hz, β-H), 7.98 (1H, d, *J* = 2.4 Hz, H-6); δ_C (125 MHz, $CDCl_3$) 55.5, 100.2, 114.6, 116.3 (d, $^2J_{CF}$ = 21.9 Hz), 124.1, 124.3, 124.4, 125.8, 127.0, 127.6, 128.8, 130.4 (d, $^3J_{CF}$ = 8.7 Hz), 131.2, 132.4 (d, $^4J_{CF}$ = 3.8 Hz), 143.6, 144.9, 151.5, 156.8, 161.6 (d, $^1J_{CF}$ = 245.0 Hz), 186.8; HRMS (ES): found 451.0333. $C_{24}H_{17}O_3{}^{79}BrF^+$ requires 451.0345. *Anal* calcd for $C_{24}H_{16}O_3{}^{79}BrF$: C, 63.87; H, 3.57. Found: C, 63.89; H, 3.62.

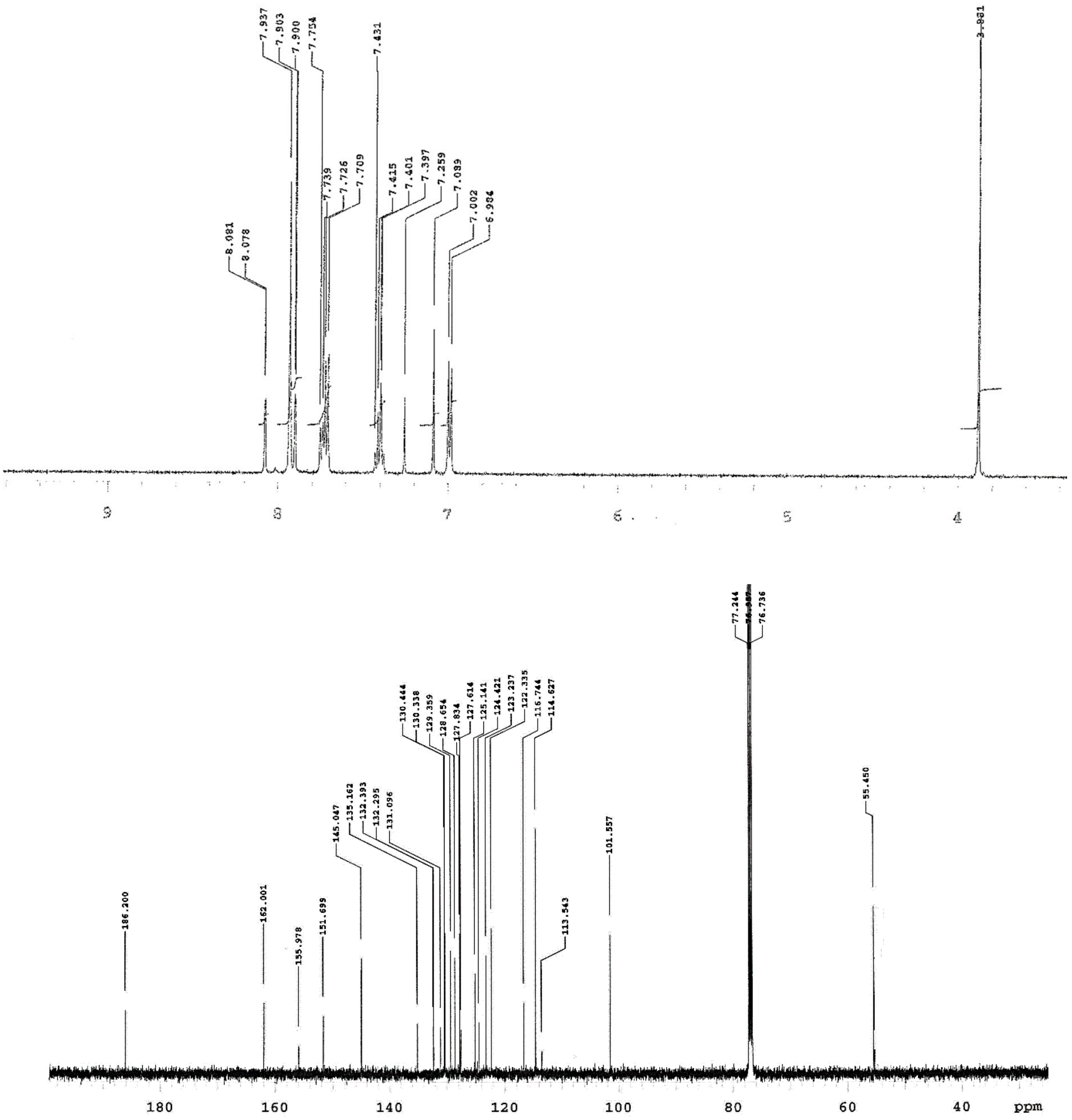

Figure S1.29: ^{1}H- and ^{13}C-NMR spectra of **3w** in $CDCl_3$ at 500 and 125 MHz, respectively.

(*E*)-1-(5-Bromo-2-(4-methoxyphenyl)benzofuran-7-yl)-3-(4-chlorophenyl)prop-2-en-1-one (**3w**)

Solid (0.83, 79%); mp. 209–210 °C; ν_{max} (ATR) 550, 646, 784, 982, 1098, 1173, 1254, 1346, 1436, 1579, 1657 cm^{-1}; δ_H (500 MHz, $CDCl_3$) 3.88 (3H, s, OCH_3), 6.98 (2H, d, *J* = 9.0 Hz, Ar), 7.08 (1H, s, =CH), 7.40 (2H, d, *J* = 8.0 Hz, Ar), 7.72 (2H, d, *J* = 9.0 Hz, Ar), 7.75 (2H, d, *J* = 8.5 Hz, Ar), 7.90 (1H, d, *J* = 2.5 Hz, 4-H), 7.92 (1H, d, J_{trans} = 16.0 Hz, α-H), 7.97 (1H, d, J_{trans} = 16.0 Hz, β-H), 8.08 (1H, d, *J* = 2.5 Hz, 6-H); δ_C (125 MHz, $CDCl_3$) 55.4, 101.6, 114.6, 116.7, 122.3, 123.2, 124.4, 125.1, 127.6, 129.4, 130.3, 130.4, 131.0, 132.3, 135.1, 145.0, 151.7, 155.9, 162.0, 186.2; HRMS (ES): found 466.381. $C_{24}H_{17}O_3{}^{35}Cl^{79}Br^+$ requires 466.0377. *Anal* calcd for $C_{24}H_{16}O_3ClBr$: C, 61.63; H, 3.45. Found: C, 61.65; H, 3.47.

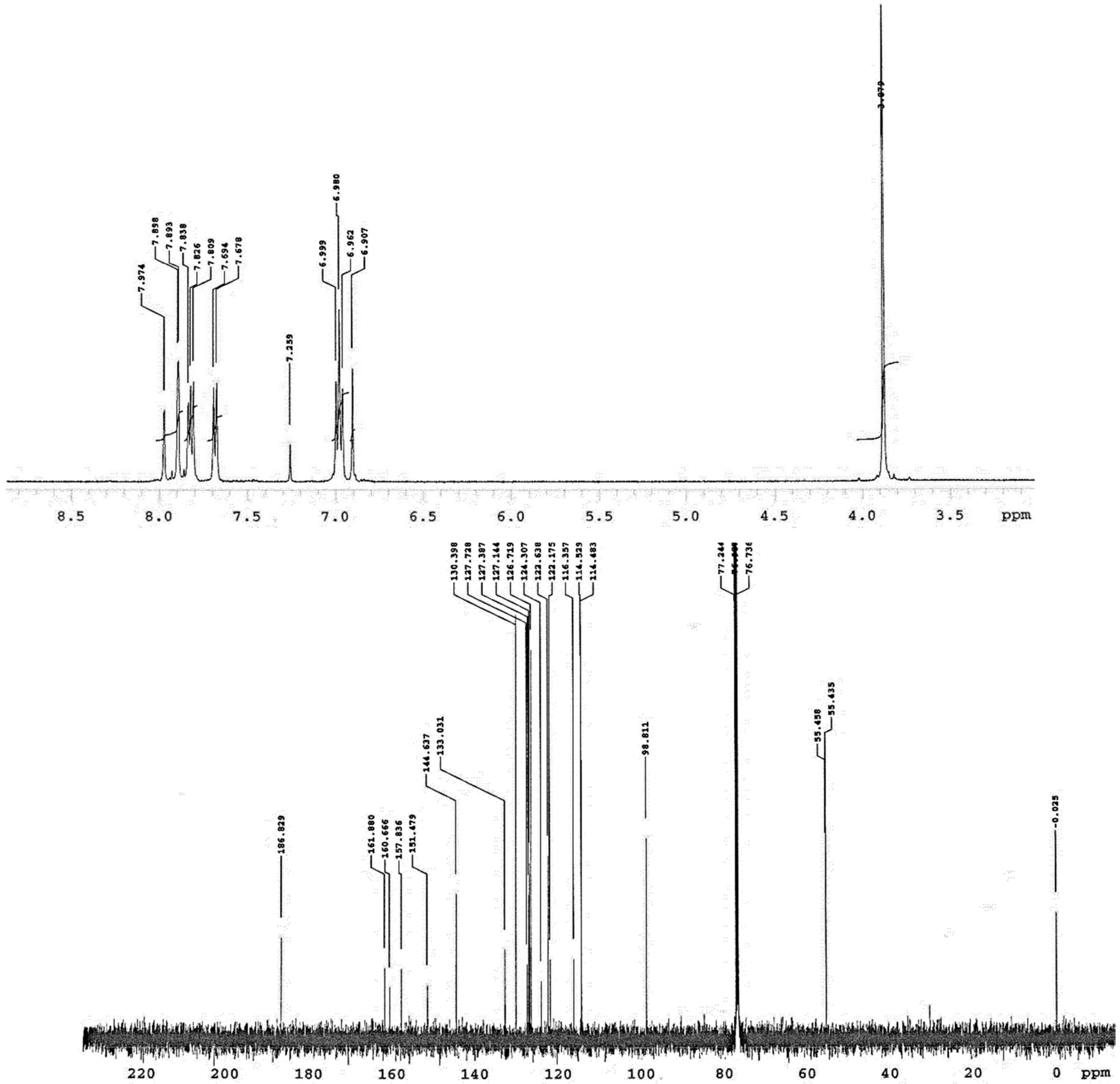

Figure S1.30: ^{1}H- and ^{13}C-NMR spectra of **3x** in $CDCl_3$ at 500 and 125 MHz, respectively.

(*E*)-1-(5-Bromo-2-(4-methoxyphenyl)benzofuran-7-yl)-3-(4-methoxyphenyl)prop-2-en-1-one (**3x**)

Solid (0.64 g, 69%); mp. 216–217 °C; ν_{max} (ATR) 550, 699, 783, 980, 1093, 1213, 1346, 1506, 1585, 1657 cm^{-1}; δ_H (500 MHz, $CDCl_3$) 3.87 (6H, s, 2xOCH_3), 6.91 (1H, s, =CH), 6.97 (2H, d, *J* = 9.0 Hz, Ar), 6.98 (2H, d, *J* = 9.0 Hz, Ar), 7.68 (2H, d, *J* = 8.0 Hz, Ar), 7.81 (2H, d, *J* = 8.5 Hz, Ar), 7.83 (1H, d, *J* = 8.0 Hz, Ar), 7.84 (1H, d, J_{trans} = 16.0 Hz, α-H), 7.87 (1H, d, J_{trans} = 16.0 Hz, β-H), 7.89 (1H, d, *J* = 2.5 Hz, 4-H), 7.97 (1H, d, *J* = 2.5 Hz, 6-H); δ_C (125 MHz, $CDCl_3$) 55.4, 55.5, 98.8, 114.4, 114.5,116.3, 122.2, 122.6, 124.3, 126.7, 127.1, 127.3, 130.4, 133.0, 144.6, 151.5, 157.8, 160,7, 161.9, 186.8; HRMS MH$^+$, found 463.0532. $C_{25}H_{20}O_4{}^{79}Br^+$ requires 463.0545. *Anal* calcd for $C_{25}H_{19}O_4Br$: C, 64.81; H, 4.13. Found: C, 64.78; H, 4.20.

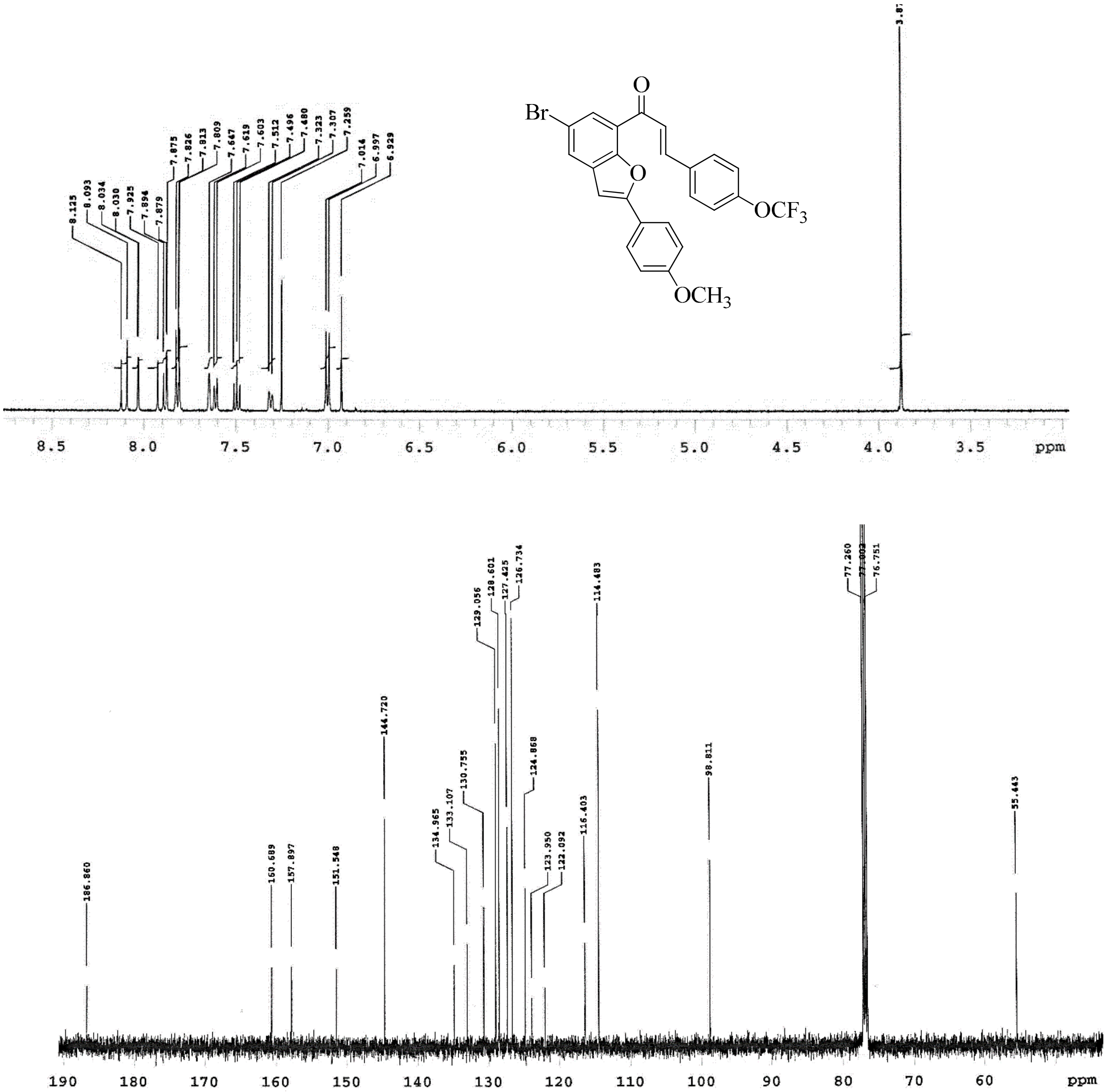

Figure S1.31: ^{1}H- and ^{13}C-NMR spectra of **3y** in $CDCl_3$ at 500 and 125 MHz, respectively.

(*E*)-1-(5-Bromo-2-(4-methoxyphenyl)benzofuran-7-yl)-3-(4-(trifluoromethoxy)phenyl)prop-2-en-1-one (**3y**)

Solid (0.59 g, 67%); mp. 187–188 °C; ν_{max} (ATR) 549, 694, 797, 866, 999, 1023, 1171, 1248, 1349, 1481, 1569, 1598, 1655 cm^{-1}; δ_H (500 MHz, $CDCl_3$) 3.87 (3H, s, OCH_3), 6.93 (1H, s, =CH), 7.00 (2H, d, *J* = 8.5 Hz), 7.31 (1H, d, *J* = 8.0 Hz), 7.49 (1H, t, *J* = 8.0 Hz, Ar), 7.61 (1H, d, *J* = 8.0 Hz, Ar), 7.65 (1H, d, *J* = 8.0 Hz, Ar), 7.81 (2H, d, *J* = 8.5 Hz, Ar), 7.87 (1H, *J* = 2.0 Hz, H-4), 7.91 (1H, d, J_{trans} = 16.0 Hz, α-H), 8.03 (1H, d, *J* 2.0 Hz, H-6), 8.11 (1H, d, J_{trans} = 16.0 Hz, β-H); δ_C (125 MHz, $CDCl_3$) 55.4, 98.9, 114.5, 116.5, 119.5 (t, J_{CF} = 256.0 Hz), 119.8, 121.9, 122.8, 123.4, 126.3, 126.6, 127.5, 127.7, 127.8, 130.5, 133.2, 139.4, 142.7, 158.0, 160.8, 186.2; HRMS (ES): found 517.0262. $C_{25}H_{17}O_4{}^{79}BrF_3{}^{+}$ requires 517.0262. *Anal* calcd for $C_{25}H_{16}O_4BrF_3$: C, 58.05; H, 3.12. Found: C, 58.06; H, 3.13.

Table S1: IC_{50} values of **3a–y** against tubulin using colchicine as a positive control.

**3a–y**	**IC_{50} (μM)**	**SD**
**3a**	15.6	± 0.06
**3b**	26.5	± 0.17
**3c**	26.4	± 0.27
**3d**	34.5	± 0.22
**3e**	25.4	± 0.24
**3f**	23.3	± 0.11
**3g**	13.2	± 0.17
**3h**	1.33	± 0.38
**3i**	5.51×10^{-5}	± 0.39
**3j**	8.85×10^{-3}	± 0.42
**3k**	77.0	± 0.21
**3l**	3.98	± 0.07
**3m**	10.2	± 0.19
**3n**	0.8×10^{-1}	± 0.37
**3o**	1.76×10^{-4}	± 0.11
**3p**	9.37×10^{-2}	± 0.21
**3q**	51.3	± 0.22
**3r**	34.5	± 0.16
**3s**	0.71	± 0.30
**3t**	2.83	± 0.07
**3u**	18.3×10^{-2}	± 0.16
**3v**	1.62×10^{-2}	± 0.12
**3w**	48.4	± 0.11
**3x**	67.4	± 0.14
**3y**	78.0	± 0.07
**Colchicine**	9.88×10^{-2}	± 0.17
